# Hydroxyl carboxylate anion catalyzed depolymerization of biopolyesters and transformation to chemicals†

**DOI:** 10.1039/d4sc02533d

**Published:** 2024-06-17

**Authors:** Yanfei Zhao, Hui Zhang, Fengtian Wu, Rongxiang Li, Minhao Tang, Yusi Wang, Wei Zeng, Buxing Han, Zhimin Liu

**Affiliations:** a Beijing National Laboratory for Molecular Sciences, CAS Laboratory of Colloid and Interface and Thermodynamics Department, CAS Research/Education Center for Excellence in Molecular Sciences, Center for Carbon Neutral Chemistry, Institute of Chemistry, Chinese Academy of Sciences Beijing 100190 China wufengtian123@126.com liuzm@iccas.ac.cn; b Jiangxi Province Key Laboratory of Polymer Micro/Nano Manufacturing and Devices, Jiangxi Province Key Laboratory of Synthetic Chemistry, East China University of Technology Economic Development Zone, Guanglan Avenue 418 Nanchang 330013 China; c University of Chinese Academy of Sciences Beijing 100049 China

## Abstract

Upcycling biopolyesters (*e.g.*, polyglycolic acid, PGA) into chemicals is an interesting and challenging topic. Herein, we report a novel protocol to upgrade biopolyesters derived from hydroxyl carboxylic acids over ionic liquids with a hydroxyl carboxylate anion (*e.g.*, glycolate, lactate) into various chemicals under metal-free conditions. It is found that as hydrogen-bond donors and acceptors, hydroxyl carboxylate anions can readily activate the ester group *via* hydrogen bonding and decompose biopolyesters *via* autocatalyzed-transesterification to form hydroxyl carboxylate anion-based intermediates. These intermediates can react with various nucleophiles (*e.g.* H_2_O, methanol, amines and hydrazine) to access the corresponding acids, esters and amides under mild conditions (*e.g.*, 40 °C). For example, 1-ethyl-3-methylimidazolium glycolate can achieve complete transformation of PGA into various chemicals such as glycolic acid, alkyl glycolates, 2-hydroxy amides, 2-(hydroxymethyl)benzimidazole, and 1,3-benzothiazol-2-ylmethanol in excellent yields *via* hydrolysis, alcoholysis and aminolysis, respectively. This protocol is simple, green, and highly efficient, which opens a novel way to upcycle biopolyesters to useful chemicals.

## Abstract 2

Hydroxyl carboxylate-based ionic liquids can achieve degradation of biopolyesters derived from hydroxyl carboxylic acids into various chemicals under mild and metal-free conditions.

## Graphical abstract

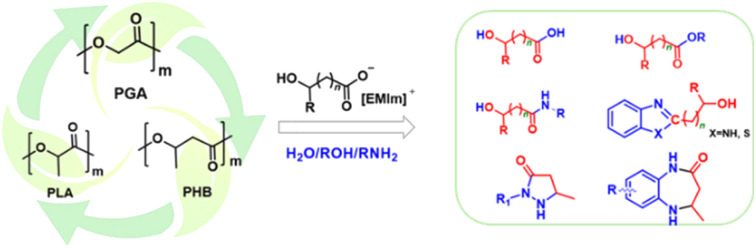

## Introduction

Plastics are ubiquitous and indispensable in our society due to their low cost, great versatility, easy applicability and high tunability, which have revolutionized modern life.^1,2^ However, a large accumulation of end-of-life plastics has caused a disruptive impact on the environment and ecosystem.^3,4^ Recently, biodegradable polymers derived from hydroxyl carboxylic acids, such as polyglycolic acid (PGA), polylactic acid (PLA) and poly-β-hydroxybutyrate (PHB), have emerged as green alternatives to petroleum-based plastics, which have received considerable attention.^5–8^ Unfortunately, their complete degradation is very sluggish with the release of CO_2_ in the natural environment, which not only increases carbon emissions but also squanders valuable carbon resources.^9,10^ In this context, chemical recycling of waste biopolymers into useful chemicals/feedstocks offers attractive access to achieve plastic sustainability. State-of-the-art approaches for polyester valorization mainly involve pyrolysis,^11^ hydrolysis,^12^ methanolysis,^13^ acetolysis,^14^ ammonolysis,^15^ hydrogenolysis^16^ and hydrosilylation,^17^ which produce corresponding carboxylic acids, esters, amides, alcohols, *etc.* Though much progress has been made, it is still highly required to develop a simple, effective and sustainable way to recycle discarded polyesters to useful chemicals.

Ionic liquids (ILs) are typically composed of organic cations and organic or inorganic anions, which possess many fascinating and tunable properties, making them widely applied in various fields. In particular, ILs can be endowed with specific functions, such as acidity/basicity, hydrophilicity/hydrophobicity, nucleophilicity, reactivity, and coordinating ability, *via* judicious selection of cations and anions, which have achieved various chemical reactions under metal-free and mild conditions.^18–23^ For instance, lactate-based ILs could achieve aminolysis of PLA and polycarbonate with aromatic amines into corresponding *N*-aryl lactamides and aryl ureas, respectively.^15^ The chloroaluminate ILs could catalyze the decomposition of polyethylene into liquid isoalkanes (C6 to C10) at temperatures below 100 °C.^2^ The ILs with halide anions could catalyze the breakage of the C_alkoxy_–O bond in polyesters, thus in combination with Pd/C accomplishing the transformation of diverse polyesters into corresponding carboxylic acids and alkanes in the presence of H_2_.^16^

N-Heterocyclic amides, such as benzimidazoles, benzothiazoles, benzothiazinones, pyrazolidines and benzodiazepines, represent key structural motifs in pharmaceutically important N-containing compounds, and often serve as attractive synthetic targets in organic synthesis, life science, agrochemical industry and functional materials. They can be produced from the reactions of desired amines with hydroxyl carboxylic acids and their derivatives,^24–27^ but suffer from their inherent shortcomings. Considering the structures of biopolyesters derived from self-condensation of hydroxyl carboxylic acids, they may serve as carbonylation feedstocks for synthesis of N-heterocyclic amides, which is however seldom reported.

**Scheme 1:**
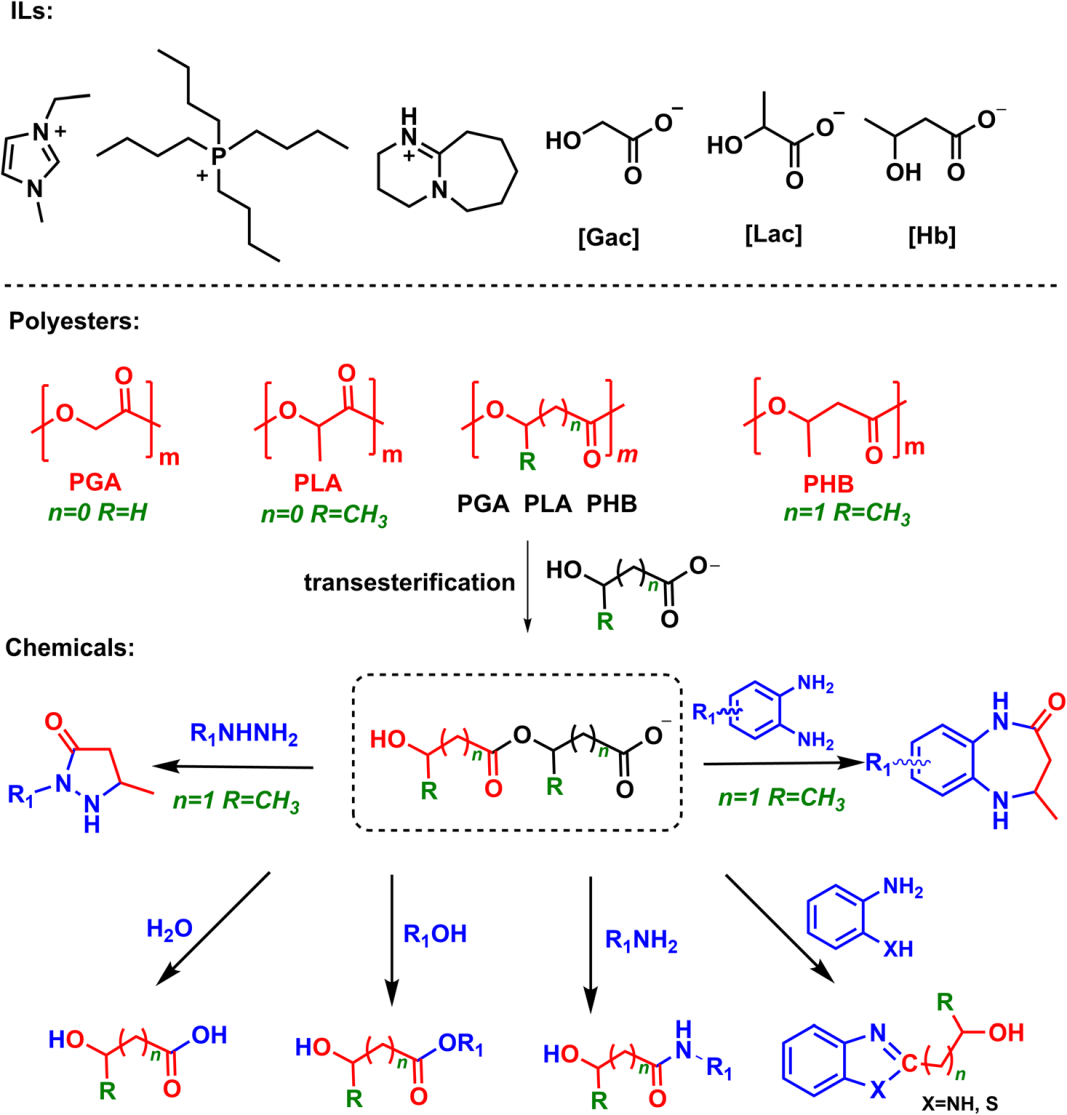
Chemical structure of hydroxyl carboxylate ILs and upcycling polyesters to various chemicals.

Herein, we report a novel and general protocol to depolymerize biopolyesters derived from hydroxyl carboxylic acids into various chemicals over hydroxyl carboxylate ILs under metal-free and mild conditions (Scheme 1). It is found that as hydrogen-bond donors and acceptors, hydroxyl carboxylate anions (*e.g.*, glycolate, lactate) can readily activate the ester group *via* hydrogen bonding, and further decompose biopolyesters *via* autocatalyzed-transesterification to form hydroxyl carboxylate anion-based intermediates. These intermediates are able to react with various nucleophiles (*e.g.* H_2_O, methanol, amines and hydrazine) under mild conditions, accessing corresponding acids, esters and amides in high yields. For example, 1-ethyl-3-methylimidazolium glycolate ([EMIm][Gac]) could achieve PGA decomposition into various chemicals such as glycolic acid, alkyl glycolates, 2-hydroxy amides, 2-(hydroxymethyl)benzimidazole, and 1,3-benzothiazol-2-ylmethanol in excellent yields. In addition, the IL catalyst could be easily recovered and reused without obvious activity loss. This protocol opens a simple and efficient way to decompose biopolyesters into valuable chemicals, which may have promising applications.

## Experimental

### General procedures for preparation of hydroxyl carboxylate anion-based ILs

[EMIm][Gac], [EMIm][Lac] and [EMIm][Hb] were synthesized by neutralizing the corresponding base [EMIm][OH] and acid (glycolic acid, lactic acid and 3-hydroxybutyric acid), respectively. In a typical experiment, an ethanol solution of [EMIm][OH] was obtained from [EMIm][Cl] using the anion-exchange resin method, and then neutralized with an equimolar amount of glycolic acid. After removing ethanol and water by evaporation under reduced pressure, the obtained [EMIm][Gac] was thoroughly washed with diethyl ether, and finally dried under vacuum at 70 °C for 72 h.

### General procedure for depolymerization of polyesters

In a typical experiment, PGA (58.0 mg) and [EMIm][Gac] (2 mmol) were loaded into a 10 mL flask equipped with a magnetic stirrer in a glovebox under an N_2_ atmosphere, and then the flask was sealed. The reaction mixture was stirred at 110 °C for 15 h. After the reaction, the flask was cooled down to room temperature. The resultant liquid mixture was analyzed by ^1^H NMR and ^13^C NMR in DMSO-d_6_.

### General procedure for one-pot decomposition of PGA with 2-aminothiophenol in [EMIm][Gac]

In a typical experiment, [EMIm][Gac] (2 mmol), PGA (58 mg) and 2-aminothiophenol (1.5 mmol) were sequentially loaded into a 10 mL flask equipped with a magnetic stirrer and sealed under a nitrogen atmosphere. The reaction mixture was stirred at the desired temperature (*e.g.*, 110 °C) for 12 h. After the reaction, the reactor was cooled down in ice water. The quantitative analysis was conducted by ^1^H NMR analysis using ultra-dry dimethylformamide as an internal standard.

## Results and discussion

**Fig. 1:**
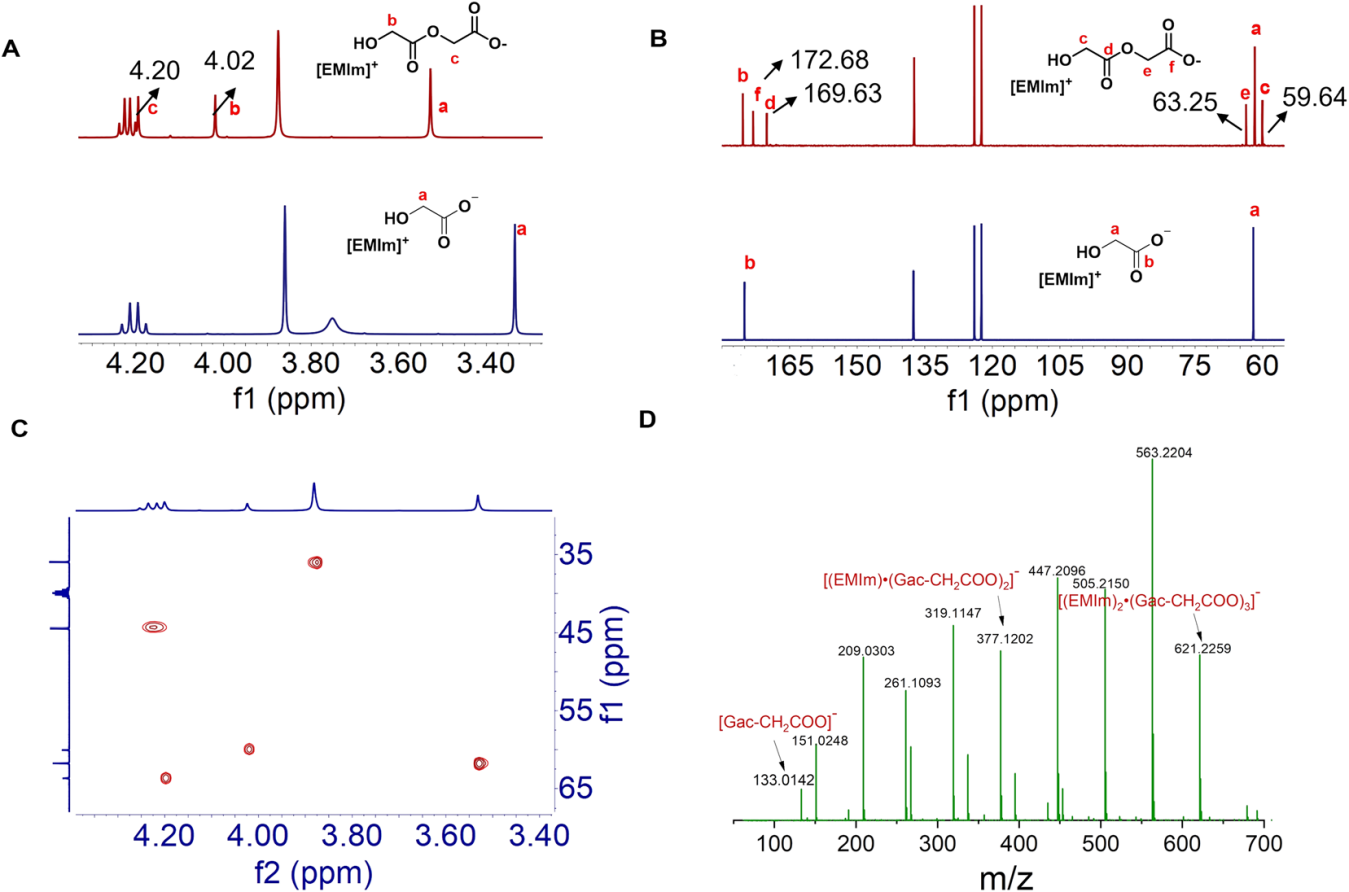
NMR and HR-ESI-MS spectra of the reaction solution. Reaction conditions: PGA (58 mg), [EMIm][Gac] (2 mmol), 110 °C, 15 h. (A) ^1^H NMR spectra; (B) ^13^C NMR spectra; (C) HSQC spectrum; (D) magnification of the selected region of the ESI-MS(−) spectrum.

Initially, PGA was selected as an example of biopolyesters to screen the IL catalysts. The PGA with a desired amount was exposed to different ILs at 110 °C for 15 h. The PGA particles almost kept unchanged in [EMIm][Cl], [EMIm][Br], [EMIm][BF_4_], [EMIm][NTf_2_], [EMIm][OTs], [EMIm][OTf] and [HOEMIm][OTf], suggesting that these ILs could hardly dissolve this polymer, whereas in the ILs with the glycolate anion ([Gac]^−^), *e.g.*, [EMIm][Gac], [BMIm][Gac], [HMIm][Gac], [TBP][Gac] and [HDBU][Gac], they became progressively smaller until a solution was formed. To get detailed information, the solution of [EMIm][Gac] and PGA was examined by NMR spectroscopy. Obviously, two new peaks appeared at 4.20 and 4.02 ppm in the ^1^H NMR spectrum of the solution (Fig. 1A); meanwhile, four new signals appeared at 59.64, 63.25, 169.63 and 172.68 ppm in the ^13^C NMR spectrum (Fig. 1B). Both ^1^H and ^13^C NMR analyses indicate that PAG was decomposed by [EMIm][Gac] to form new species with specific structures rather than being dissolved in this IL. Considering the chemical structures of the IL and PGA, it is speculated that the transesterification between PGA and the [Gac] anion may occur under the autocatalysis of the [Gac] anion, to form ionic species [Gac–CH_2_COO]^−^, which was verified by heteronuclear singular quantum correlation (HSQC) (Fig. 1C). To further identify the ionic species in the solution, electrospray ionization mass spectrometry (ESI-MS) was performed (Fig. 1D and S1†). Excitingly, [Gac–CH_2_COO]^−^ (*m*/*z* = 133.0142), [(Gac–CH_2_COO)·H_2_O]^−^ (*m*/*z* = 151.0248), [(Gac–CH_2_COO)·(HGac)]^−^ (*m*/*z* = 209.0303), [(EMIm)·(Gac)·(Gac–CH_2_COO)]^−^ (*m*/*z* = 319.1147), [(EMIm)·(Gac–CH_2_COO)_2_]^−^ (*m*/*z* = 377.1202), [(EMIm)_2_·(Gac)·(Gac–CH_2_COO)_2_]^−^ (*m*/*z* = 563.2204), [(EMIm)_2_·(Gac–CH_2_COO)_3_]^−^ (*m*/*z* = 621.2259) and other [Gac–CH_2_COO]^−^-based species were detected in the ESI-MS(−) spectrum, while the aggregates of [Gac–CH_2_COO]^−^-containing ions, such as [(EMIm)_2_·(Gac–CH_2_COO)]^+^ (*m*/*z* = 355.1979) and [(EMIm)_3_·(Gac)·(Gac–CH_2_COO)]^+^ (*m*/*z* = 541.2989), were detected under experimental conditions. The above NMR and ESI-MS analysis results indicate that PAG can be decomposed by [EMIm][Gac] *via* transesterification to form [Gac–CH_2_COO]^−^ species. The other ILs with the [Gac] anion like [BMIm][Gac], [HMIm][Gac] and [HDBU][Gac] could also decompose PGA into [Gac–CH_2_COO]^−^ species, confirmed by NMR spectroscopy analysis (Fig. S2†). The above results clearly indicate that the [Gac] anion of these ILs can react with PGA to produce [Gac]-based anion species stabilized by the IL cations.

Inspired by the above results, we examined other ILs with the lactate anion ([Lac]^−^, *e.g.* [EMIm][Lac]) and with the 2-hydroxyl butylate anion ([Hb]^−^, *e.g.*, [EMIm][Hb]) to depolymerize PLA and PHB at 110 °C, respectively. As expected, [EMIm][Lac] could decompose PLA into [Lac–CH(CH_3_)–COO]^−^ species (Fig. S3†), confirmed by NMR analysis, and [EMIm][Hb] could decompose PHB into [Hb–CH(CH_3_)–CH_2_COO]^−^ species (Fig. S4†). Notably, the [Hb–CH(CH_3_)–CH_2_COO]^−^ species could be readily dehydrated to form crotonic acid under the reaction temperature, and crotonic acid was the final product upon prolonging the reaction time to 12 h (Fig. S4B and C†), which is identical to the reported results that the acetate anion could catalyze PHB depolymerization to crotonic acid.^28^ From the above results, it can be deduced that hydroxyl carboxylate anions are reactive to decompose polyesters *via* transesterification to form hydroxyl carboxylate anion-based species.

To detect the reactivity of the ionic species ([Gac–CH_2_COO]^−^) derived from PGA decomposition with [Gac] anion-based ILs, they were employed to react with various nucleophiles including H_2_O, alcohols and amines. It was indicated that as water was added into the [Gac–CH_2_COO]^−^ solutions, glycolic acid was obtained even at 60 °C, suggesting that [Gac–CH_2_COO]^−^ can react with water. As demonstrated, the yields of glycolic acid were impacted by the structures of the IL cations. 1-Alkyl-3-methyl imidazolium glycolate ILs displayed comparable activities affording glycolic acid yields of around 98%, while [TBP][Gac] and [HDBU][Gac] showed much lower activity, with product yields less than 50% (Fig. S5†). These findings indicate that the cations of the ILs impact the activity of the anions, probably due to the interaction between the cation and anion of the ILs. It is reasonable that though [TBP][Gac] and [HDBU][Gac] have the same anion as [EMIm][Gac], their anions show different reactivity towards PGA degradation to generate active [Gac–CH_2_COO]^−^ species due to the influence of the paired cations, thus leading to their diminished activities.

All the tested nucleophiles including water, alcohols and amines could efficiently react with [EMIm][Gac–CH_2_COO] under mild conditions, generating glycolic acid, and corresponding esters and amides in excellent yields at 60 °C in most cases (Scheme S1†). For example, using 2-aminothiophenol (1a) as a nucleophile, 2*H*-1,4-benzothiazin-3-one (2a) and 1,3-benzothiazol-2-ylmethanol (3a) were selectively obtained depending on the reaction temperature (Fig. S6†). From 40 °C to 120 °C, 3a was the main product with the highest yield of about 90% obtained at 100 °C within 6 h. Increasing temperature from 100 to 140 °C, 2a yield was enhanced from about 10% to 55% correspondingly. Similarly, using *o*-phenylenediamine to react with [EMIm][Gac–CH_2_COO], 2-(hydroxymethyl)benzimidazole (3b) was solely obtained in yields >99% in the temperature range of 60–140 °C. The above results indicate that the [Gac–CH_2_COO]^−^ species are very reactive, which can react with various nucleophiles to produce valuable chemicals.

**Scheme 2:**
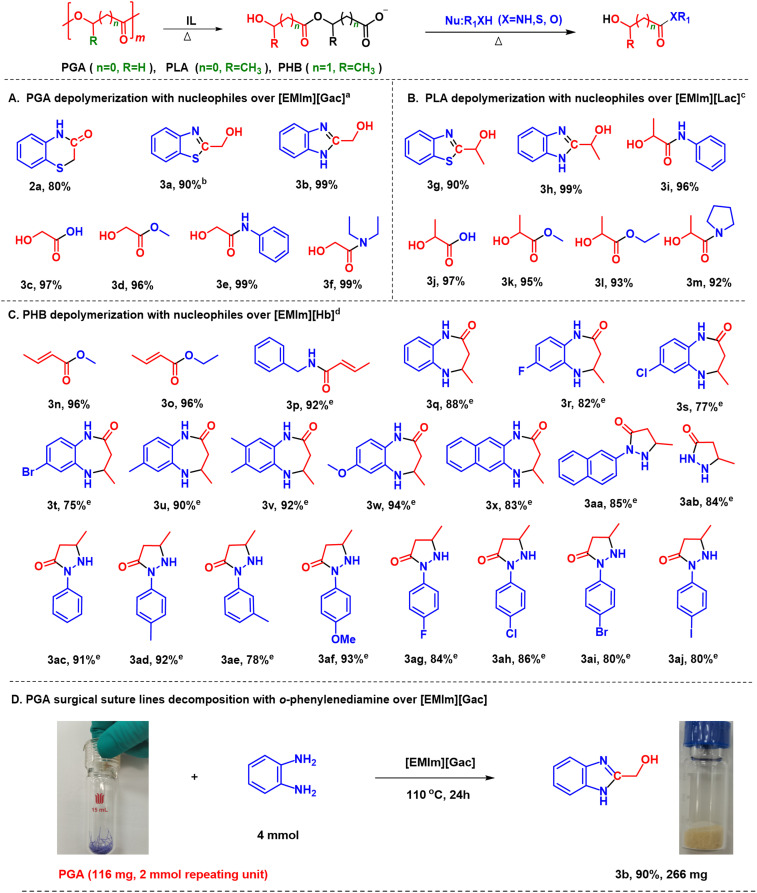
Universality of the designed protocol. (A) PGA depolymerization with nucleophiles; (B) PLA depolymerization with nucleophiles; (C) PHB depolymerization with nucleophiles; (D) PGA surgical suture decomposition with *o*-phenylenediamine. Reaction conditions: ^a^ PGA (58 mg), [EMIm][Gac] (2 mmol), nucleophiles (1.5–3 mmol), 110 °C, 12 h; ^b^1a (1.5 mmol) was added into [EMIm][Gac–CH_2_COO]; ^c^ PLA (72 mg), [EMIm][Lac] (2 mmol), nucleophiles (1.5–3 mmol), 110 °C, 12 h; ^d^ PHB (43 mg), [EMIm][Hb] (0.15 mmol), nucleophiles (1.5–3 mmol), 110 °C, 24 h; ^e^ isolated yields.

Based on the above results, the one-pot decomposition of PGA with different nucleophiles including water, methanol and various amines in [EMIm][Gac] was investigated, respectively, under similar conditions, and corresponding target products were obtained in comparable yields to those achieved from the reactions of [EMIm][Gac–CH_2_COO] with these nucleophiles (Scheme 2A). However, as 2-aminophenol with a similar structure to 2-aminothiophenol was examined for aminolysis of PGA over [EMIm][Gac], no target product was detected under the similar reaction conditions. For comparison, [EMIm][Oac] was employed in the decomposition of PGA with 1a, and 2a was obtained in an inferior yield of <45%. NMR analysis illustrates that this IL can only dissolve PGA, unable to react with PGA to generate an active intermediate (Fig. S7†). From the above results, it can be deduced that [Gac]-catalyzed valorization of PGA undergoes the decomposition of PGA with the [Gac] anion to reactive [Gac–CH_2_COO]^−^ species, and subsequent reaction of [Gac–CH_2_COO]^−^ with nucleophiles. This protocol demonstrates enhanced efficiency and wide substrate scope over traditional depolymerization with a nucleophile, especially under mild conditions.^29–31^

The reusability of [EMIm][Gac] was examined by performing decomposition of PGA with *o*-phenylenediamine. It was demonstrated that the IL kept its activity after being reused for 5 runs, indicating its good stability. In addition, large-scale decomposition of PGA (1.16 g) with *o*-phenylenediamine was performed at 110 °C, and 3.02 g of 3b was obtained, indicating that the decomposition strategy of PGA can be scaled up in practical application.

What is more, to extend this protocol in practical applications, 116 mg of PGA surgical suture lines were collected and tried to be decomposed with *o*-phenylenediamine in [EMIm][Gac]. As expected, this practical PGA feedstock was decomposed, producing 266 mg of 3b, which means that the 3b yield reached 90% (Scheme 2D), demonstrating the promising application prospects.

In this work, the glycolate-based ILs (*e.g.* [EMIm][Gac]) have realized one-pot decomposition of PGA with various nucleophiles, providing a simple and green route to degrade PGA, and accessing a series of glycolic acid-derived valuable chemicals. Inspired by the above results, [EMIm][Lac] as one representative of the lactate-based ILs was examined for decomposition of PLA with various nucleophiles. Interestingly, [EMIm][Lac] exhibited good activity for the degradation of PLA with water, alcohols and amines under similar experimental conditions, affording lactic acid, lactates and lactamides in good to excellent yields (Scheme 2B). In addition, 360 mg of readily available PLA straw was tried to be degraded with *o*-phenylenediamine in [EMIm][Lac], and 723 mg of 3h in an isolated yield of 89% was obtained, implying the additives in the PLA straw had little effect on the decomposition of PLA.

Similarly, the [Hb] anion-based IL, [EMIm][Hb], was employed in decomposition of PHB with various nucleophiles, and a series of crotonic acid-based amides and esters were obtained in excellent yields since the ionic species resulting from PHB and [EMIm][Hb] could be converted into crotonic acid that is a reactive 2,4-biselectrophilic reagent. Especially, various cyclic amides were accessed *via* the decomposition of PHB with *o*-phenylenediamines. In comparison, the *o*-phenylenediamines with electron-donating groups (methyl, dimethyl and methoxyl) afforded the desired products (Scheme 2C, 3u–3w) in excellent yields, showing higher reactivity than those with electron-withdrawing groups (Scheme 2C, 3r–3t). Hydrazines could also decompose PHB, producing a series of pyrazolidines in good to excellent yields (Scheme 2C, 3aa–3aj). In particular, 2-naphthalene hydrazine and hydrazine hydrate generated the corresponding products 3aa and 3ab in high yields of around 85%. In comparison, the phenyl hydrazines with electron-donating groups in the benzene ring at *p*-position (methyl, methoxyl) showed higher reactivity than those with electron-withdrawing substituents (F, Cl, Br and I), affording corresponding products in excellent yields (3ac, 3ad, 3af & 3ag–3aj). *p*-Methylphenyl hydrazine offered the desired product in higher yield than *m*-methylphenyl hydrazine (3ad and 3ae).

Since the above-described ILs, including [EMIm][Gac], [EMIm][Lac], and [EMIm][Hb], can decompose PGA, PLA and PHB, respectively, into corresponding hydroxyl carboxylate anion-based ionic species, they may follow a similar catalytic mechanism to decompose corresponding polyesters. Notably, the anions of the used ILs are derived from monomers of the polyesters, which may efficiently avoid the formation of possible byproducts resulting from the reaction of the IL anion with the polyesters. This is confirmed by the formation of [EMIm][Lac] and [EMIm][Lac–CH(CH_3_)–COO] as poly(lactic-*co*-glycolic acid) (PLGA) was decomposed over [EMIm][Gac] (Fig. S8†).

**Fig. 2:**
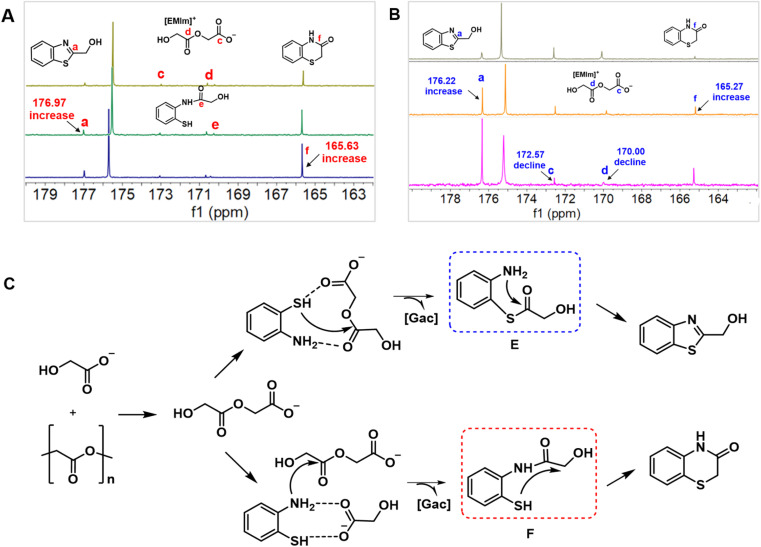
Mechanism study on PGA degradation with 1a. (A) *In situ*^13^C NMR for 2a formation; (B) *in situ*^13^C NMR for 3a formation; (C) proposed reaction pathway.

As described above, the decomposition of PGA with 1a in [EMIm][Gac] could selectively produce 2a and 3a*via* changing the reaction temperature. To explore the reaction mechanism of the control synthesis of 2a and 3a, semi-*in situ*^13^C NMR analysis on the one-pot reaction of PGA with 1a over [EMIm][Gac] was performed. Three new peaks appeared in the range of 173.0 to 169.0 ppm in the ^13^C NMR spectrum of the reaction solution (Fig. 2A). The signals at 173.03 and 170.63 ppm are assigned to the two carbonyl C atoms of [Gac–CH_2_COO]^−^, supported by the results shown in Fig. 1B. The signal at 170.25 ppm may be ascribed to the carbonyl C atom of the intermediate F (as shown in Fig. 2C), formed from nucleophilic attack of the activated N atom in 1a on the carbonyl C of [Gac–CH_2_COO]. Meanwhile, the height of the ^13^C NMR resonance at 165.63 ppm assigned to the carbonyl C atom of 3a, and at 176.97 ppm to the C atom of C=N bond gradually enhanced with reaction time, reflecting degradation of PGA and formation of 2a and 3a. In the case of [EMIm][Gac–CH_2_COO] reacting with 1a, the amount of [EMIm][Gac–CH_2_COO] gradually declined as the reaction proceeded (the peaks appeared at *δ* = 172.57 and 170.00 ppm), accompanied by the production of 2a and 3a (Fig. 2B). However, no intermediate was observed, suggesting that the selective formation of 2a and 3a may undergo a different reaction pathway.

Based on the above experimental results and discussion, a plausible reaction pathway is proposed in Fig. 2C. Initially, the glycolate anion decomposes PGA into ionic intermediate [Gac–CH_2_COO]^−^. Subsequently, the activated S atom in 1a attacks the carbonyl C of [Gac–CH_2_COO]^−^ to form intermediate E, which quickly undergoes intramolecular cyclodehydration to form 3a, thus regenerating the IL (path 1). In the case of forming 2a, it is the activated N atom in 1a that attacks the carbonyl C of [Gac–CH_2_COO] to form intermediate F, and 2a is yielded through the intramolecular cyclization and dehydration (path 2). The reason for temperature determining the reaction pathway may be explained as follows. In comparison, the –NH_2_ group has a basic nature, while the –SH group has an acidic characteristic. At low temperatures, the hydrogen bonding interaction between the NH_2_ group and [Gac] anion enhances the nucleophilicity of the N atom, thus facilitating the preferential occurrence of path 2. At higher temperatures, the hydrogen bonding interaction between the NH_2_ group and [Gac] anion is weakened, while the acidity of the –SH group improves, thus making path 1 more competitive.

## Conclusions

In summary, we present a hydroxyl carboxylate IL-catalyzed protocol for depolymerization and transformation of hydroxyl carboxylic acid-derived polyesters under metal-free and mild conditions, which can accomplish polyester recycling to various chemicals *via* hydrolysis, alcoholysis and aminolysis. In particular, a series of N-heterocyclic amides have been accessed in high yields *via* aminolysis of this kind of polyester. It is verified that the hydroxyl carboxylate anion can readily break down the acyl C–O bond of polyesters, forming reactive ionic species, *i.e.* [Gac–CH_2_COO]^−^. The ionic species serves as a good electrophilic reagent to react with various nucleophiles (*e.g.* H_2_O, methanol, and amines), generating the corresponding monomers of polyesters, esters and amides efficiently. This work opens a novel way to recycle polyesters into valuable chemicals, which may have promising applications.

## Data availability

The data underlying this study are available in the published article and its ESI.†

## Author contributions

Z. L. directed the project and designed the experiments. Y. Z. and F. W. performed experiments and with W. Z. discussed the depolymerization mechanism. H. Z., Y. W., M. T., and R. L. facilitated NMR-related experiments. All authors collaboratively analyzed the data and contributed to writing the manuscript.

## Conflicts of interest

The authors declare no conflict of interest.

## Supplementary Material

SC-015-D4SC02533D-s001
