# Supplementary material for: Hydroxyl carboxylate anion catalyzed depolymerization of biopolyesters and transformation to chemicals
Source: Chem Sci. 2024 Jun 17;15(28):10892–9. doi: 10.1039/d4sc02533d (PMC11253192; doi:10.1039/d4sc02533d)

Electronic Supplementary Material (ESI) for Chemical Science.
This journal is © The Royal Society of Chemistry 2024

Supporting Information

Hydroxyl carboxylate anion catalyzed depolymerization of biopolyesters and transformation to chemicals

Yanfei Zhao,[a,c] Hui Zhang[a,c], Fengtian Wu[a,b]*, Rongxiang Li[a,c], Minhao Tang[a,c], Yusi Wang [a,c], Wei Zeng[a,c], Buxing Han[a,c], Zhimin Liu*[a,c]

[a] Beijing National Laboratory for Molecular Sciences, Key Laboratory of Colloid and Interface and Thermodynamics Department, CAS Research/Education Center for Excellence in Molecular Sciences, Center for Carbon Neutral Chemistry, Institute of Chemistry, Chinese Academy of Sciences, Beijing 100190, China

E-mail: wufengtian123@126.com, liuzm@iccas.ac.cn

[b] Jiangxi Province Key Laboratory of Polymer Micro/Nano Manufacturing and Devices, Jiangxi Province Key Laboratory of Synthetic Chemistry, East China University of Technology, Economic Development Zone, Guanglan Avenue 418, Nanchang 330013, China.

[c] University of Chinese Academy of Sciences, Beijing 100049, China.

Experimental section

Materials

Polyglycolic acid (PGA, granule, Mn ~ 150,000), polylactic acid (PLA, particle, Mw ~ 80,000), poly(β-hydroxybutyrate) (PHB, powder, ≥98%) were purchased from Macklin Biochemical Co., Ltd. The ILs including [EMIm][Cl] (99%), [BMIm][Cl] (99%) and [HMIm][Cl] (99%), were provided by Centre of Green Chemistry and Catalysis, Lanzhou Institute of Chemical Physics (LICP), Chinese Academy of Sciences (CAS). Ion exchange resin (Ambersep|r HPR900 OH), glycolic acid (99%), lactic acid (85%), 3-hydroxybutyric acid (95%), tetrabutylphosphonium bromide (99%), 1,8-diazabicyclo[5.4.0]undec-7-ene（DBU, 98%） were purchased from J&K Scientific Co., Ltd. 2-Aminothiophenol (97%), *o*-phenylenediamine (98%), hydrazine monohydrate (98%) and various substrates of amines, were commercially available from Beijing Innochem Science & Technology CO., Ltd. The other chemicals were purchased from Beijing Chemical Company. All chemicals were of analytical grade and used as received.

General procedure for annulation of 2-aminothiophenol with PGA-derived ionic intermediate

In a typical experiment, [EMIm][Gac-CH_2COO^-] (1 mmol) and 2-aminothiophenol (1.5 mmol) were sequentially loaded into a 10 mL flask and sealed under the nitrogen atmosphere equipped with a magnetic stirrer. The reaction mixture was stirred at the desired temperature (e.g., 60°C) for 24 h. After the reaction, the reactor was cooled down in ice water. The quantitative analysis was conducted by ^{1}H NMR analysis using ultra-dry dimethylformamide yas an internal standard.

General procedures for decomposition of PGA surgical suture lines using *o*-phenylenediamine

In a typical experiment, PGA surgical suture lines (116 mg), *o*-phenylenediamine (4 mmol) and [EMIm][Gac] (4 mmol) were sequentially loaded into a 10 mL flask and sealed under the nitrogen atmosphere equipped with a magnetic stirrer. The reaction mixture was stirred at 110 ^oC for 24 h. After the reaction, the reactor was cooled down in ice water. The reaction mixture was extracted with 3 ml of ethyl acetate for three times, and [EMIm][Gac] was used directly for the next run after removing solvent by rotary evaporation and drying in a vacuum oven at 343 K for 24 h. The combined ethyl acetate solutions were washed with water and brine, followed by treatment with anhydrous Na_2SO_4, and evaporated to remove ethyl acetate. Then, the obtained residue was purified by flash column chromatography on silica gel to yield the desired 3b.

NMR measurements

NMR spectra were recorded on Bruker Avance III 400 HD or 500 WB spectrometer equipped with 5 mm pulsed-field-gradient (PFG) probes. Chemical shifts are given in ppm relative to tetramethylsilane.

For ^{1}H and ^{13}C analysis, pure products were dissolved in DMSO-d_6 and were recorded on Bruker Avance III 400 HD or 500 WB.

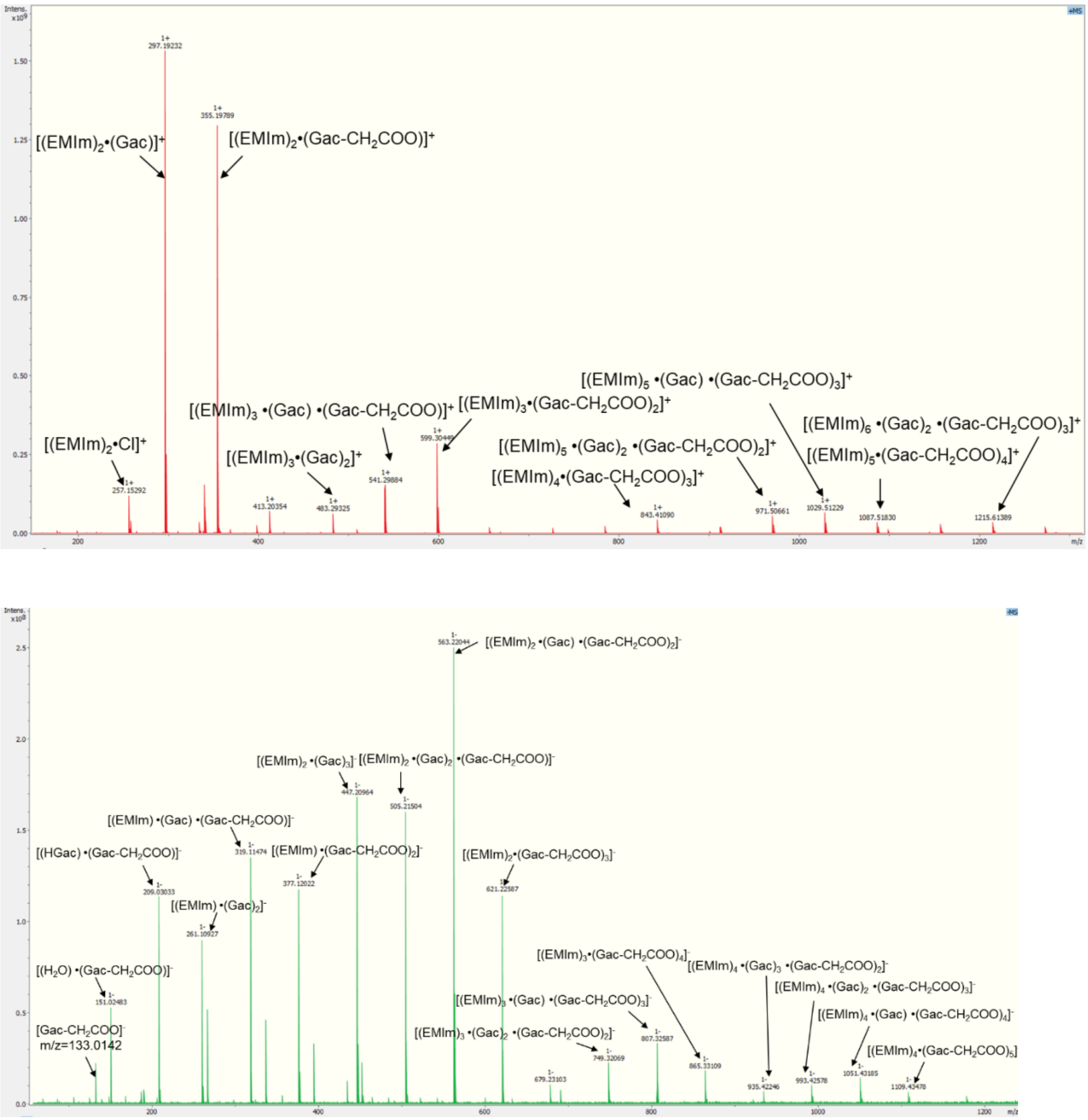

Figure S1. HR-ESI-MS spectra the reaction solution. Reaction conditions: PGA (58 mg), [EMIm][Gac] (2 mmol), 110 ^{o}C, 15h. (a) ESI-MS (+); (b) ESI-MS(-).

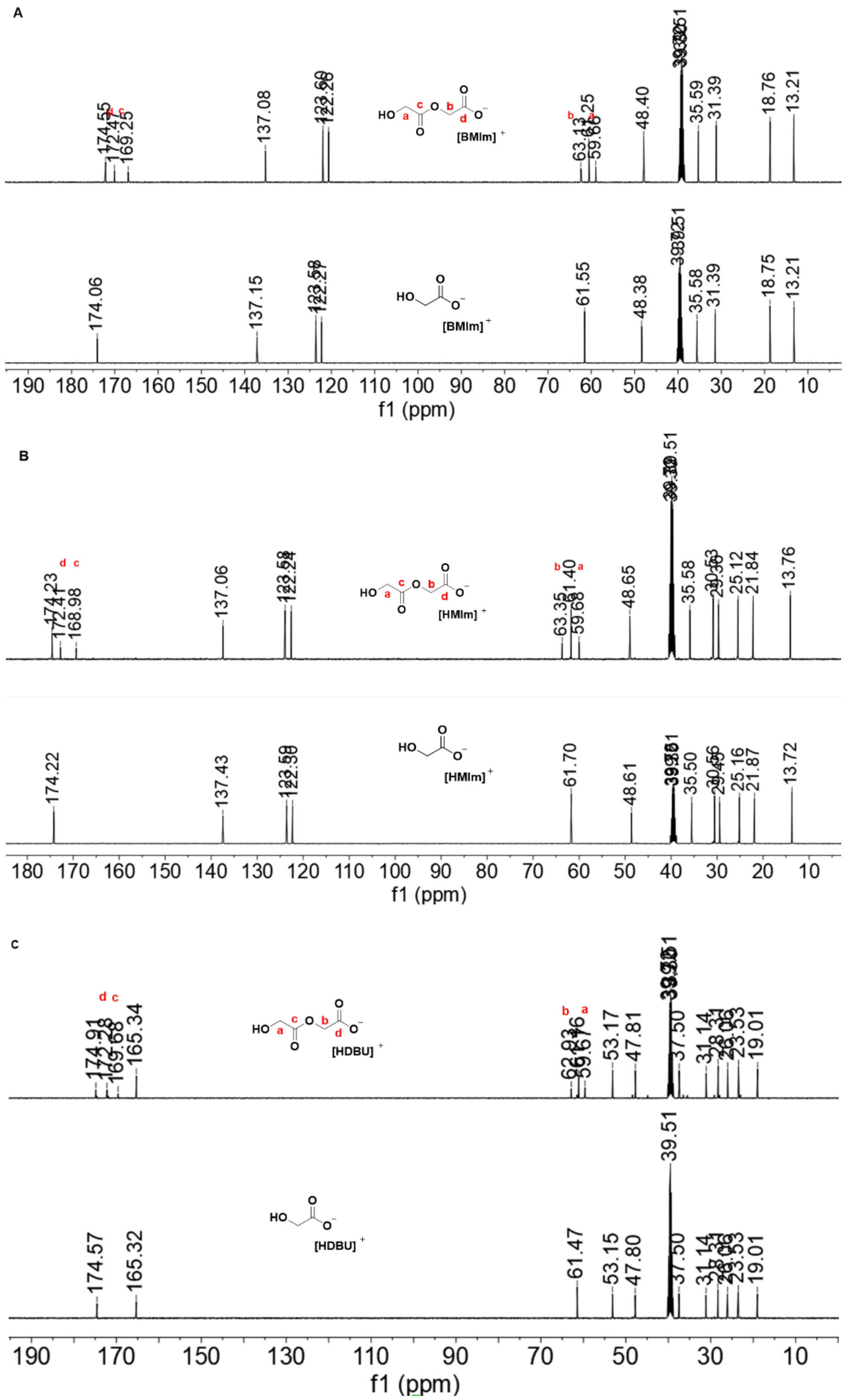

Figure S2 ^{13}C NMR spectra of PGA before and after exposed to (A) [BMIm][Gac], (B) [HMIm][Gac], and (C) [HDBU][Gac] at 110 ^{o}C

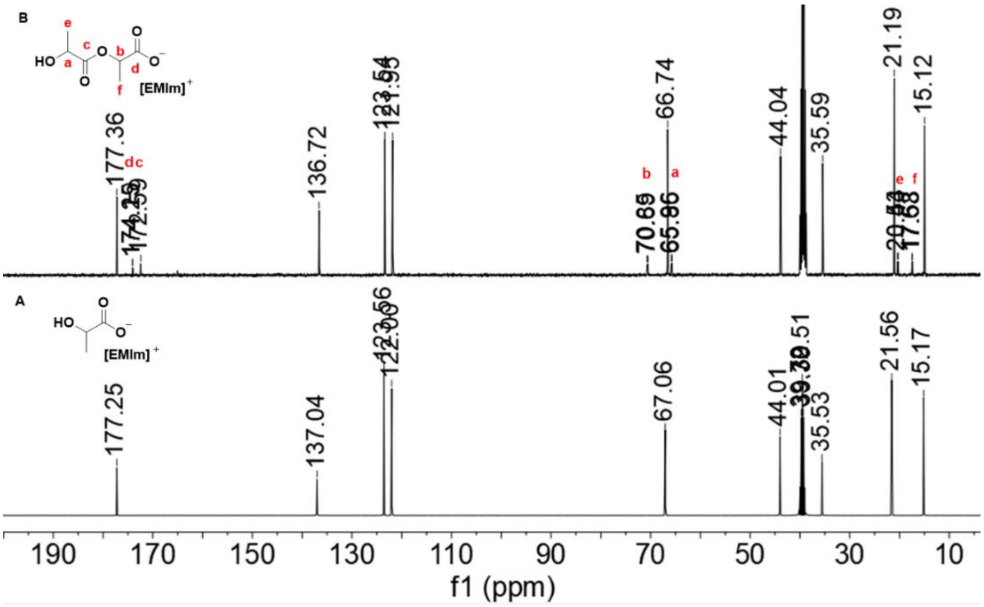

Figure S3 ^{13}C NMR spectra of PLA before (A) and after (B) exposed to [EMIm][Lac] at 110 ^{o}C.

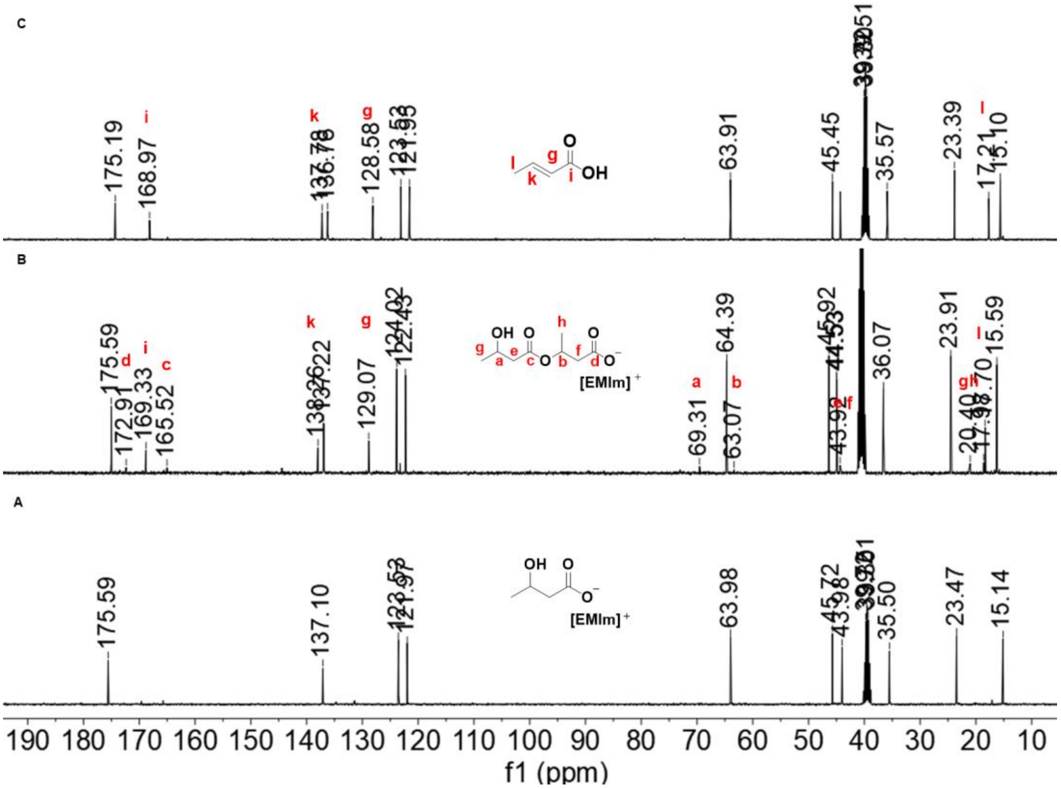

Figure S4 ^{13}C NMR spectra of PHB before (A) and after exposed to [EMIm][Hb] at 110 ^{o}C for 5h (B) and 12 h (C).

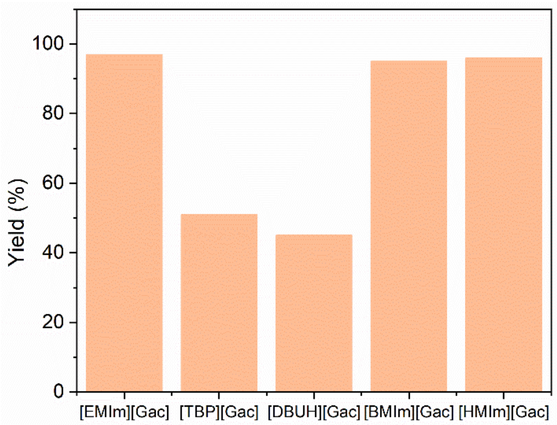

Figure S5. The effect of ionic liquids on the yield of glycolic acid after water was added into the [Gac-$CH_2COO]^-$-based solutions.

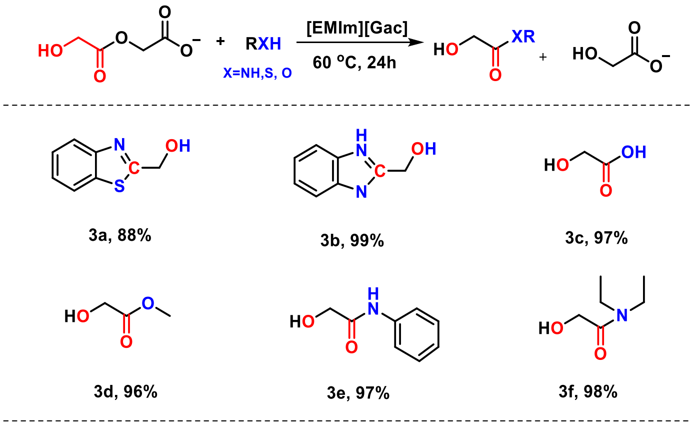

Scheme S1 The reaction of [EMIm][Gac-CH_2COO] with different nucleophiles including water, methanol and amines.

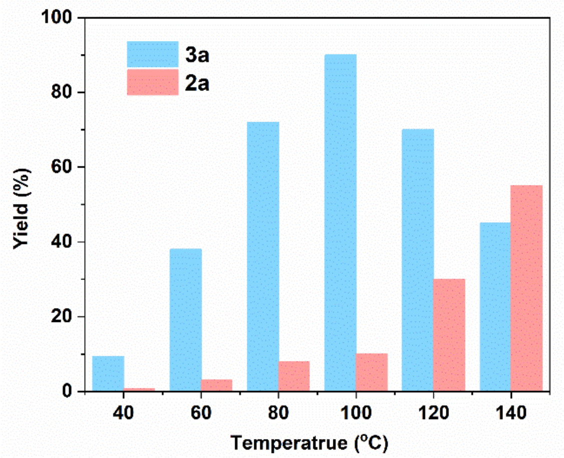

Figure S6. Selective synthesis of 2H-1,4-benzothiazin-3-one (2a) and 1,3-benzothiazol-2-ylmethanol (3a) from [EMIm][Gac-CH_2COO] and 2-aminothiophenol (1a) at different temperature.

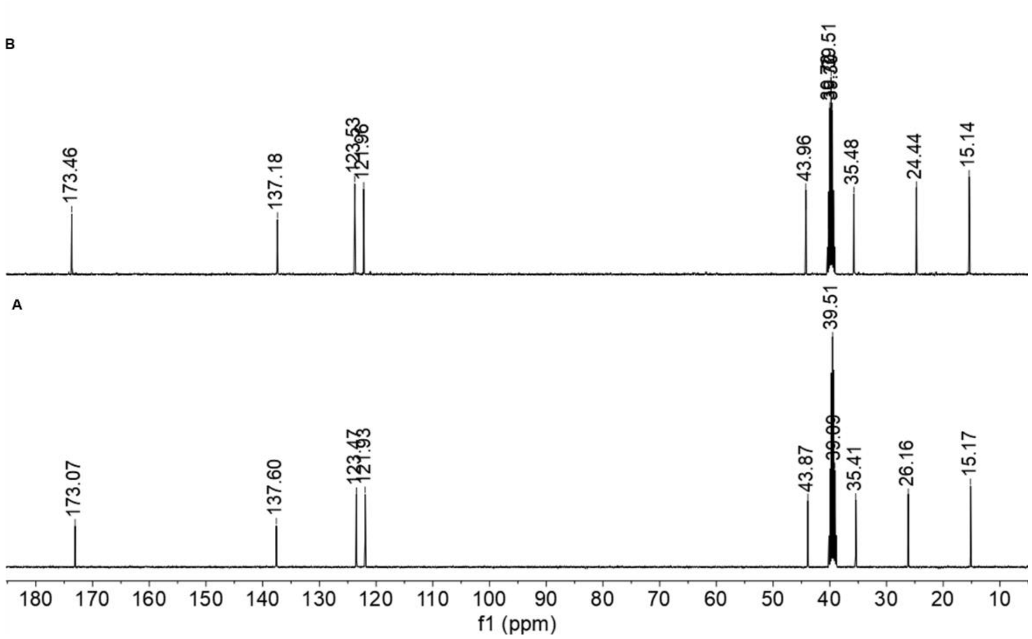

Figure S7 ^{13}C NMR spectra of PGA before(A) and after(B) exposed to [EMIm][Oac] at 110 ^{o}C for 15 h

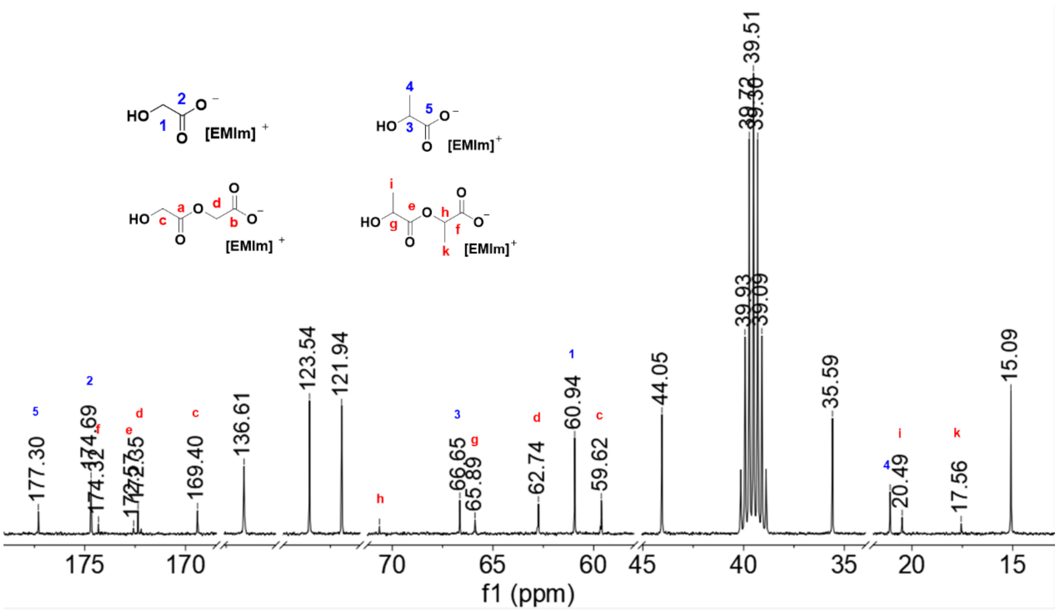

Figure S8 ^{13}C NMR spectra of PLGA after exposed to [EMIm][Gac] at 110 ^{o}C for15 h

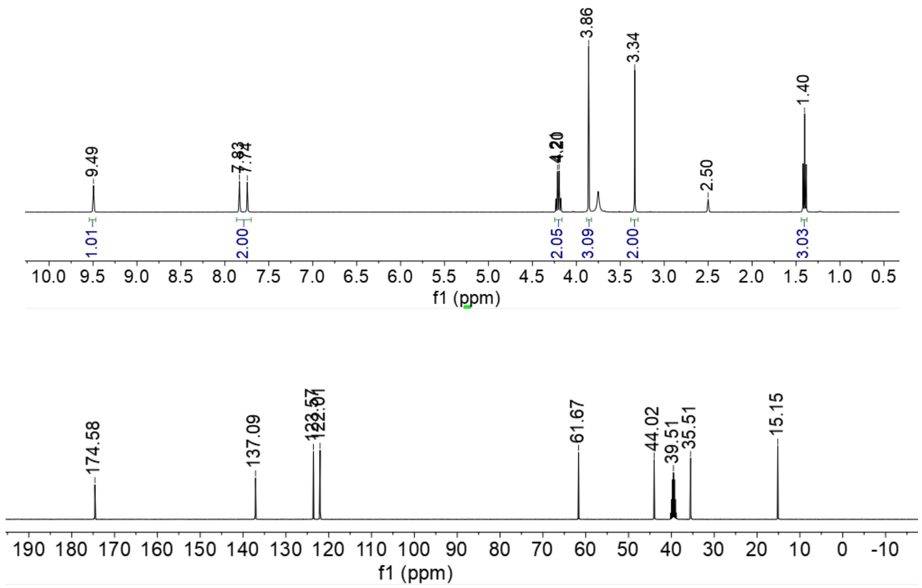

Figure S9 ^{1}H and ^{13}C NMR spectra of [EMIm][Gac]

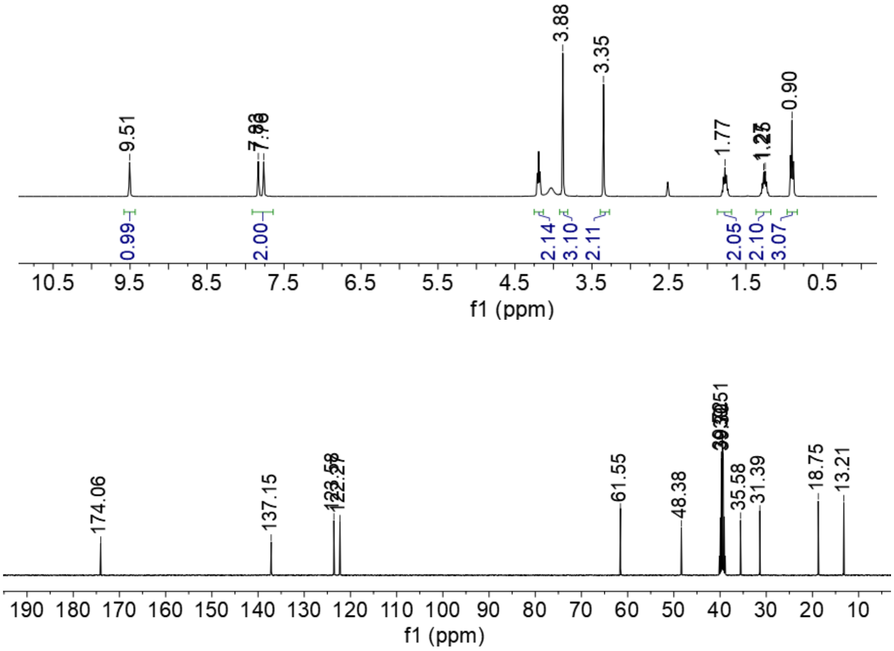

Figure S10 ^{1}H and ^{13}C NMR spectra of [BMIm][Gac]

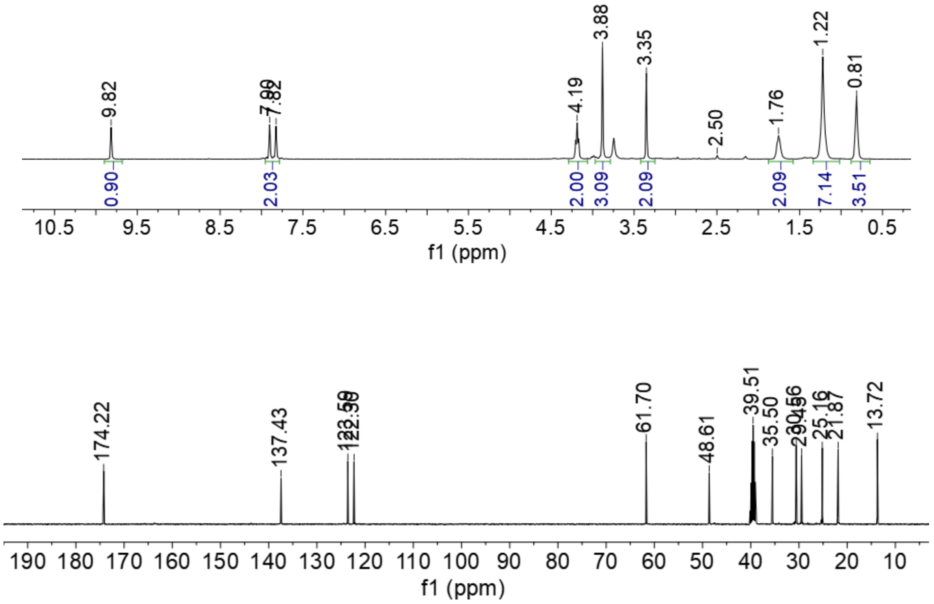

Figure S11 ^{1}H and ^{13}C NMR spectra of [HMIm][Gac]

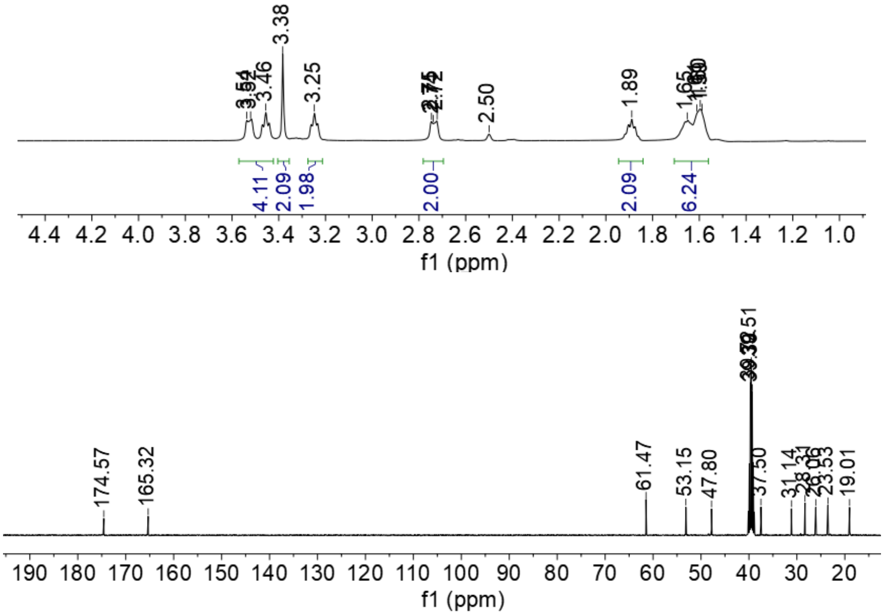

Figure S12 ^{1}H and ^{13}C NMR spectra of [HDBU][Gac]

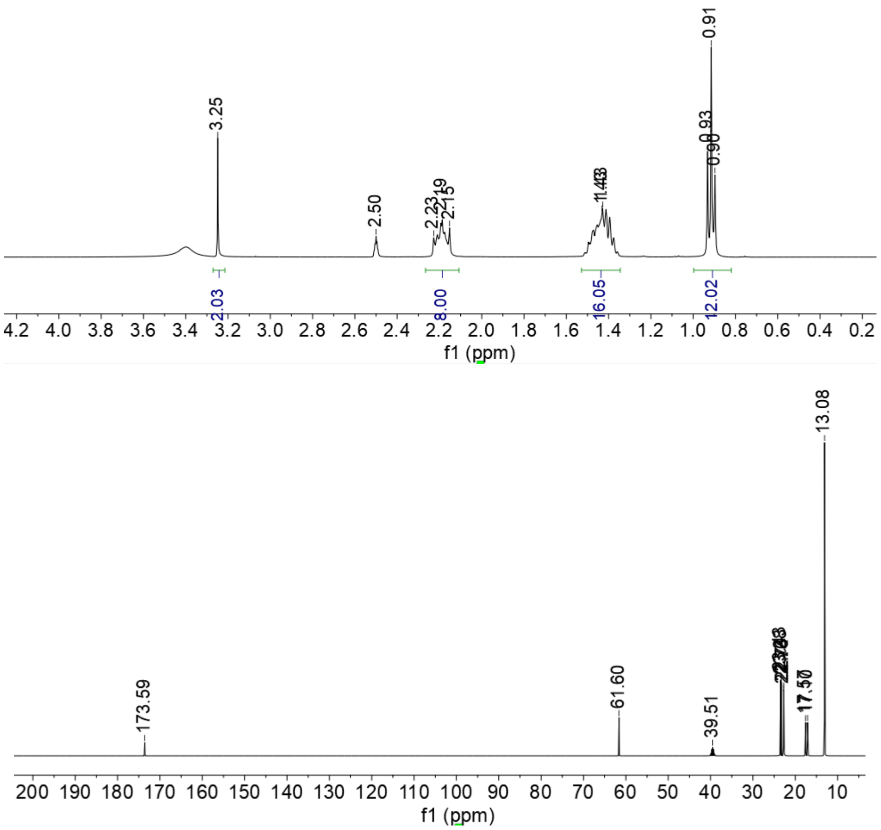

Figure S13 ^{1}H and ^{13}C NMR spectra of [TBP][Gac]

2H-1,4-Benzothiazin-3-one (2a)

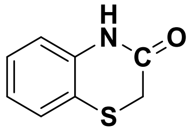

^{1}H NMR (400 MHz, DMSO-d_6, 25 °C): δ = 10.56 (s, 1H), 7.31-7.33 (m, 1H), 7.16-7.20 (m, 1H), 6.96-6.99 (m, 2H), 3.46 ppm (s, 2H); ^{13}C NMR (101 MHz, DMSO-d_6, 25 °C): δ = 165.6, 137.9, 127.7, 127.3, 123.3, 119.5, 117.6, 29.4 ppm.

1,3-Benzothiazol-2-ylmethanol (3a)

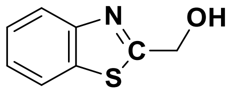

^{1}H NMR (400 MHz, DMSO-d_6, 25 °C): δ = 8.08-8.10 (m, 1H), 7.92-7.94 (m, 1H), 7.48-7.51 (m, 1H), 7.39-7.43 (m, 1H), 6.25 (t, J = 6.0 Hz, 1H), 4.88 ppm (d, J = 6.0 Hz, 2H); ^{13}C NMR (101 MHz, DMSO-d_6, 25 °C): δ =175.6, 153.1, 134.3, 126.0, 124.6, 122.3, 122.2, 61.3 ppm.

2-(Hydroxymethyl)benzimidazole (3b)

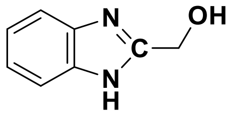

^{1}H NMR (400 MHz, DMSO-d_6, 25 °C): δ = 12.32 (s, 1H), 7.49-7.52 (m, 2H), 7.13-7.15 (m, 2H), 5.71 (t, J = 5.6 Hz, 1H), 4.70 ppm (d, J = 5.6 Hz, 2H); ^{13}C NMR (101 MHz, DMSO-d_6, 25 °C): δ = 155.5, 143.6, 134.7, 122.0, 121.5, 118.9, 111.7, 58.2 ppm.

Glycolic acid (3c)

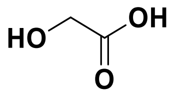

^{1}H NMR (400 MHz, DMSO-d_6, 25 °C): δ = 12.26 (s, 1H), 5.16 (s, 1H), 3.91 ppm (s, 2H); ^{13}C NMR (101 MHz, DMSO-d_6, 25 °C): δ = 174.7, 60.0 ppm.

Glycolic acid methyl ester (3d)

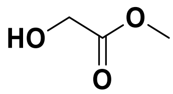

^{1}H NMR (400 MHz, DMSO-d_6, 25 °C): δ = 5.30 (t, J = 6.4 Hz, 1H), 4.01 (d, J = 6.4 Hz, 1H), 3.64 ppm (s, 2H); ^{13}C NMR (101 MHz, DMSO-d_6, 25 °C): δ = 174.7, 60.0 ppm.

Glycolanilide (3e)

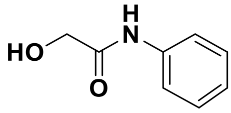

^{1}H NMR (400 MHz, DMSO-d_6, 25 °C): δ = 9.66 (s, 1H), 7.69-7.72 (m, 2H), 7.28-7.33 (m, 2H), 7.03-7.08 (m, 1H), 5.69 (t, J = 7.2 Hz, 1H), 4.00 ppm (d, J = 7.2 Hz, 2H); ^{13}C NMR (101 MHz, DMSO-d_6, 25 °C): δ = 171.3, 139.0, 129.1, 123.9, 120.1, 62.4 ppm.

N, N-Diethyl-2-hydroxyacetamide (3f)

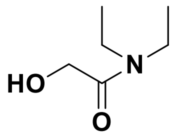

^{1}H NMR (400 MHz, DMSO-d_6, 25 °C): δ = 4.37 (t, J = 7.2 Hz, 1H), 4.05 (d, J = 7.2 Hz, 2H), 3.26-3.33 (m, 2H), 3.16-3.23 (m, 2H), 1.01-1.11 ppm (m, 6H); ^{13}C NMR (101 MHz, DMSO-d_6, 25 °C): δ = 170.9, 60.1, 14.3, 13.3 ppm.

1-(Benzo[d]thiazol-2-yl)ethan-1-ol (3g)

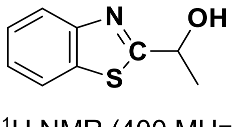

^{1}H NMR (400 MHz, DMSO-d_6, 25 °C): δ = 8.07-8.10 (m, 2H), 7.41-7.49 (m, 2H), 6.34-6.39

(m, 1H), 5.09-5.10 (m, 1H), 1.55-1.60 ppm (m, 3H); ^{13}C NMR (101 MHz, DMSO-d_6, 25 °C): δ = 180.0, 153.7, 134.9, 126.4, 125.1, 122.8, 122.7, 67.6, 24.3 ppm.

1-Benzimidazol-2-ylethanol (3h)

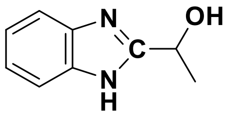

^{1}H NMR (400 MHz, DMSO-d_6, 25 °C): δ = 12.26 (s, 1H), 7.53-7.55 (m, 2H), 7.12-7.14 (m, 2H), 5.80 (d, *J* = 6.4 Hz, 1H), 4.90-4.98 (m, 1H), 1.51 ppm (d, *J* = 8.8 Hz, 3H); ^{13}C NMR (101 MHz, DMSO-d_6, 25 °C): δ = 159.0, 143.6, 134.6, 122.0, 121.3, 118.9, 111.7, 64.2, 23.4 ppm.

Lactanilide (3i)

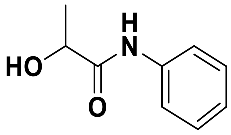

^{1}H NMR (400 MHz, DMSO-d_6, 25 °C): δ = 9.62 (s, 1H), 7.70-7.72 (m, 2H), 7.27-7.33 (m, 2H), 7.03-7.08 (m, 1H), 5.73-5.75 (m, 1H), 4.10-4.19 (m, 1H), 1.31 ppm (d, *J* = 9.2 Hz, 3H); ^{13}C NMR (101 MHz, DMSO-d_6, 25 °C): δ = 174.0, 139.1, 129.1, 123.8, 120.0, 68.2, 21.4 ppm.

Lactic acid (3j)

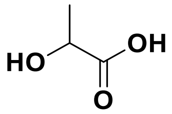

^{1}H NMR (400 MHz, DMSO-d_6, 25 °C): δ = 4.03 (q, *J* = 9.2 Hz, 1H), 1.22 ppm (d, *J* = 9.2 Hz, 3H); ^{13}C NMR (101 MHz, DMSO-d_6, 25 °C): δ = 177.0, 66.2, 20.1 ppm.

Methyl lactate (3k)

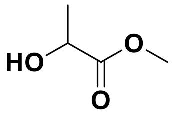

^{1}H NMR (400 MHz, DMSO-d_6, 25 °C): δ = 5.35 (d, *J* = 8.4 Hz, 1H), 4.10-4.19 (m, 1H), 3.63 (s, 3H), 1.24 ppm (d, *J* = 9.2 Hz, 3H); ^{13}C NMR (101 MHz, DMSO-d_6, 25 °C): δ = 175.4, 66.4, 51.7, 20.7 ppm.

Ethyl lactate (3l)

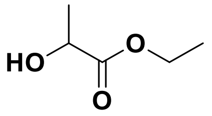

^{1}H NMR (400 MHz, DMSO-d_6, 25 °C): δ = 5.29 (d, *J* = 7.6 Hz, 1H), 4.05-4.15 (m, 3H), 1.17-1.25 ppm (m, 6H); ^{13}C NMR (101 MHz, DMSO-d_6, 25 °C): δ = 175.0, 66.4, 60.3, 20.7, 14.4 ppm.

2-Hydroxy-1-(pyrrolidin-1-yl)propan-1-one (3m)

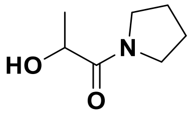

^{1}H NMR (400 MHz, DMSO-d_6, 25 °C): δ = 4.78-4.80 (m, 1H), 4.20-4.29 (m, 1H), 3.51-3.59 (m, 1H), 3.22-3.41 (m, 3H), 1.71-1.91 (m, 4H), 1.16 ppm (d, J = 7.6 Hz, 3H); ^{13}C NMR (101 MHz, DMSO-d_6, 25 °C): δ = 172.6, 65.7, 46.1, 45.9, 26.2, 23.9, 20.5 ppm.

Methyl crotonate (3n)

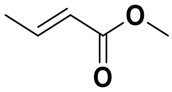

^{1}H NMR (400 MHz, DMSO-d_6, 25 °C): δ = 6.86-6.95 (m, 1H), 5.88-5.92 (m, 1H), 3.64 (s, 3H), 1.85 ppm (d, J = 6.8 Hz, 3H); ^{13}C NMR (101 MHz, DMSO-d_6, 25 °C): δ = 166.5, 145.6, 122.4, 51.5, 18.1 ppm.

Ethyl crotonate (3o)

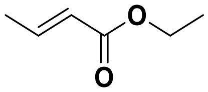

^{1}H NMR (400 MHz, DMSO-d_6, 25 °C): δ = 6.85-6.94 (m, 1H), 5.85-5.90 (m, 1H), 4.08-4.13 (m, 2H), 1.84-1.86 (m, 3H), 1.19-1.22 ppm (m, 3H); ^{13}C NMR (101 MHz, DMSO-d_6, 25 °C): δ = 166.0, 145.3, 122.8, 60.0, 18.0, 14.5 ppm.

(2E)-N-Benzylbut-2-enamide (3p)

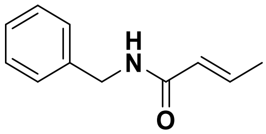

^{1}H NMR (400 MHz, DMSO-d_6, 25 °C): δ = 8.41 (s, 1H), 7.22-7.35 (m, 5H), 6.61-6.73 (m, 1H), 5.95-6.01 (m, 1H), 4.33 (d, J = 8.0 Hz, 2H), 1.79-1.82 ppm (m, 3H); ^{13}C NMR (101 MHz, DMSO-d_6, 25 °C): δ = 165.3, 140.1, 138.5, 128.8, 127.7, 127.2, 126.2, 42.5, 17.8 ppm.

4-Methyl-1,3,4,5-tetrahydro-2H-1,5-benzodiazepin-2-one (3q)

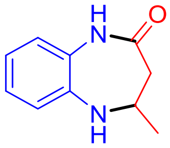

^{1}H NMR (400 MHz, DMSO-d6, 25 °C): δ = 9.43 (s, 1H), 6.83-6.87 (m, 3H), 6.68-6.73 (m, 1H), 5.27 (s, 1H), 3.82-3.84 (m, 1H), 2.41-2.47 (m, 1H), 2.17-2.41 (m, 1H), 1.18 ppm (d, J = 8.8 Hz, 3H); ^{13}C NMR (101 MHz, DMSO-d_6, 25 °C): δ = 171.9, 139.8, 128.5, 124.9, 122.2, 120.7, 119.8, 53.5, 42.1, 23.7 ppm.

7-Fluoro-4-methyl-1,3,4,5-tetrahydro-2H-benzo[*b*][1,4]diazepin-2-one (3r)

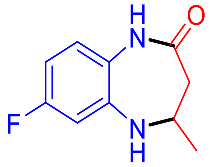

^{1}H NMR (400 MHz, DMSO-d6, 25 °C): δ = 9.55 (s, 1H), 9.43 (s, 0.5H), 6.60-6.89 (m, 5H), 6.45-6.52 (m, 1H), 5.65 (s, 0.6H), 5.17 (s, 1H), 3.80-3.84 (m, 2H), 2.42-2.47 (m, 1.5 H), 2.14-2.29 (m, 2H), 1.16-1.20 ppm (m, 5H); ^{13}C NMR (101 MHz, DMSO-d_6, 25 °C): δ = 172.2, 136.4, 130.6, 121.9 (J_{C-F} = 12.0 Hz), 111.2, 110.9, 108.5, 108.2, 54.3, 41.8, 23.4 ppm; ^{19}F NMR (400 MHz, DMSO-d6, 25 °C): δ = -119.3 ppm.

7-Chloro-4-methyl-1,3,4,5-tetrahydro-2*H*-benzo[*b*][1,4]diazepin-2-one (3s)

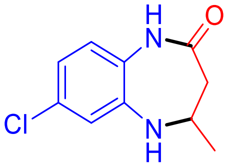

^{1}H NMR (400 MHz, DMSO-d6, 25 °C): δ = 9.59 (d, J = 9.2 Hz, 1H), 6.87-6.98 (m, 5H), 6.74-6.77 (m, 1H), 5.71 (s, 1 H), 5.58 (s, 1H), 3.84-3.90 (m, 2H), 2.50-2.51 (m, 1 H), 2.27-2.36 (m, 2H), 1.23-1.29 ppm (m, 6H); ^{13}C NMR (101 MHz, DMSO-d_6, 25 °C): δ = 171.9, 171.7, 141.1, 138.8, 129.2, 128.3, 126.5, 124.3, 123.5, 122.6, 121.6, 121.3, 119.1, 118.6, 52.9, 52.6, 42.4, 23.7, 23.6 ppm.

7-Bromo -4-methyl-1,3,4,5-tetrahydro-2*H*-benzo[*b*][1,4]diazepin-2-one (3t)

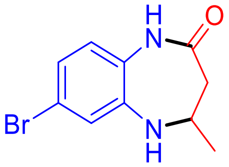

^{1}H NMR (400 MHz, DMSO-d6, 25 °C): δ = 9.55 (s, 1H), 9.44 (s, 0.6H), 6.82-6.89 (m, 2H), 6.60-6.76 (m, 3H), 6.45-6.52 (m, 1H), 5.65 (s, 0.8H), 5.16 (s, 1H), 3.80-3.84 (m, 2H), 2.42-2.48 (m, 2 H), 2.14-2.29 (m, 2H), 1.16-1.24 ppm (m, 6H); ^{13}C NMR (101 MHz, DMSO-d_6, 25 °C): δ = 172.2, 171.7, 141.5, 136.4, 130.7, 123.9, 123.5, 121.9, 111.2, 110.9, 108.5, 108.2, 105.7, 100.0, 54.3, 52.8, 42.1, 41.9, 23.8, 23.4 ppm.

4,7-dimethyl-1,3,4,5-tetrahydro-benzo[b][1,4]diazepin-2-one (3u)

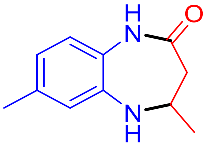

^{1}H NMR (400 MHz, DMSO-d6, 25 °C): δ = 9.43 (s, 1H), 9.39 (s, 0.8H), 6.71-6.83 (m, 5H), 6.57-6.60 (m, 0.8H), 5.22 (s, 0.8H), 5.07 (s, 1H), 3.85-3.88 (m, 2H), 2.43-2.49 (m, 2H), 2.22 (s, 6H), 1.21-1.24 ppm (m, 6H); ^{13}C NMR (101 MHz, DMSO-d_6, 25 °C): δ = 172.1,

171.9, 139.6, 137.4, 133.8, 129.3, 129.1, 126.1, 125.5, 122.4, 122.1, 121.1, 121.0, 120.5, 54.4, 53.6, 42.0, 41.8, 23.8, 23.6, 20.9, 20.6 ppm.

4,7,8-Trimethyl-1,3,4,5-tetrahydro-2*H*-benzo[b][1,4]diazepin-2-one (3v)

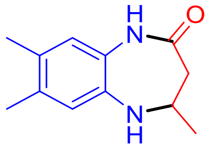

^{1}H NMR (400 MHz, DMSO-d6, 25 °C): δ = 9.32 (s, 1H), 6.68 (d, J = 8.8 Hz, 2H), 4.96 (s, 1H), 3.84-3.86 (m, 1H), 2.41-2.45 (m, 2 H), 2.17-2.20 (m, 1H), 2.12 (d, J = 4.8 Hz, 6H), 1.21 ppm (d, J = 30.0 Hz, 3H); ^{13}C NMR (101 MHz, DMSO-d_6, 25 °C): δ = 172.0, 137.5, 132.4, 127.6, 126.9, 123.1, 122.2, 54.3, 41.8, 23.7, 19.3, 18.9 ppm.

7-Methoxy-4-methyl-1,3,4,5-tetrahydro-2*H*-benzo[*b*][1,4]diazepin-2-one (3w)

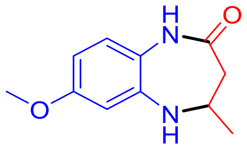

^{1}H NMR (400 MHz, DMSO-d6, 25 °C): δ = 9.44 (s, 1H), 9.30 (s, 0.5H), 6.87-6.89 (m, 1H), 6.80-6.82 (m, 0.8H), 6.59-6.62 (m, 0.8H), 6.49-6.54 (m, 1.6H), 6.35-6.38 (s, 0.7H), 5.35 (s, 0.8H), 4.82 (s, 1H), 3.84-3.87 (m, 2H), 3.72 (s, 4.5H), 2.43-2.48 (m, 1.8H), 2.22-2.28 (m, 0.8 H), 2.09-2.17 (m, 0.8H), 1.19-1.24 ppm (m, 5H); ^{13}C NMR (101 MHz, DMSO-d_6, 25 °C): δ = 172.3, 171.7, 156.9, 154.1, 141.0, 133.5, 131.6, 123.1, 122.3, 121.7, 110.3, 107.7, 105.4, 105.3, 55.7, 55.4, 55.3, 53.4, 41.9, 41.6, 23.8, 23.4 ppm.

4-Methyl-1,3,4,5-tetrahydro-2*H*-naphtho[2,3-*b*][1,4]diazepin-2-one (3x)

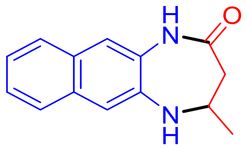

^{1}H NMR (400 MHz, DMSO-d6, 25 °C): δ = 9.84 (s, 1H), 7.66-7.73 (m, 2H), 7.27-7.40 (m, 4H), 5.55-5.56 (m, 1H), 3.92-3.94 (m, 1H), 2.52-2.55 (m, 1H), 2.21-2.28 (m, 1H), 1.27 ppm (d, J = 8.4 Hz, 3H); ^{13}C NMR (101 MHz, DMSO-d_6, 25 °C): δ = 171.9, 140.0, 132.2, 131.9, 129.0, 127.1, 125.4, 123.9, 118.6, 116.0, 54.1, 41.0, 23.4 ppm.

5-Methyl-2-(naphthalen-2-yl)pyrazolidin-3-one (3aa)

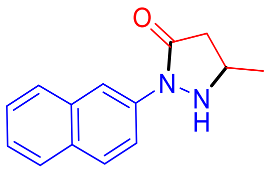

^{1}H NMR (400 MHz, DMSO-d6, 25 °C): δ = 8.15-8.17 (m, 2H), 7.85-7.91 (m, 3H), 7.40-7.51 (m, 2H), 6.26 (d, J = 12.4 Hz, 1H), 3.64-3.72 (m, 1H), 2.76-2.84 (m, 1H), 2,39-2.47 (m, 1H), 1.25 ppm (d, J = 8.8 Hz, 3H); ^{13}C NMR (101 MHz, DMSO-d_6, 25 °C): δ = 173.0, 137.6,

133.6, 130.1, 128.6, 127.9, 126.9, 125.2, 118.5, 114.5, 56.5, 42.6, 19.0 ppm.

5-Methylpyrazolidin-3-one (3ab)

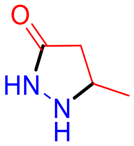

^{1}H NMR (400 MHz, DMSO-d6, 25 °C): δ = 8.95 (s, 1H), 5.00 (s, 1H), 3.45-3.57 (m, 1H), 2.29-2.36 (m, 1H), 1.90-1.98 (m, 1H), 1.09 ppm (d, J = 8.8 Hz, 3H); ^{13}C NMR (101 MHz, DMSO-d_6, 25 °C): δ = 176.8, 53.9, 18.8 ppm.

5-Methyl-2-phenylpyrazolidin-3-one (3ac)

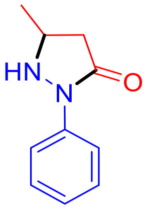

^{1}H NMR (400 MHz, DMSO-d6, 25 °C): δ = 7.77-7.80 (m, 2H), 7.31-7.36 (m, 2H), 7.04-7.09 (m, 1H), 6.10 (d, J = 8.4 Hz, 1H), 3.55-3.69 (m, 1H), 2.69-2.77 (m, 1H), 2.32-2.40 (m, 1H), 1.19 ppm (d, J = 8.4 Hz, 3H); ^{13}C NMR (101 MHz, DMSO-d_6, 25 °C): δ = 172.7, 139.9, 128.9, 123.7, 118.1, 50.5, 42.5, 18.5 ppm.

5-Methyl-2-(*p*-tolyl)pyrazolidin-3-one (3ad)

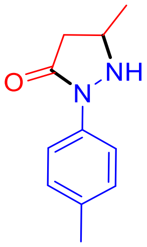

^{1}H NMR (400 MHz, DMSO-d6, 25 °C): δ = 7.65-7.67 (m, 2H), 7.13-7.15 (m, 2H), 6.07 (d, J = 12.0 Hz, 1H), 3.55-3.65 (m, 1H), 2.67-2.74 (m, 1H), 2.29-2.37 (m, 1H), 2.26 (s, 3H), 1.18 ppm (d, J = 8.8 Hz, 3H); ^{13}C NMR (101 MHz, DMSO-d_6, 25 °C): δ = 172.3, 137.5, 132.7, 129.3, 118.2, 50.5, 42.6, 20.9, 18.5 ppm.

5-Methyl-2-(*m*-tolyl)pyrazolidin-3-one (3ae)

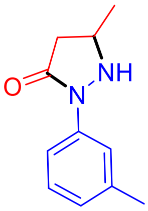

^{1}H NMR (400 MHz, DMSO-d6, 25 °C): δ = 7.60-7.62 (m, 2H), 7.19-7.24 (m, 1H), 6.87-6.90 (m, 1H), 6.07 (d, J = 12.0 Hz, 1H), 3.55-3.65 (m, 1H), 2.68-2.76 (m, 1H), 2.3-2.38 (m, 1H),

2.29 (s, 3H), 1.19 ppm (d, J = 8.8 Hz, 3H); ^{13}C NMR (101 MHz, DMSO-d_6, 25 °C): δ = 172.6, 139.9, 138.1, 128.8, 124.4, 118.6, 115.4, 50.5, 42.5, 21.7, 18.5 ppm.

2-(4-Methoxyphenyl)-5-methylpyrazolidin-3-one (3af)

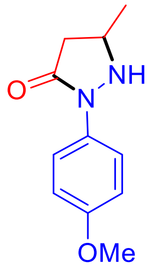

^{1}H NMR (400 MHz, DMSO-d6, 25 °C): δ = 7.67-7.70 (m, 2H), 6.90-6.93 (m, 2H), 6.07 (d, J = 12.4 Hz, 1H), 3.73 (s, 3H), 3.56-3.66 (m, 1H), 2.66-2.74 (m, 1H), 2.28-2.36 (m, 1H), 1.19 ppm (d, J = 8.4 Hz, 3H); ^{13}C NMR (101 MHz, DMSO-d_6, 25 °C): δ = 171.8, 155.7, 133.4, 119.8, 114.1, 55.7, 50.6, 42.3, 18.6 ppm.

2-(4-Fluorophenyl)-5-methylpyrazolidin-3-one (3ag)

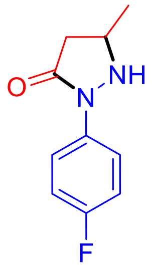

^{1}H NMR (400 MHz, DMSO-d6, 25 °C): δ = 7.81-7.88 (m, 2H), 7.19-7.28 (m, 2H), 6.20 (d, J = 12.0 Hz, 1H), 3.60-3.75 (m, 1H), 2.75-2.82 (m, 1H), 2.37-2.45 (m, 1H), 1.24 ppm (d, J = 8.8 Hz, 3H); ^{13}C NMR (101 MHz, DMSO-d_6, 25 °C): δ = 172.5, 160.0, 156.8, 136.3 ($J_{C\text{-}F}$ = 3.0 Hz), 139.9 ($J_{C\text{-}F}$ = 10.0 Hz), 115.7, 115.4, 50.6, 42.3, 18.5 ppm; ^{19}F NMR (400 MHz, DMSO-d6, 25 °C): δ = -119.4 ppm.

2-(4-Chlorophenyl)-5-methylpyrazolidin-3-one (3ah)

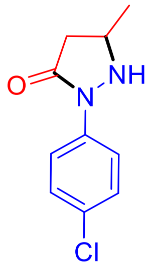

^{1}H NMR (400 MHz, DMSO-d6, 25 °C): δ = 7.79-7.82 (m, 2H), 7.38-7.41 (m, 2H), 6.15 (d, J = 12.0 Hz, 1H), 3.55-3.70 (m, 1H), 2.70-2.78 (m, 1H), 2.33-2.41 (m, 1H), 1.19 ppm (d, J = 8.8 Hz, 3H); ^{13}C NMR (101 MHz, DMSO-d_6, 25 °C): δ = 173.0, 138.7, 128.9, 127.3, 119.6, 50.6, 42.3, 18.4 ppm.

2-(4-Bromophenyl)-5-methylpyrazolidin-3-one (3ai)

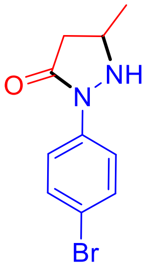

^{1}H NMR (400 MHz, DMSO-d6, 25 °C): δ = 7.74-7.77 (m, 2H), 7.52-7.54 (m, 2H), 6.15 (d, J = 12.0 Hz, 1H), 3.60-3.65 (m, 1H), 2.70-2.76 (m, 1H), 2.33-2.41 (m, 1H), 1.19 ppm (d, J = 8.8 Hz, 3H); ^{13}C NMR (101 MHz, DMSO-d_6, 25 °C): δ = 173.0, 139.1, 131.8, 119.9, 115.4, 50.6, 42.4, 18.4 ppm.

2-(4-Iodophenyl)-5-methylpyrazolidin-3-one (3aj)

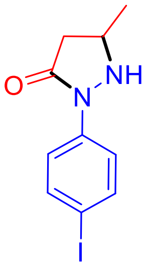

^{1}H NMR (400 MHz, DMSO-d6, 25 °C): δ = 7.60-7.70 (m, 4H), 6.12 (d, J = 12.0 Hz, 1H), 3.57-3.67 (m, 1H), 2.69-2.77 (m, 1H), 2.32-2.40 (m, 1H), 1.19 ppm (d, J = 8.8 Hz, 3H); ^{13}C NMR (101 MHz, DMSO-d_6, 25 °C): δ = 173.0, 139.6, 137.6, 120.2, 87.3, 50.6, 42.4, 18.4 ppm.

2H-1,4-Benzothiazin-3-one (2a)

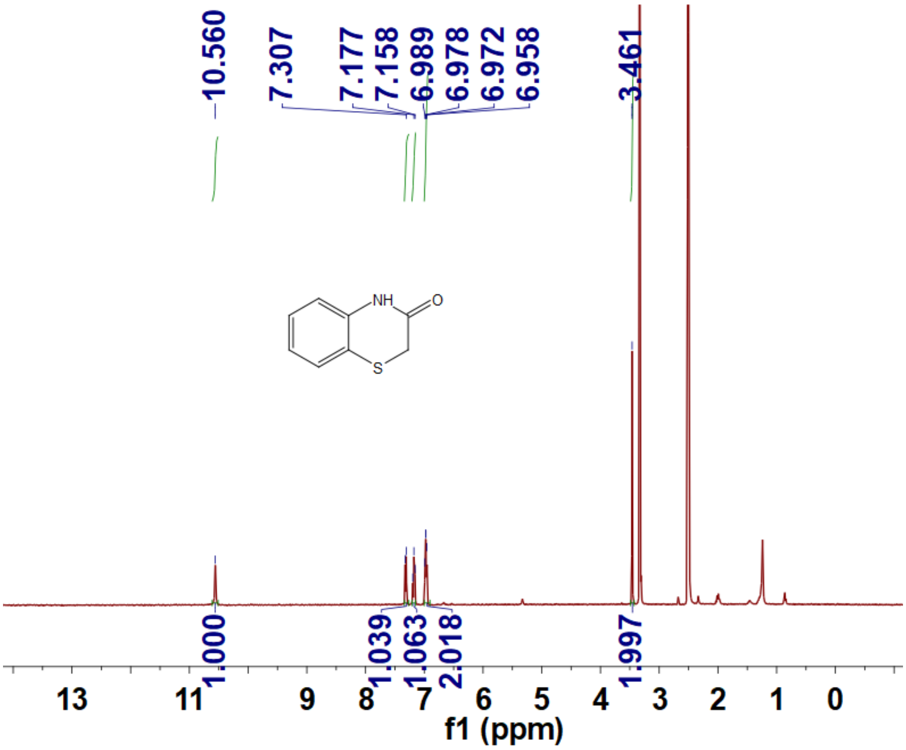

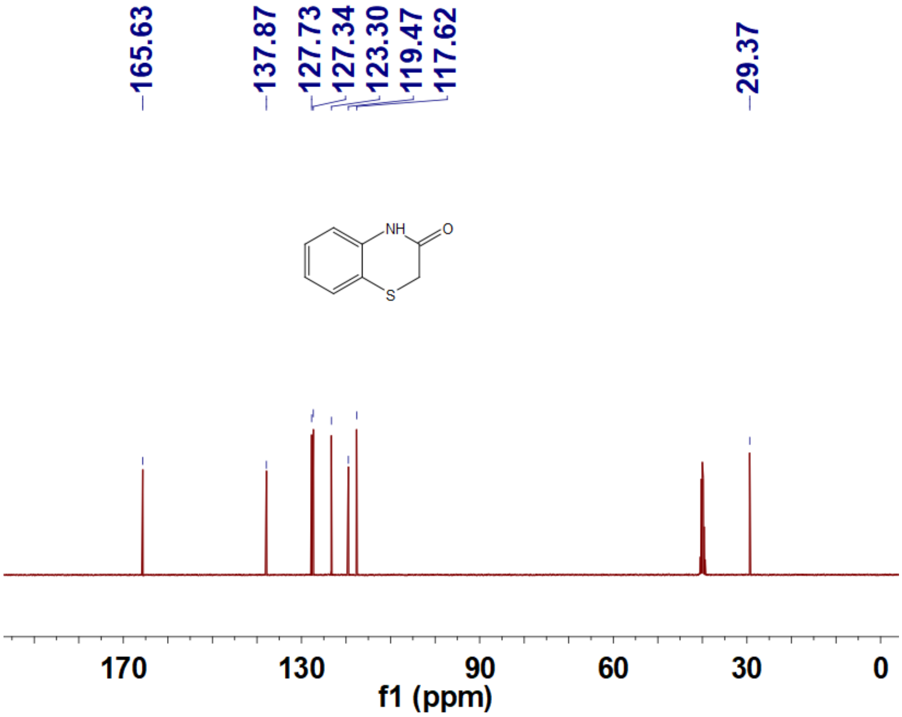

1,3-Benzothiazol-2-ylmethanol (3a)

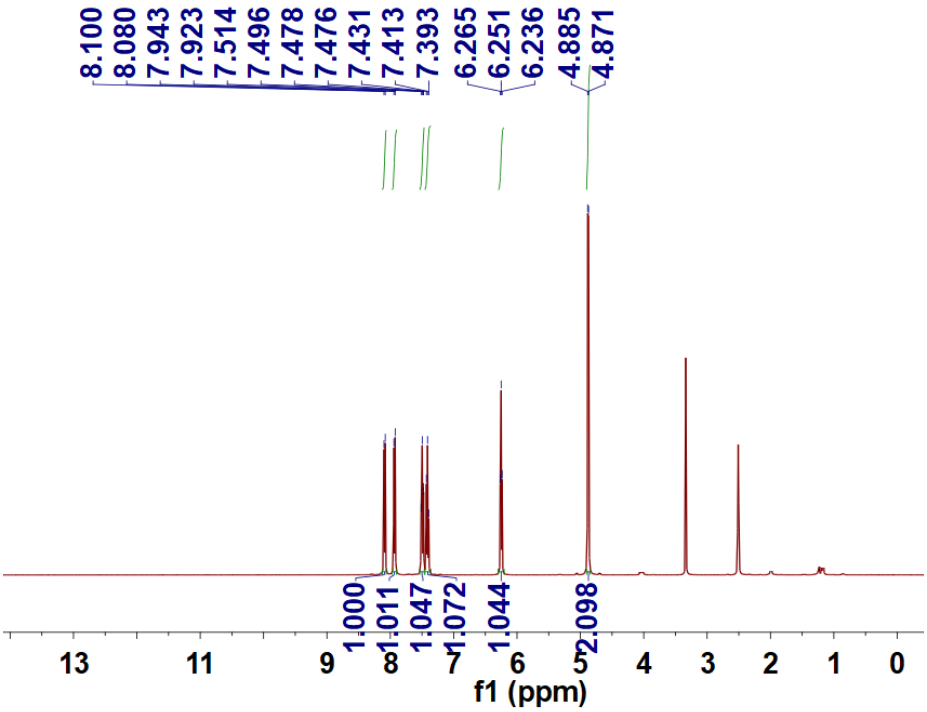

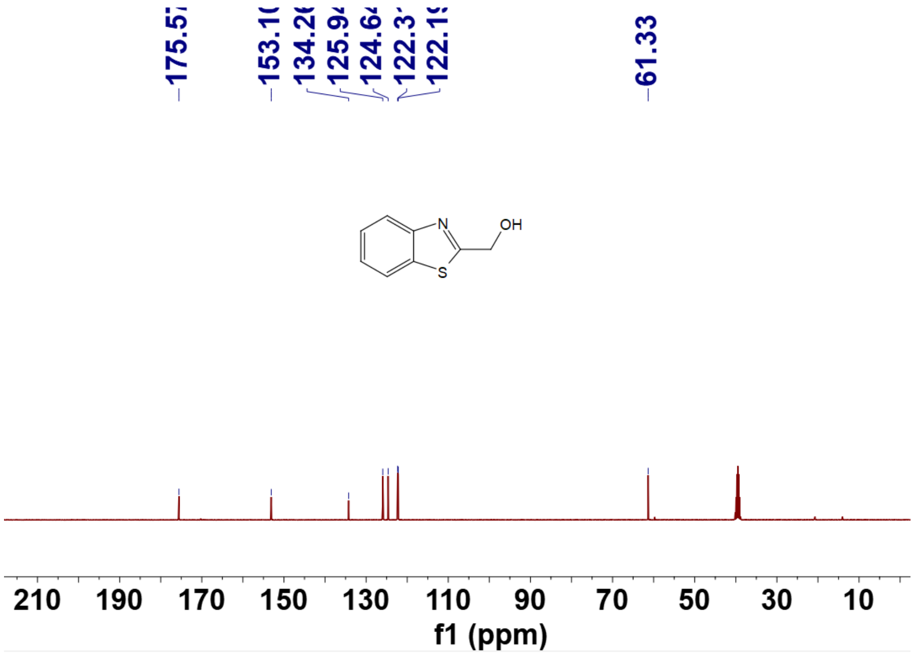

2-(Hydroxymethyl)benzimidazole (3b)

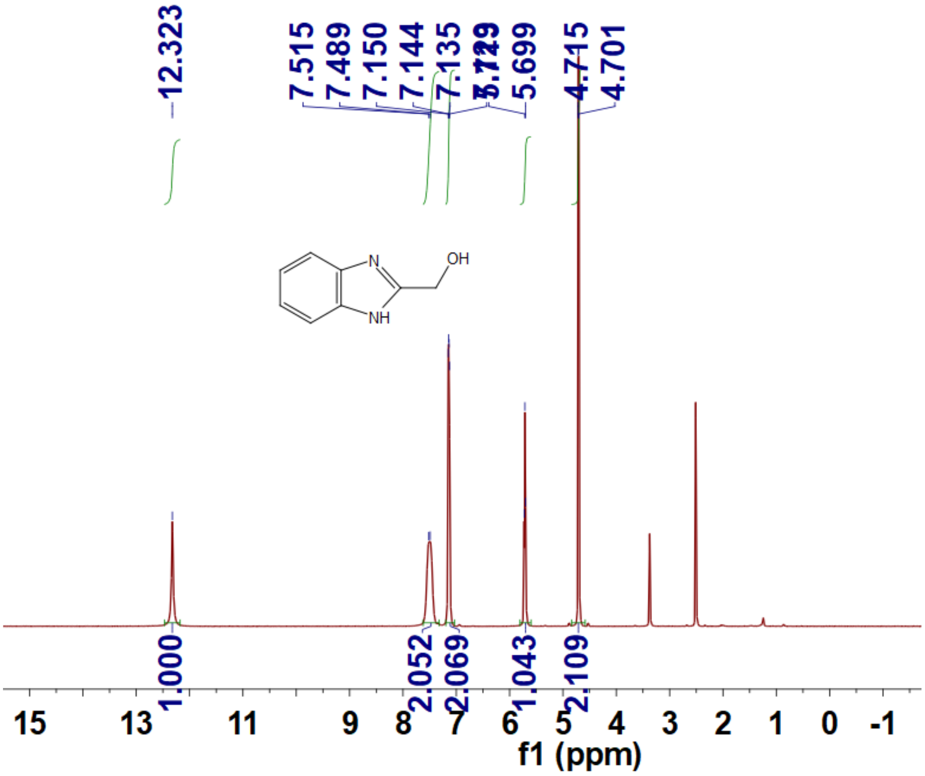

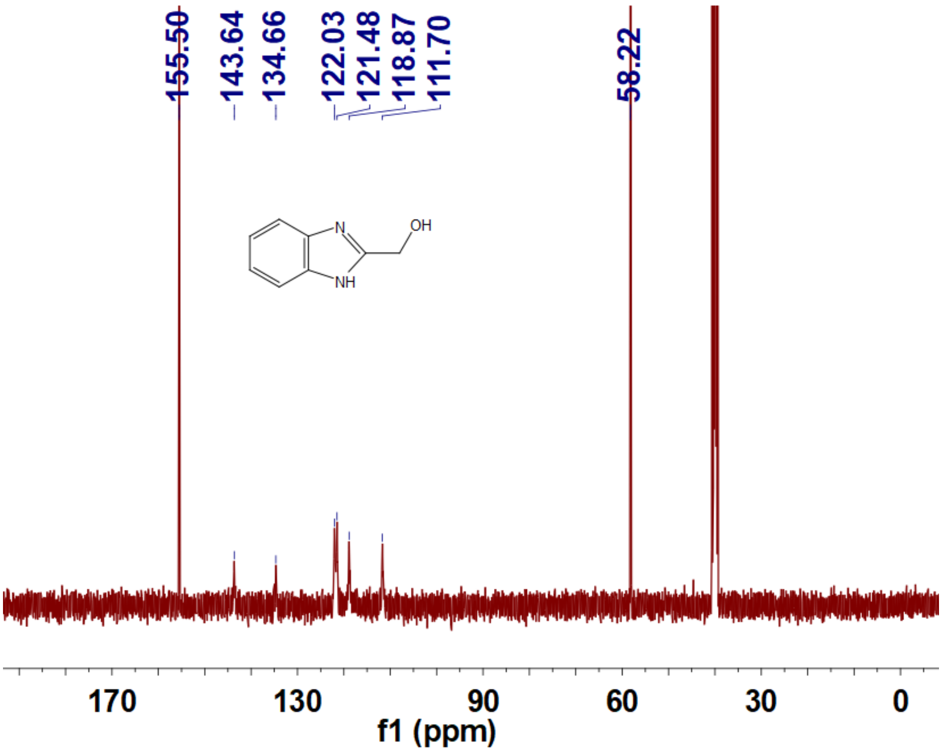

Glycolic acid (3c)

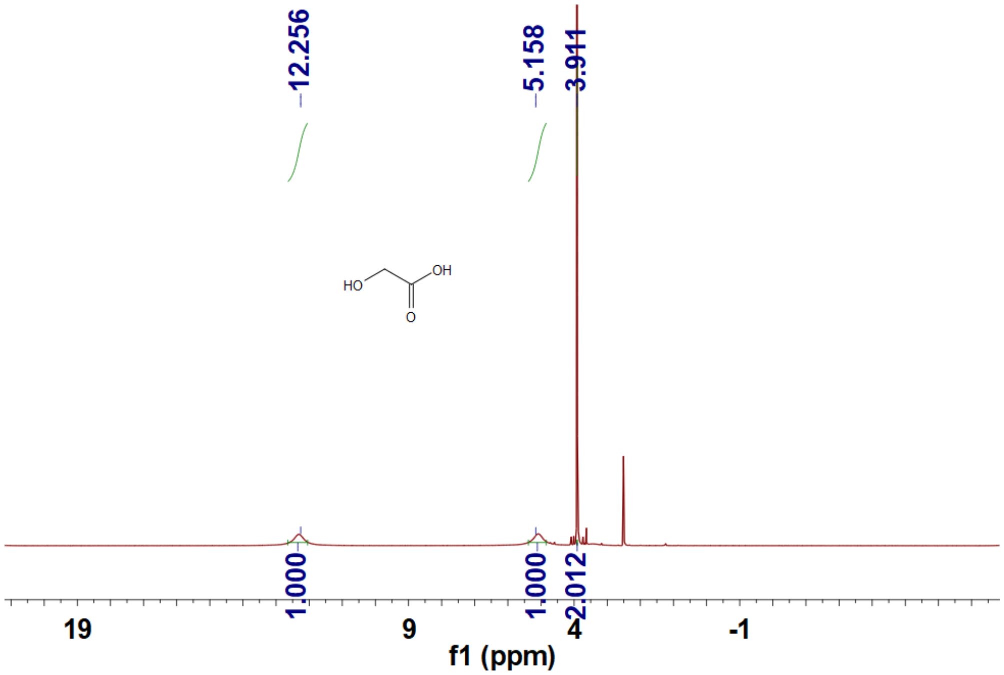

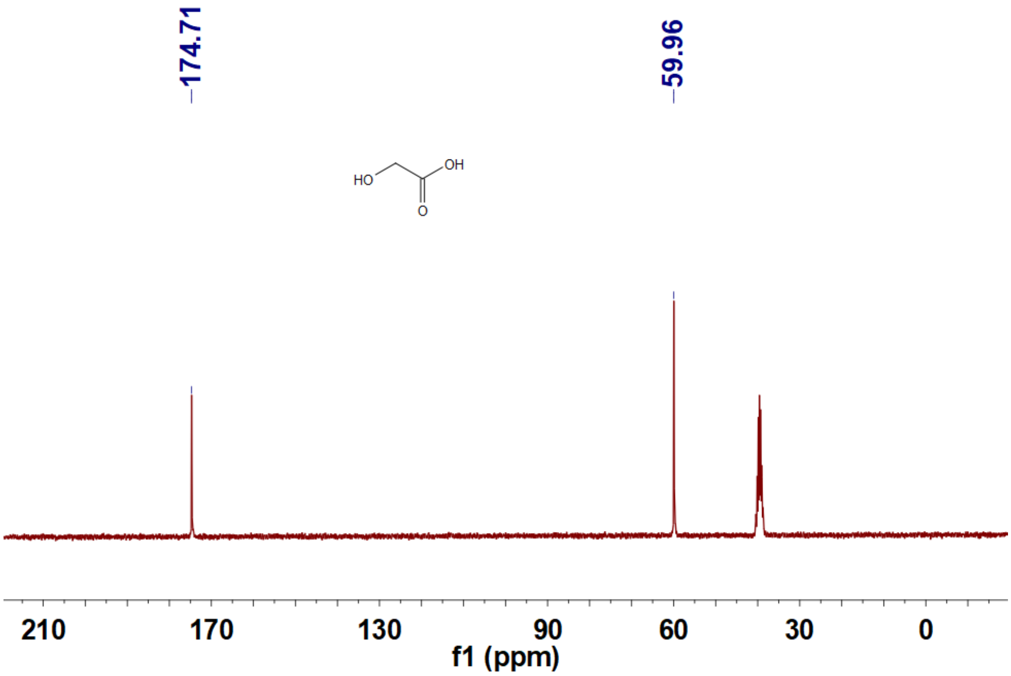

Glycolic acid methyl ester (3d)

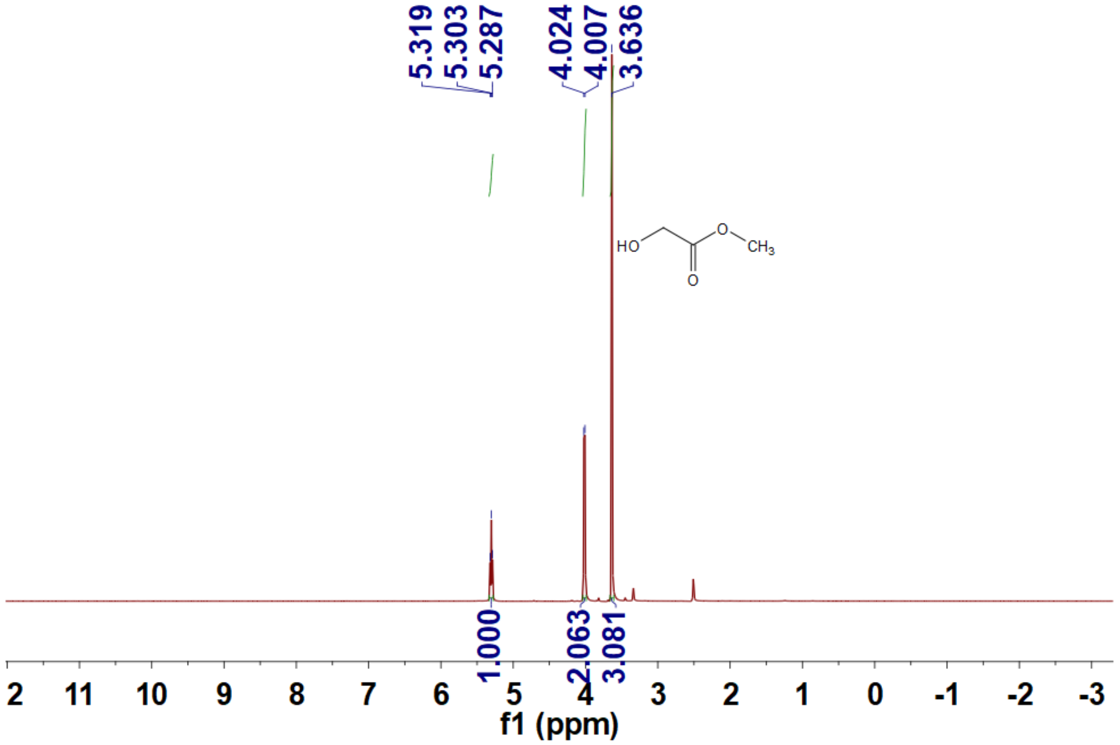

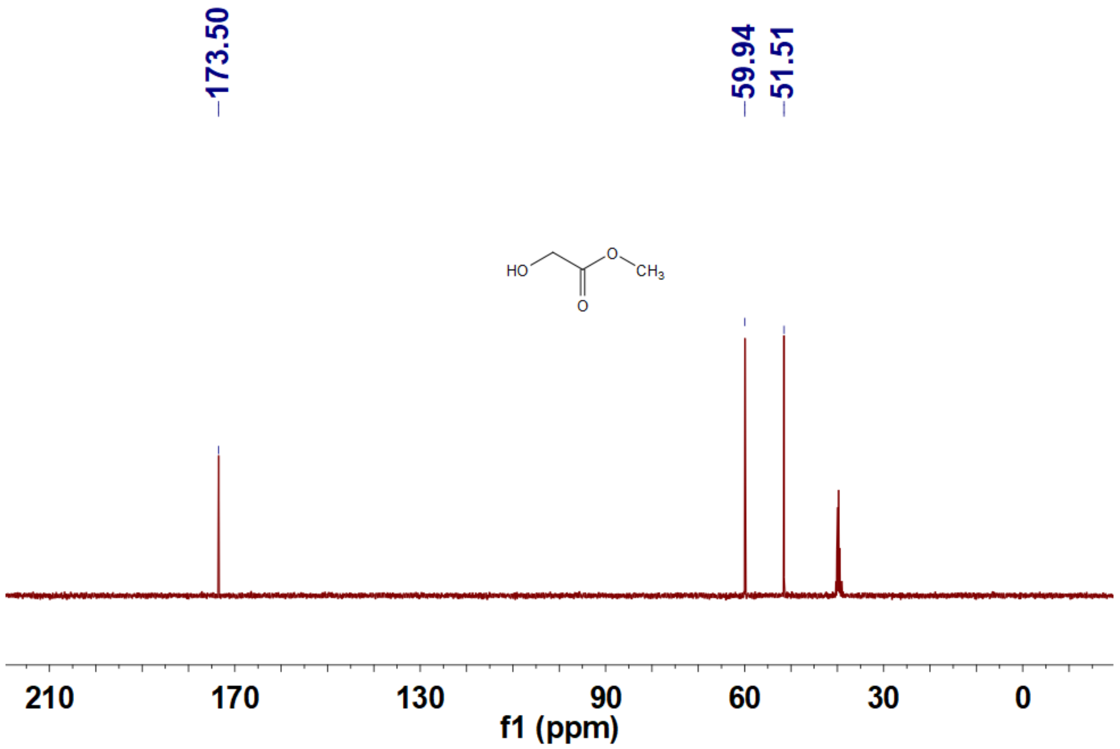

Glycolanilide (3e)

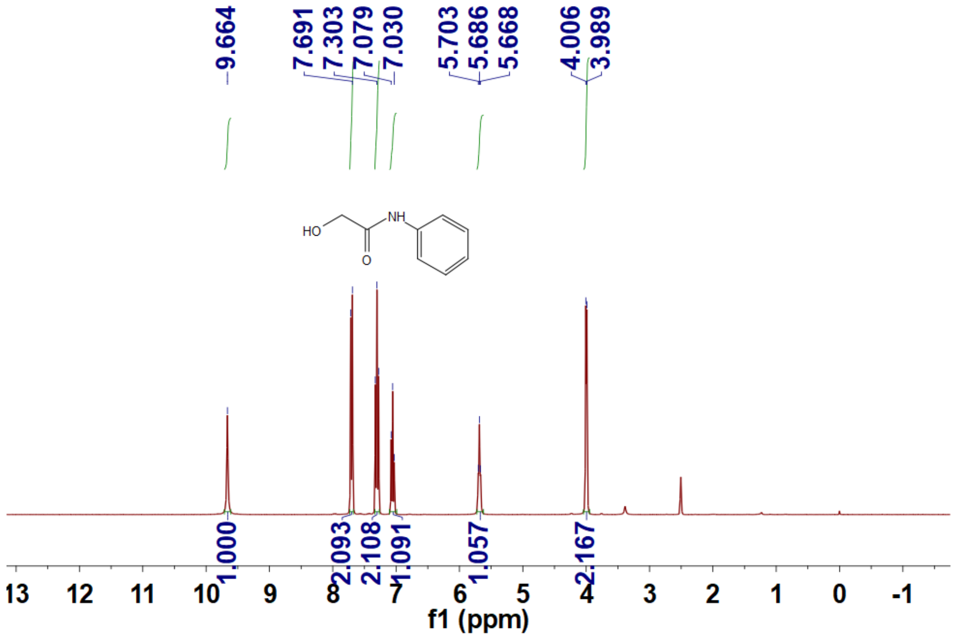

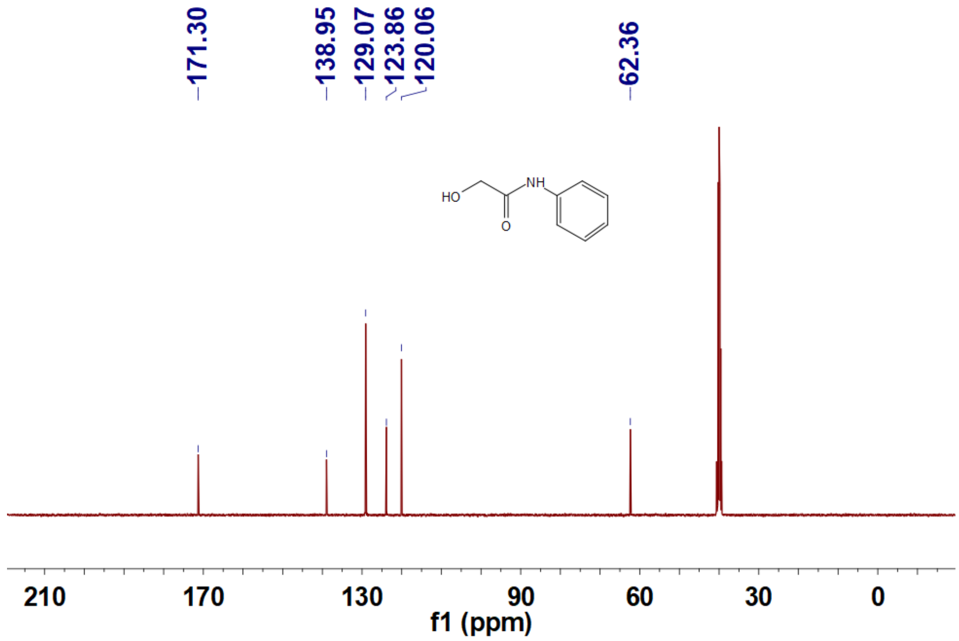

N, N-Diethyl-2-hydroxyacetamide (3f)

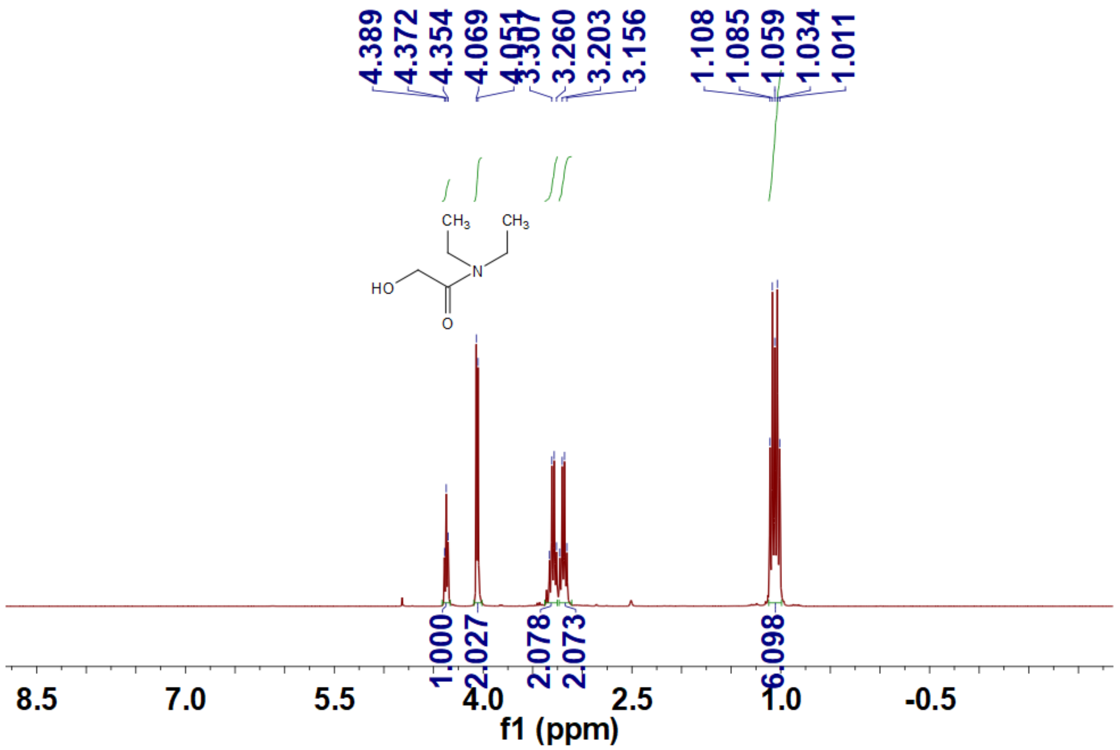

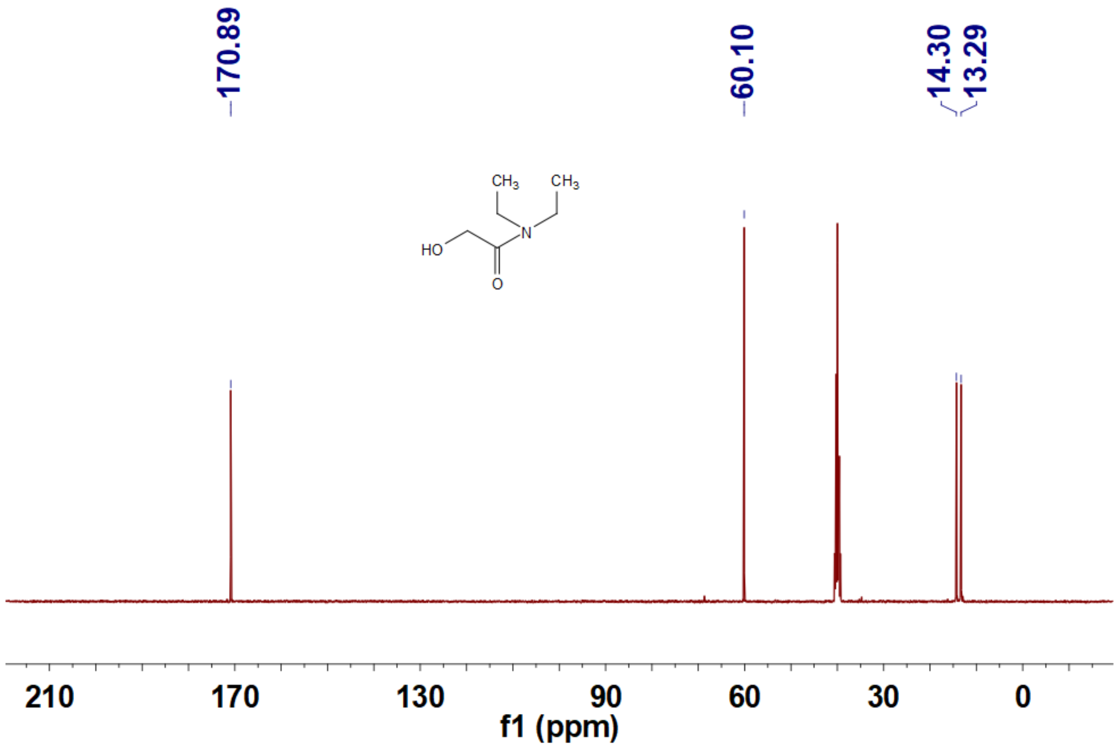

1-(Benzo[d]thiazol-2-yl)ethan-1-ol (3g)

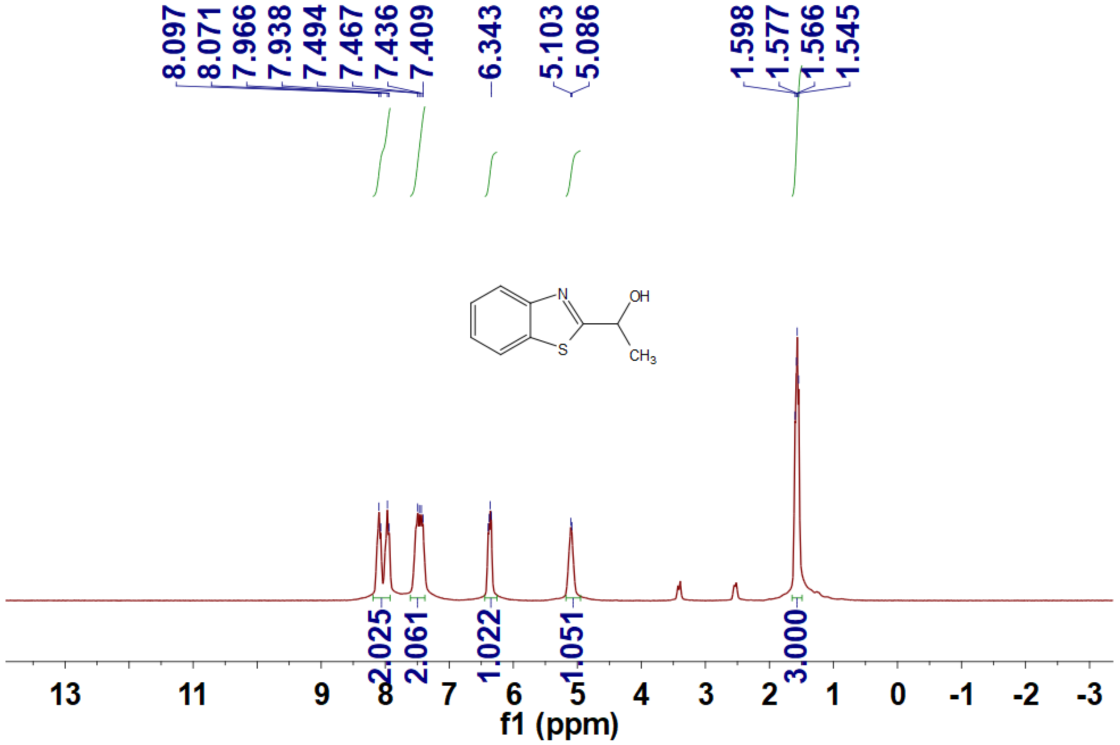

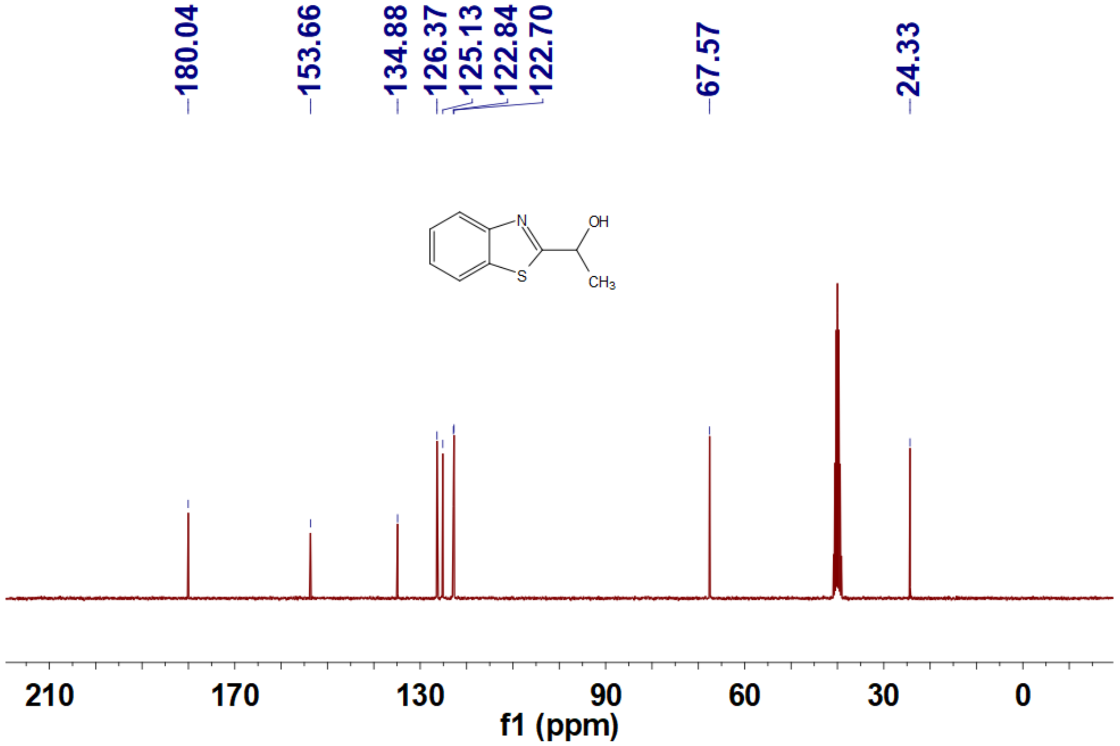

1-benzimidazol-2-ylethanol (3h)

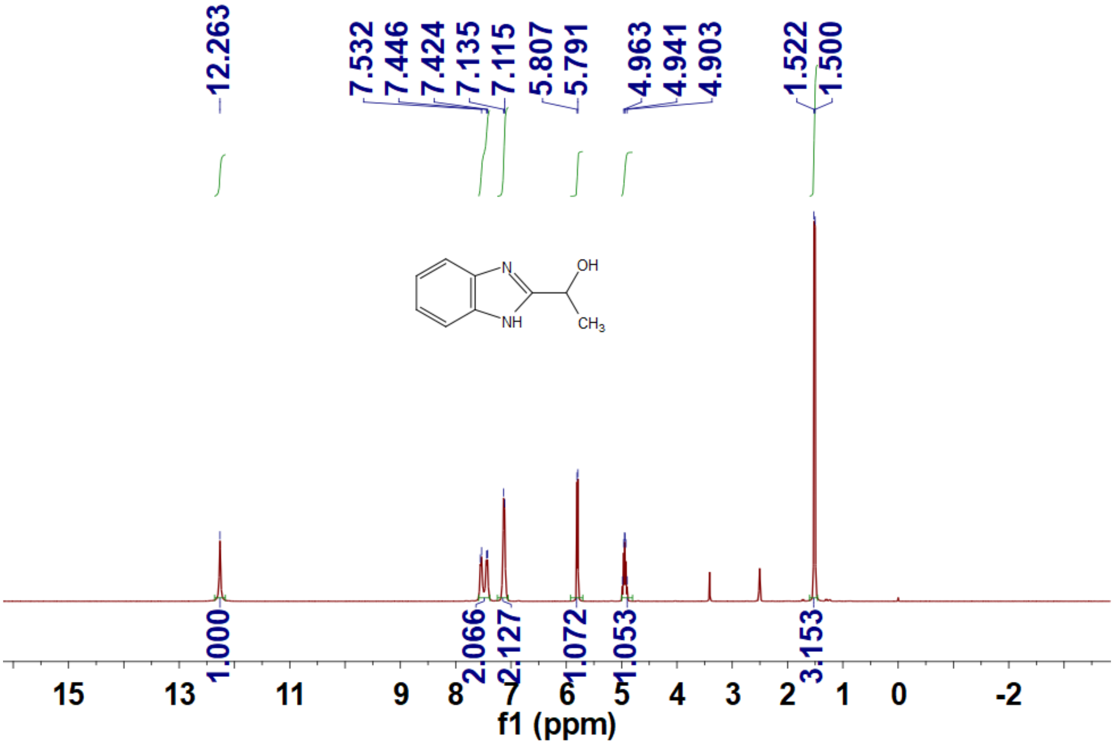

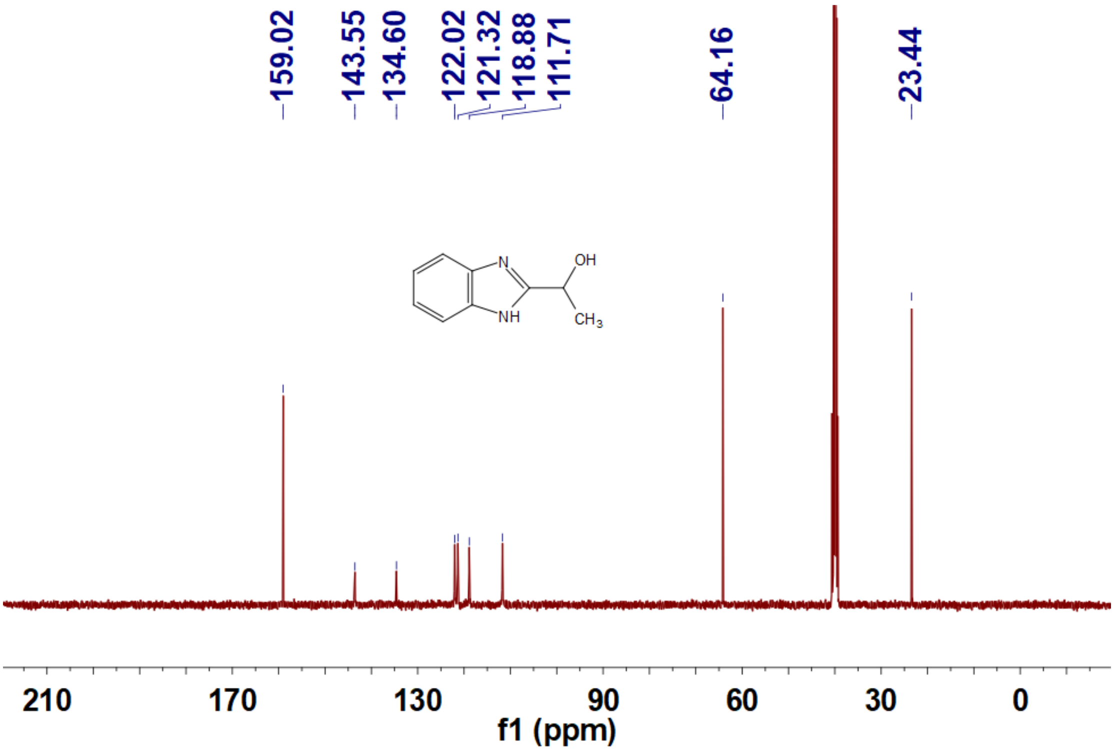

Lactanilide (3i)

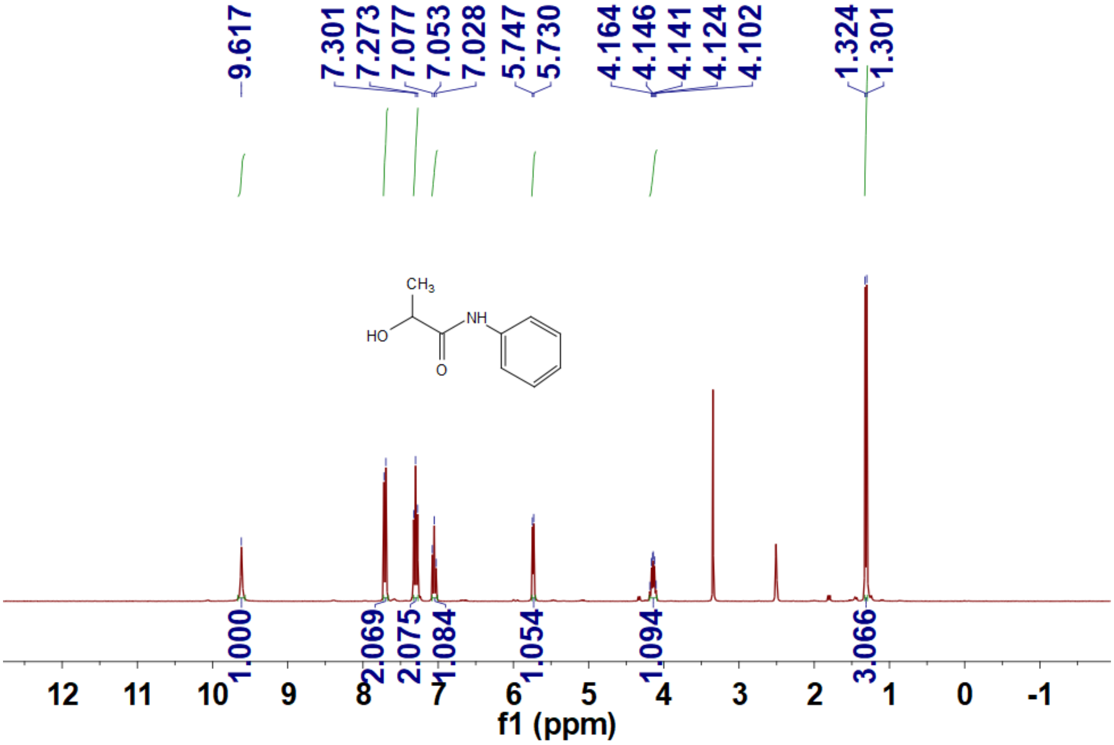

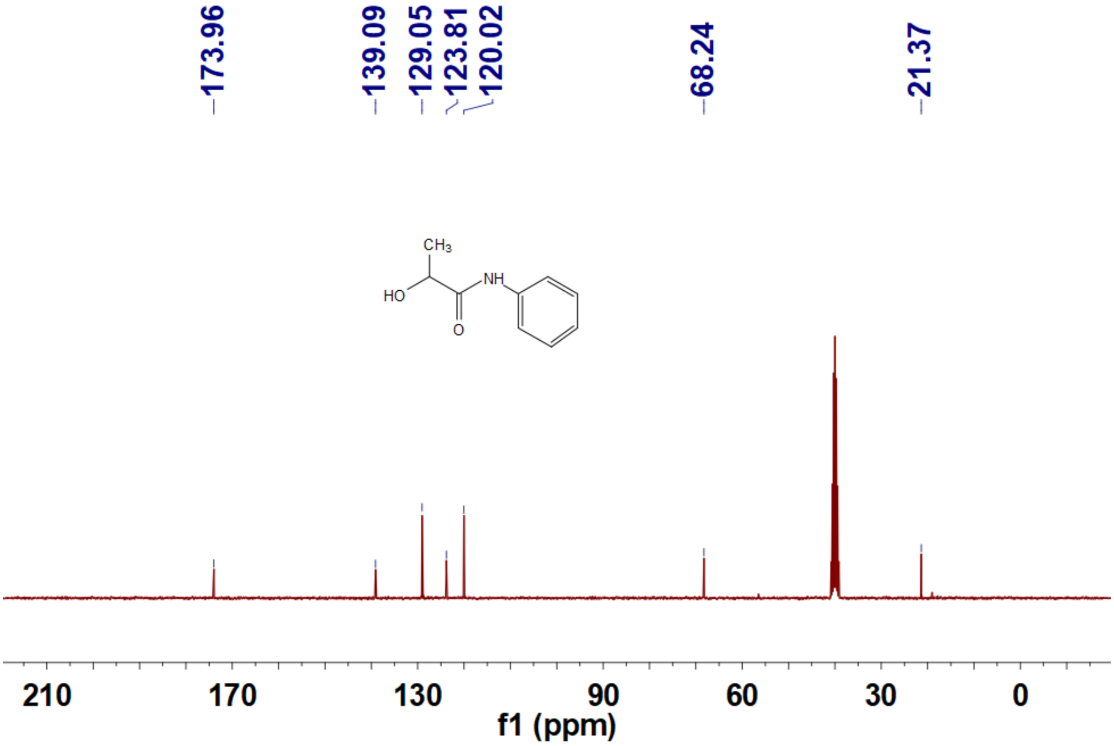

Lactic acid (3g)

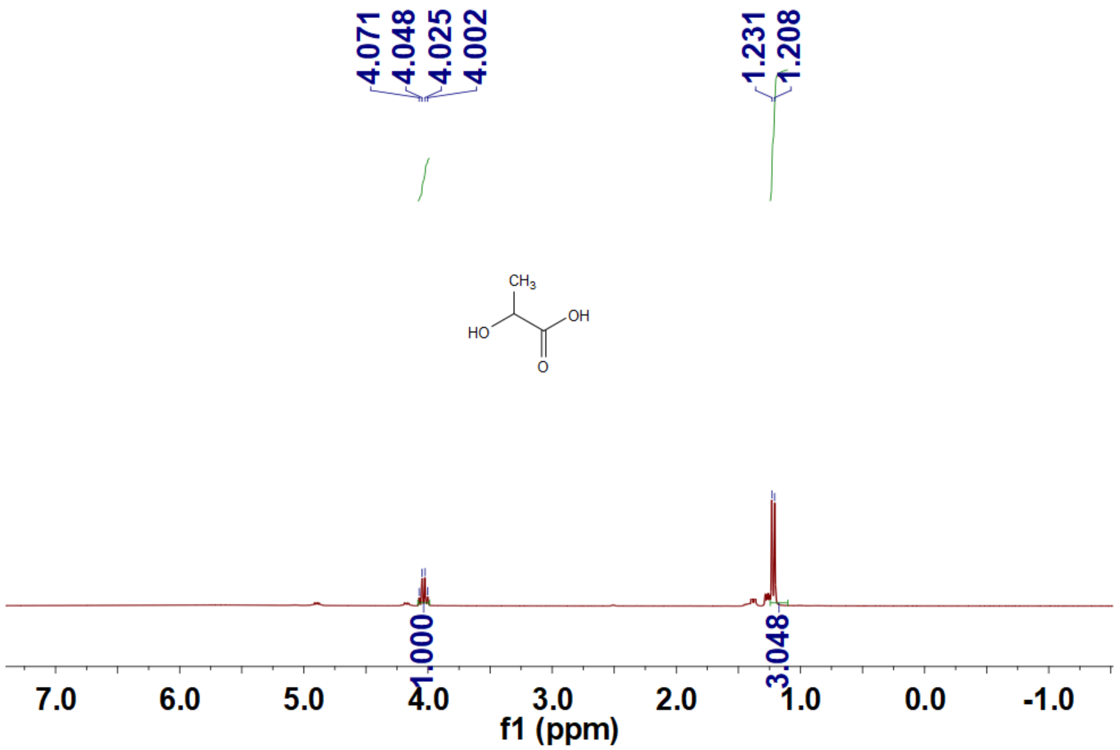

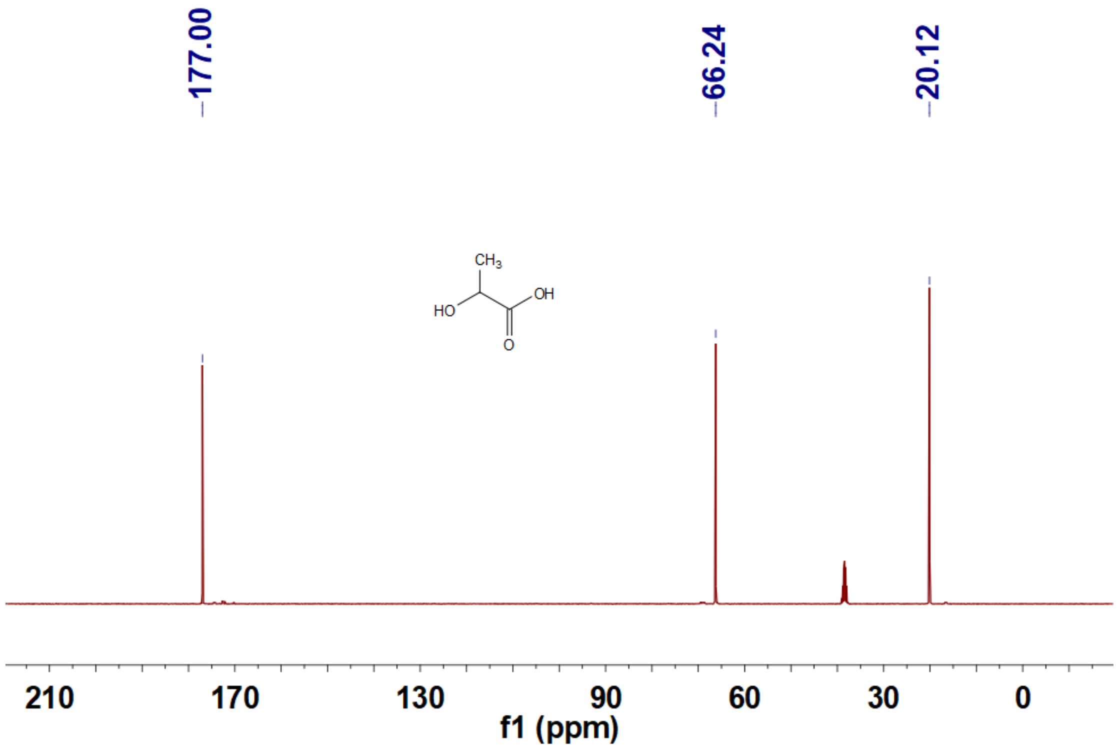

Methyl lactate (3k)

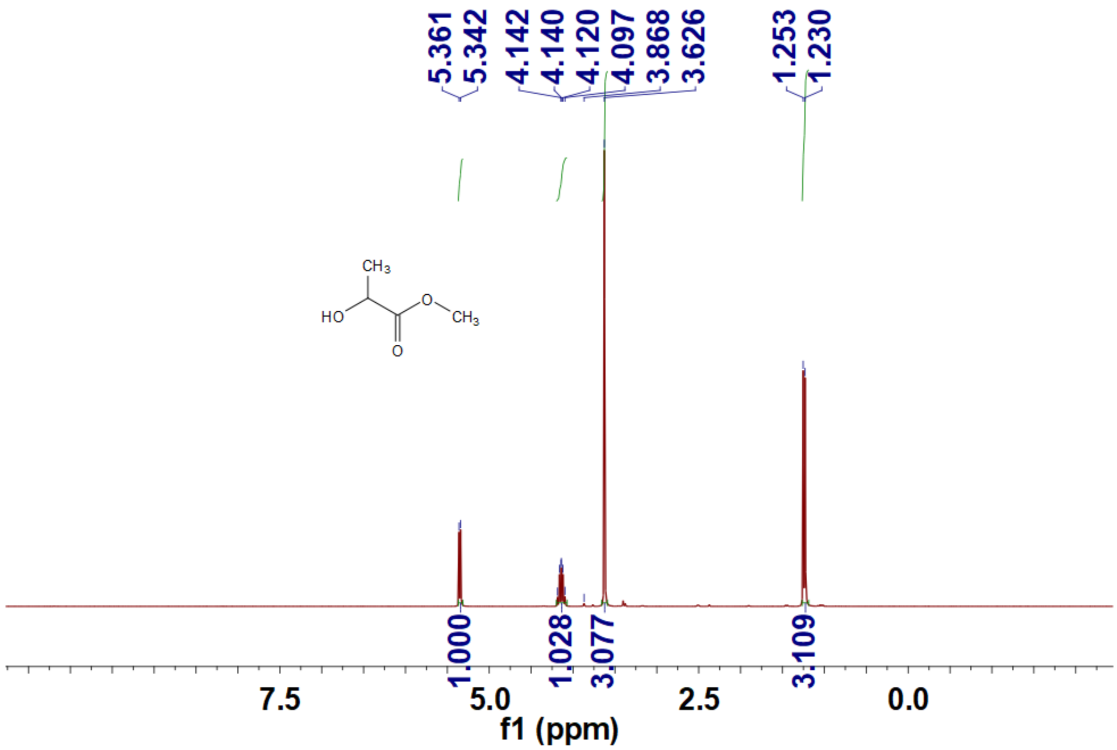

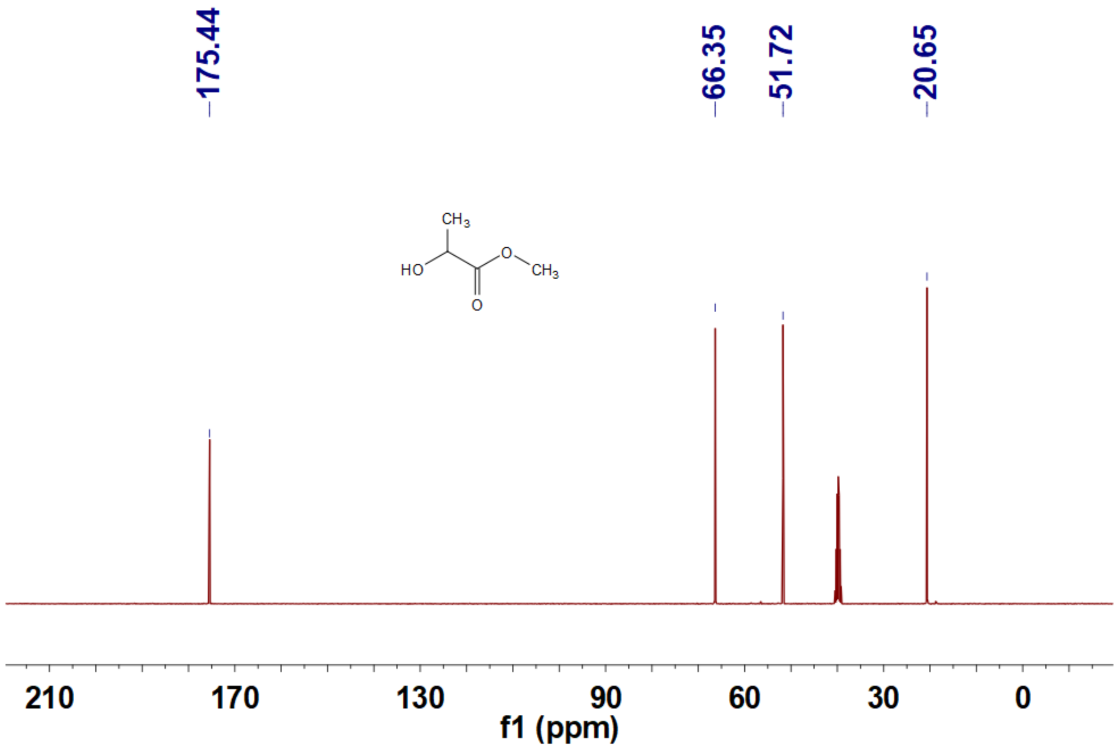

Ethyl lactate (3l)

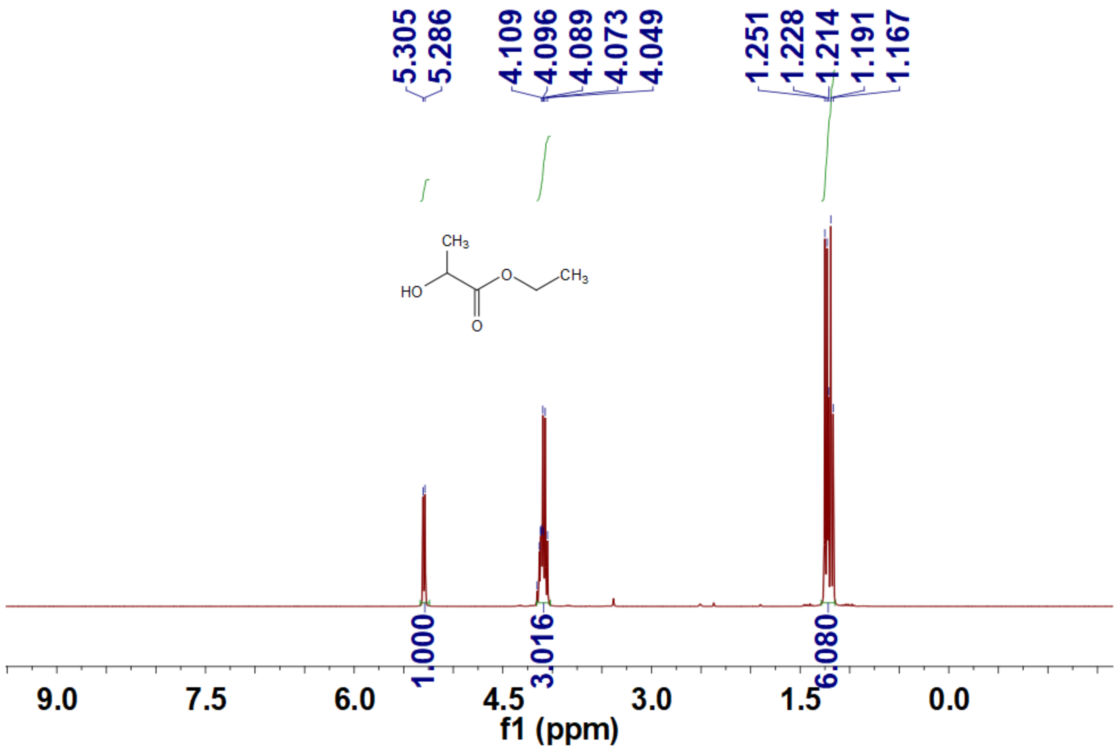

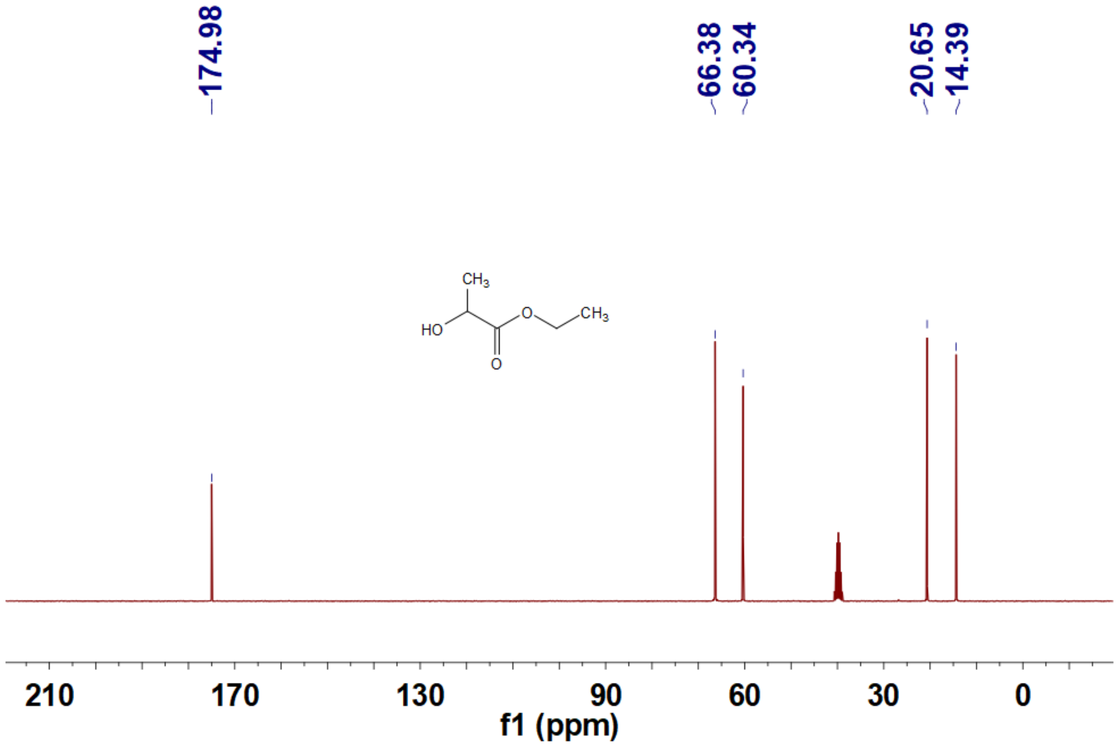

2-Hydroxy-1-(pyrrolidin-1-yl)propan-1-one (3m)

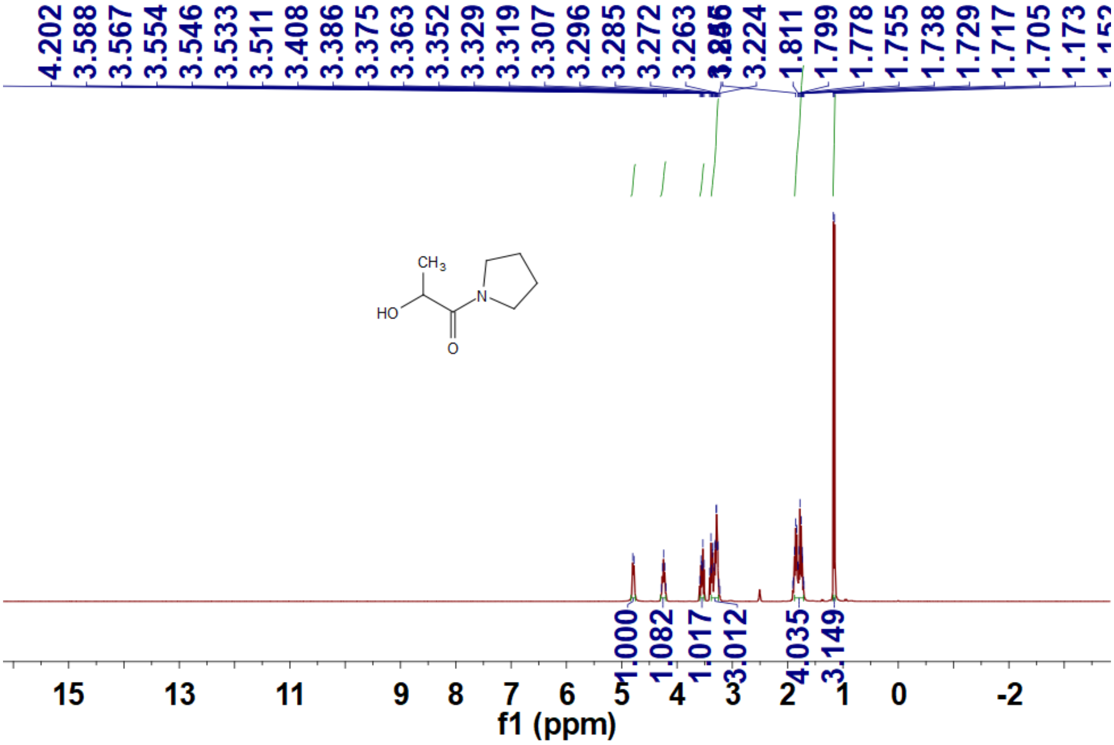

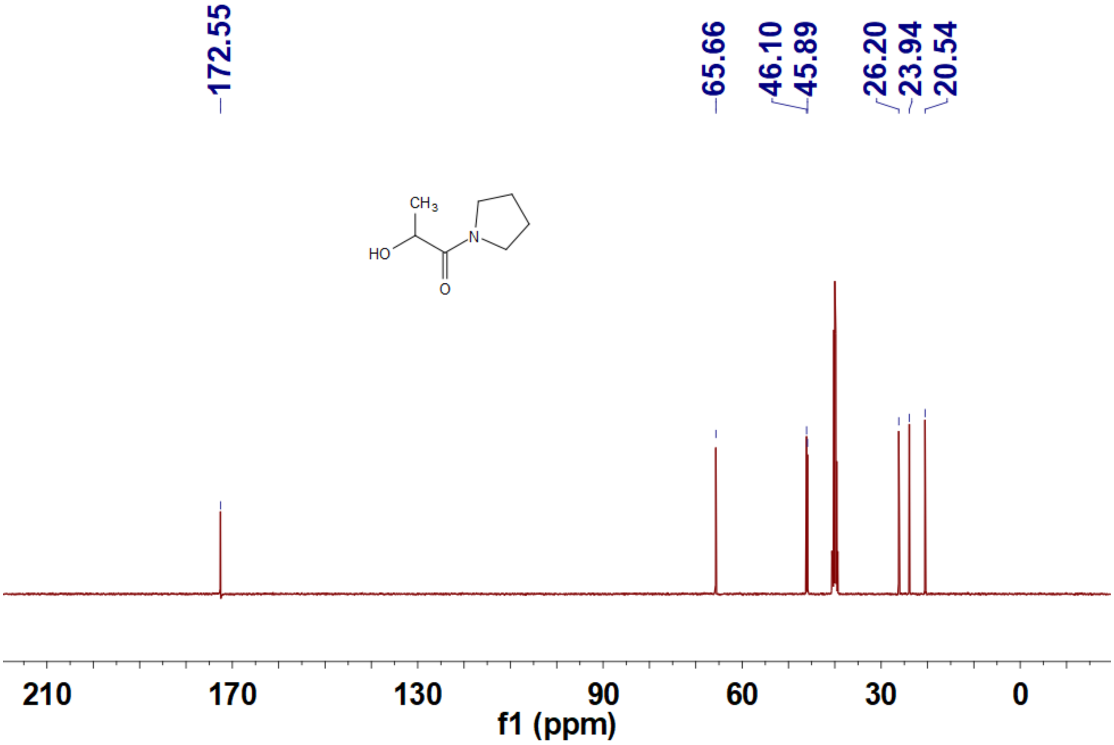

Methyl crotonate (3n)

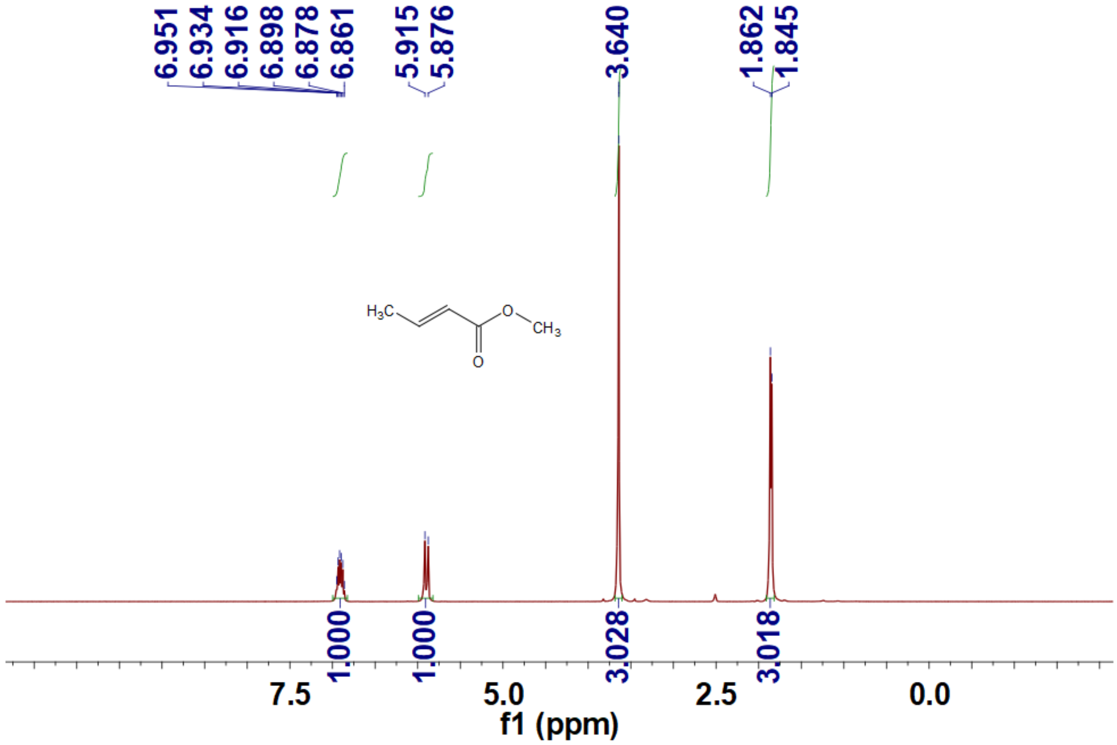

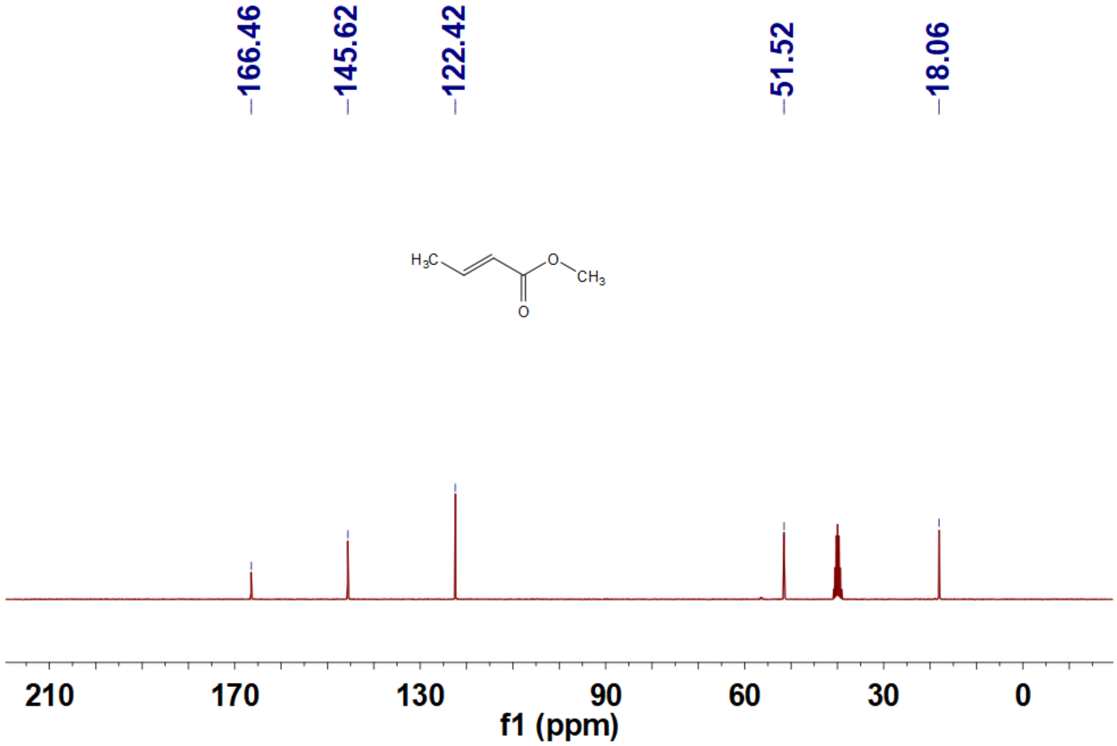

Ethyl crotonate (3o)

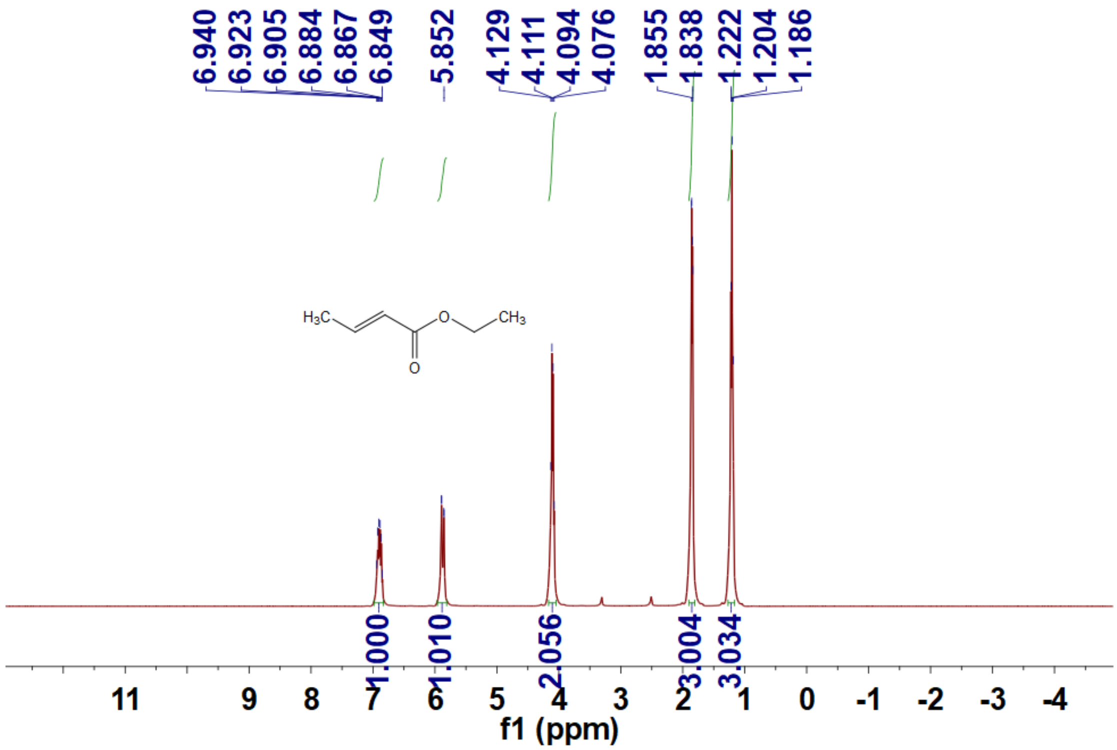

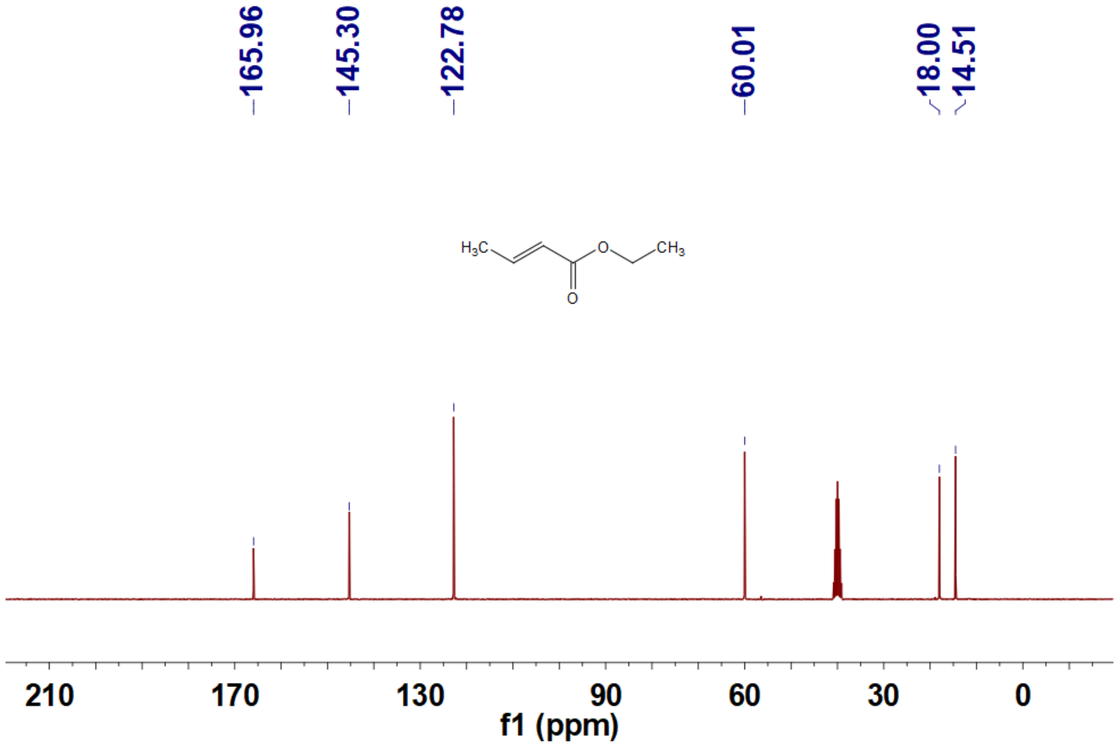

(2E)-N-Benzylbut-2-enamide (3p)

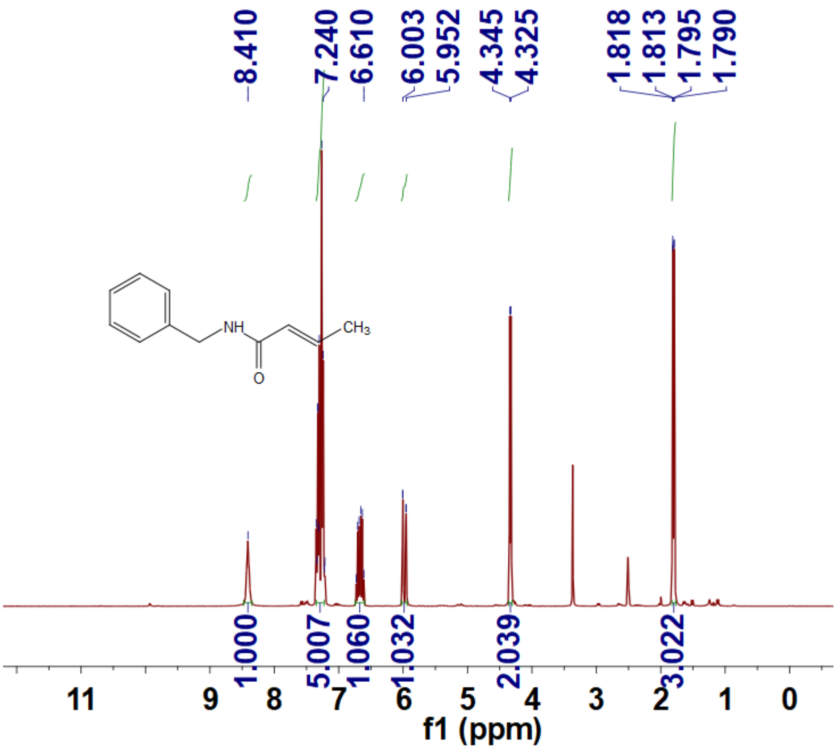

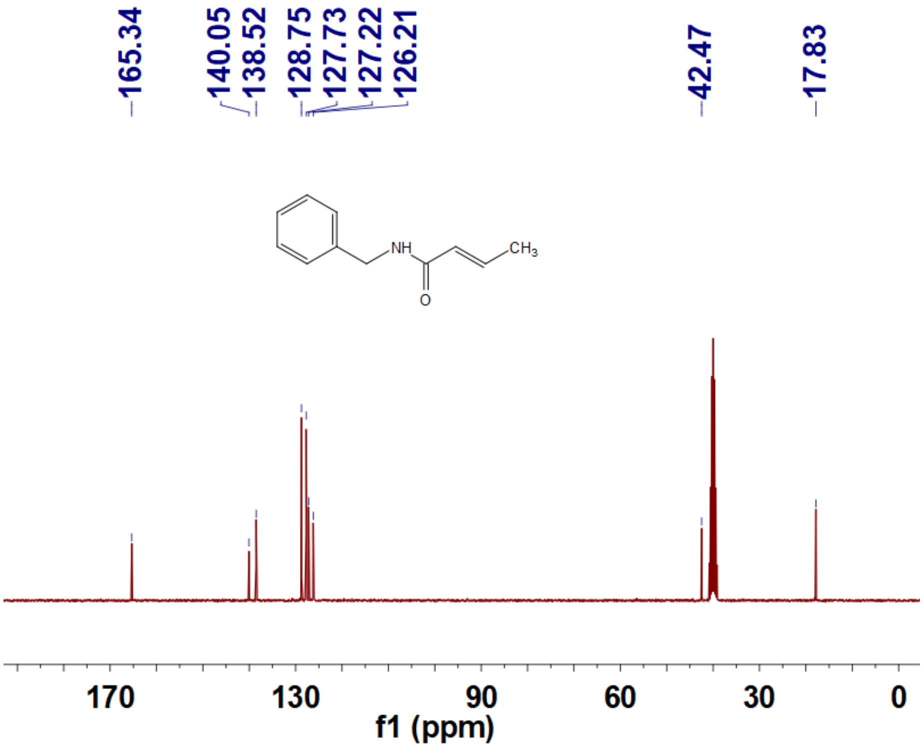

4-Methyl-1,3,4,5-tetrahydro-2H-1,5-benzodiazepin-2-one (3q)

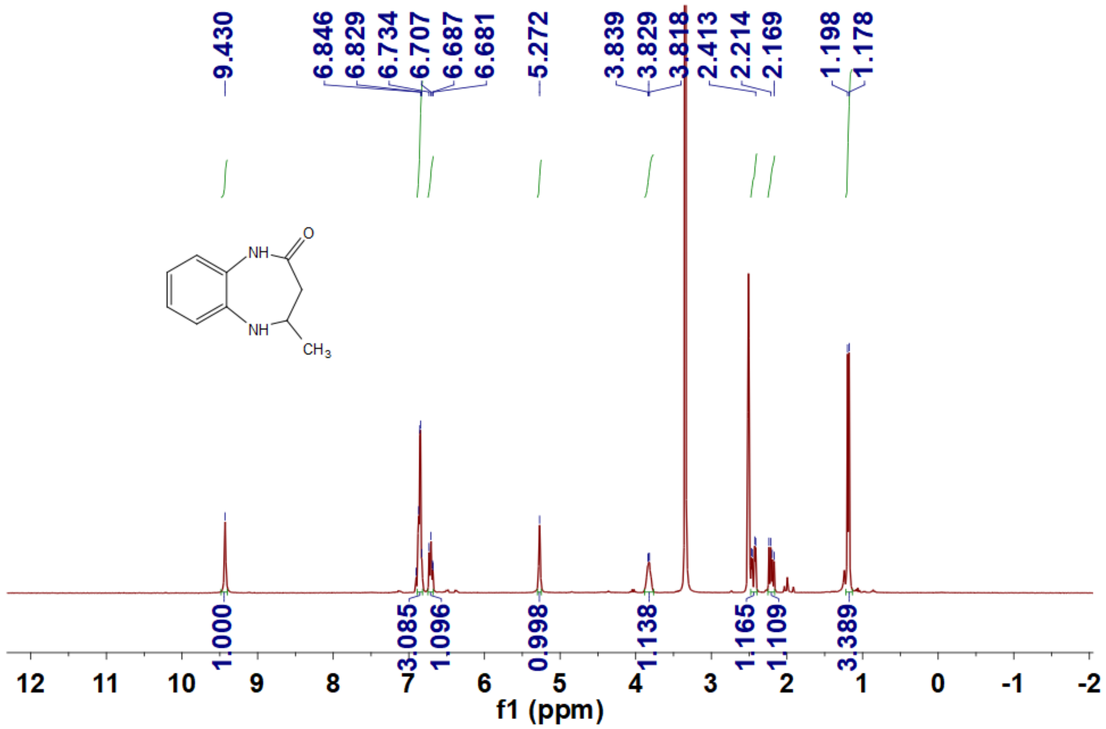

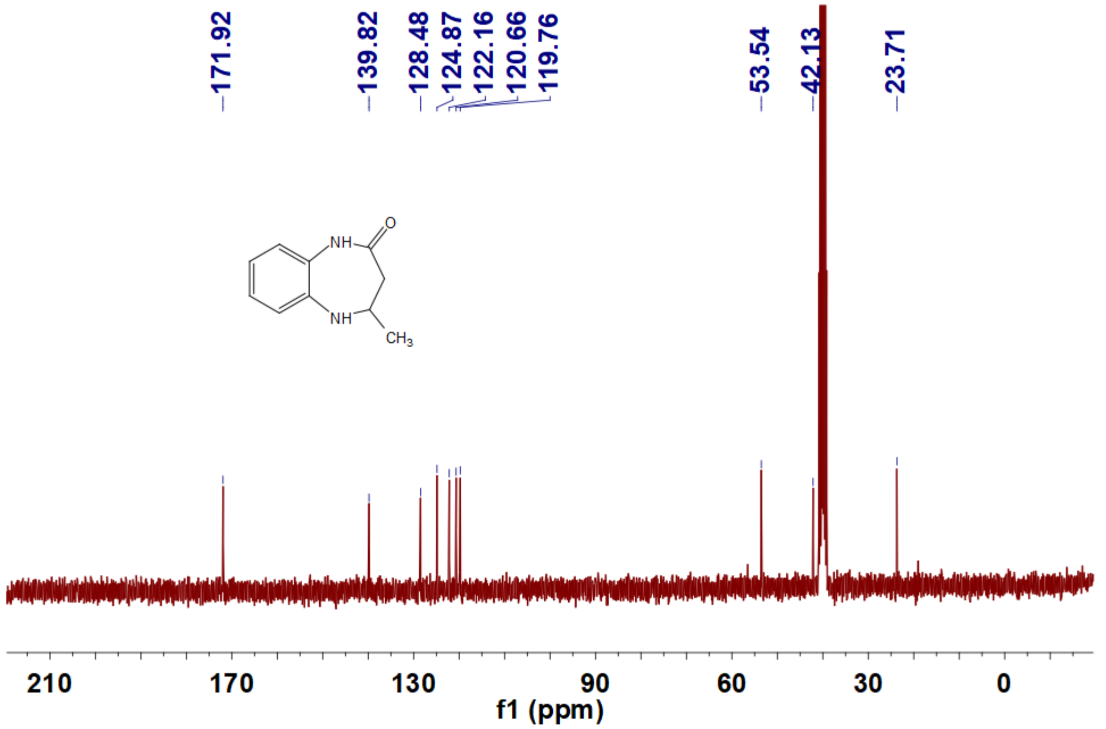

7-Fluoro-4-methyl-1,3,4,5-tetrahydro-2H-benzo[*b*][1,4]diazepin-2-one (3r)

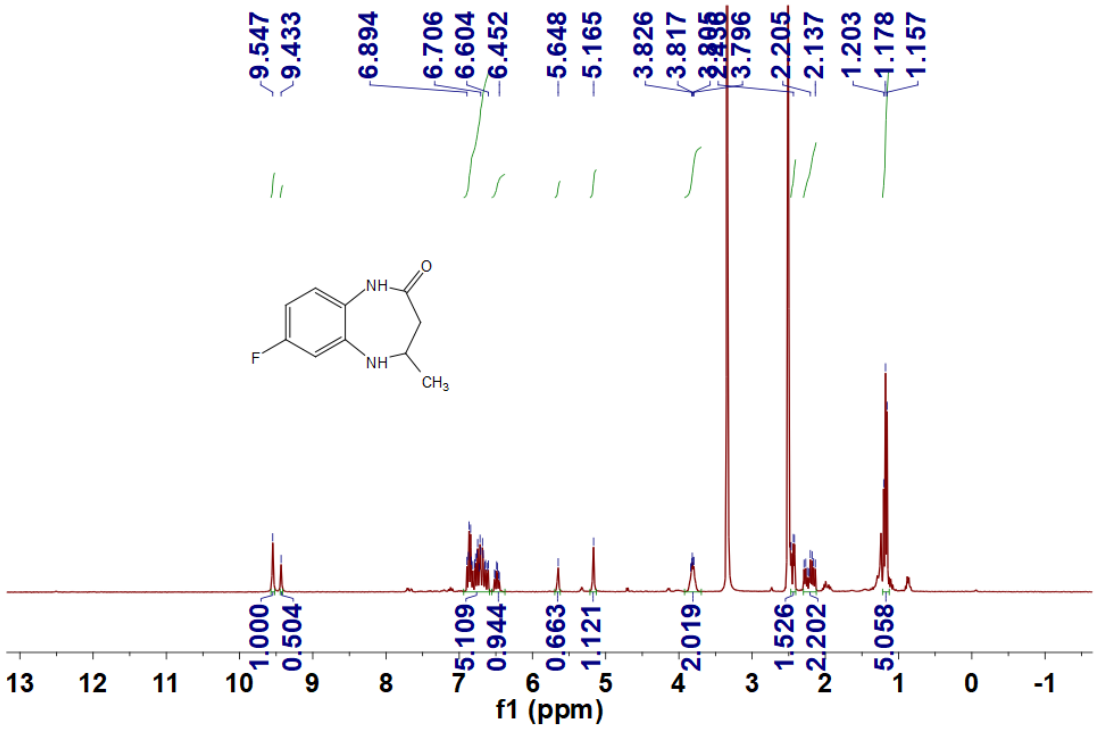

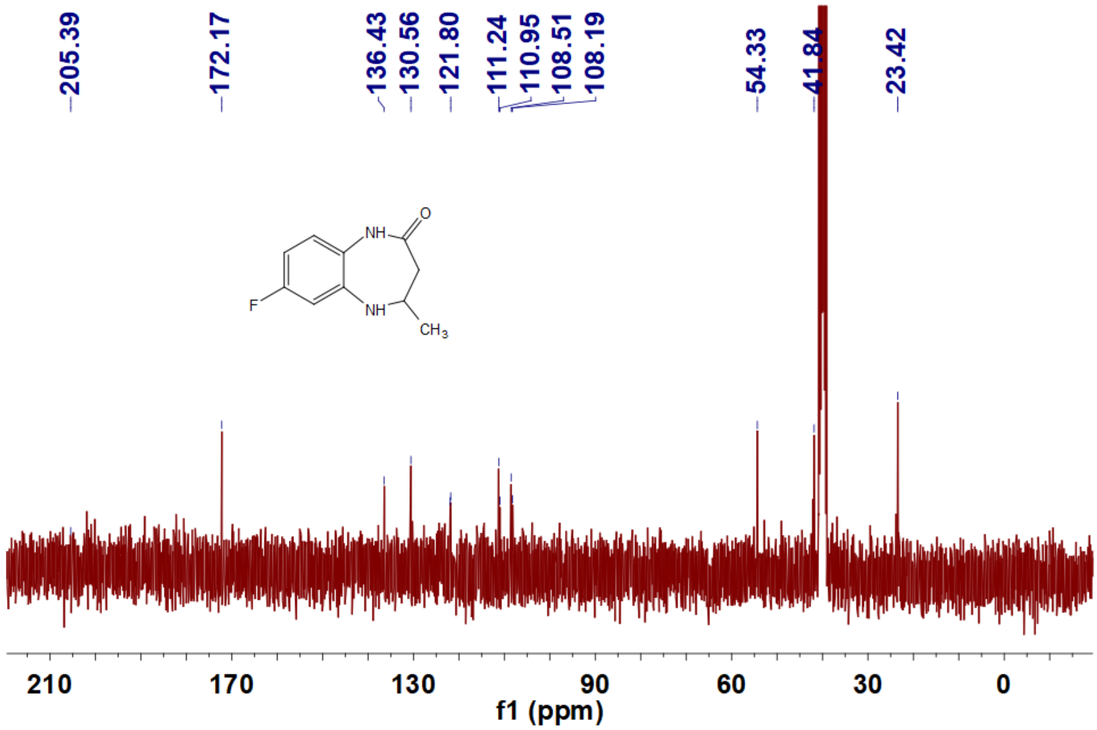

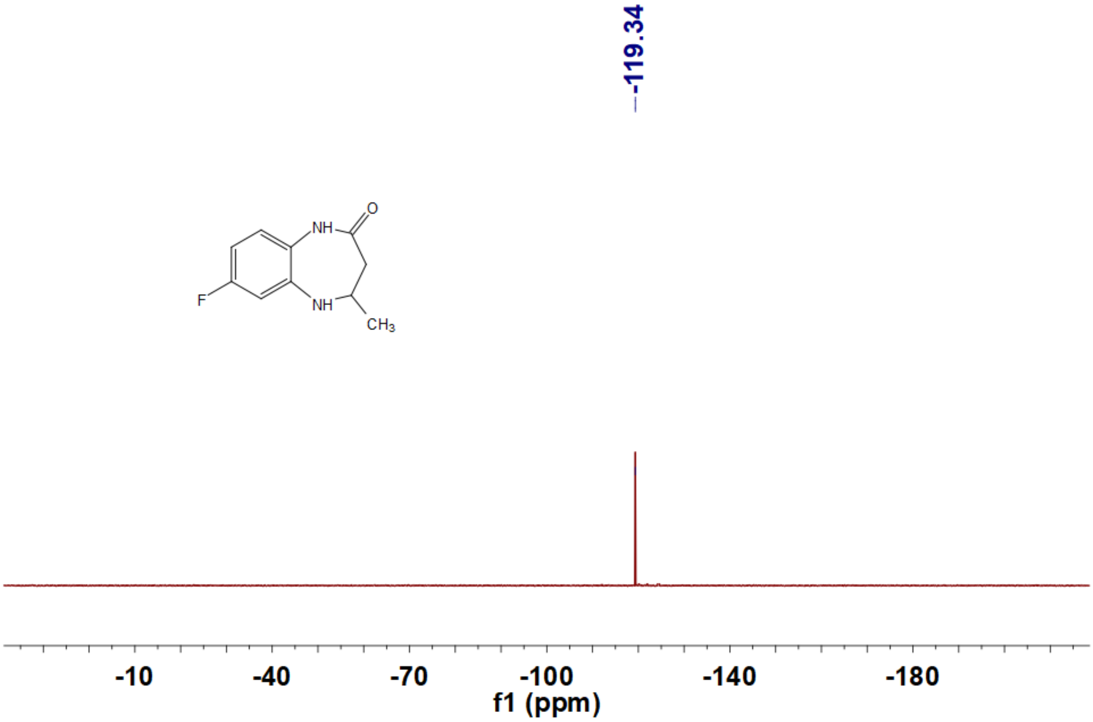

7-Chloro-4-methyl-1,3,4,5-tetrahydro-2*H*-benzo[*b*][1,4]diazepin-2-one (3s)

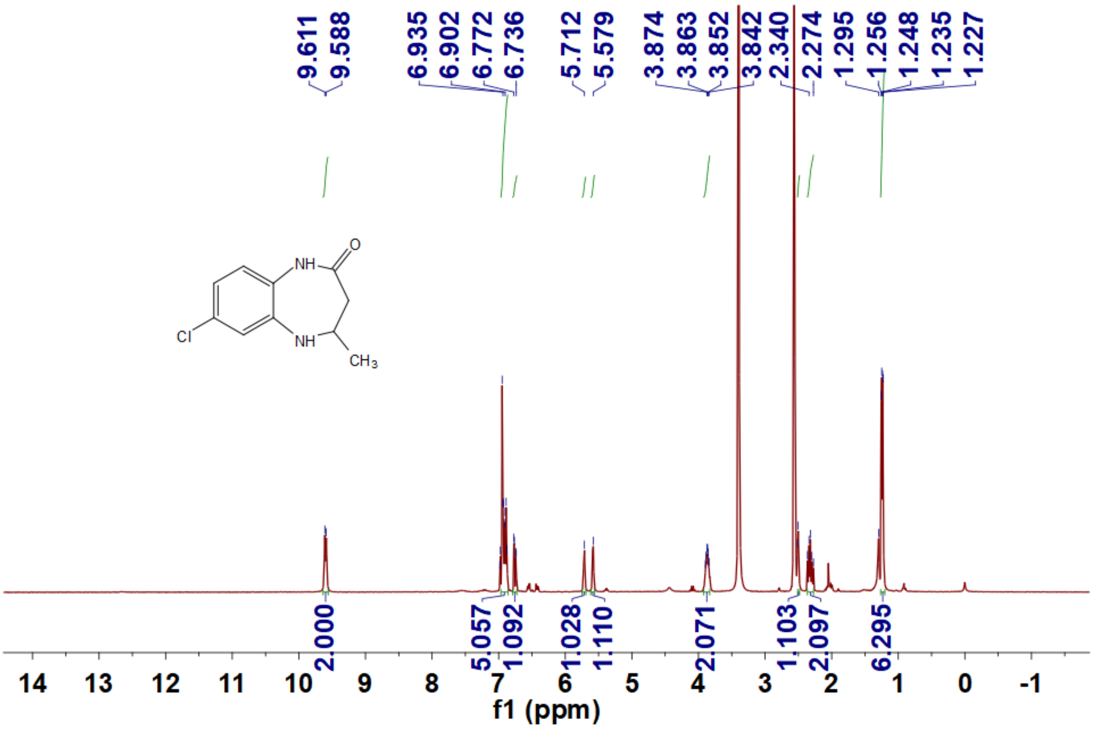

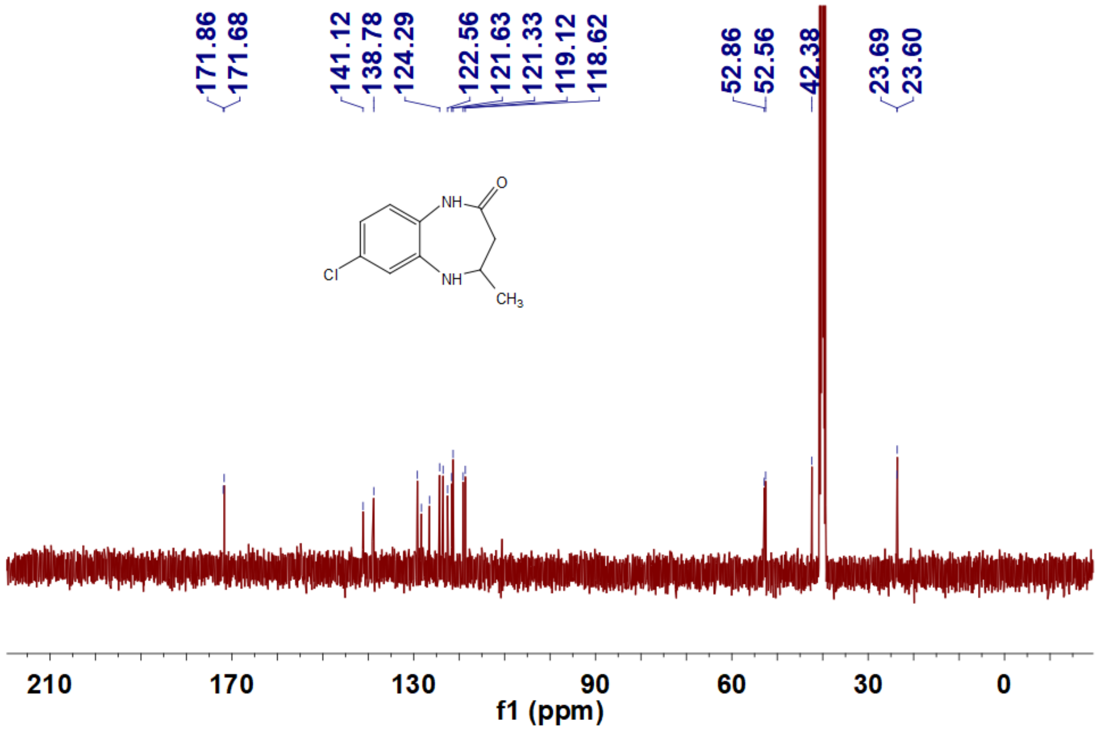

7-Bromo-4-methyl-1,3,4,5-tetrahydro-2H-benzo[b][1,4]diazepin-2-one (3t)

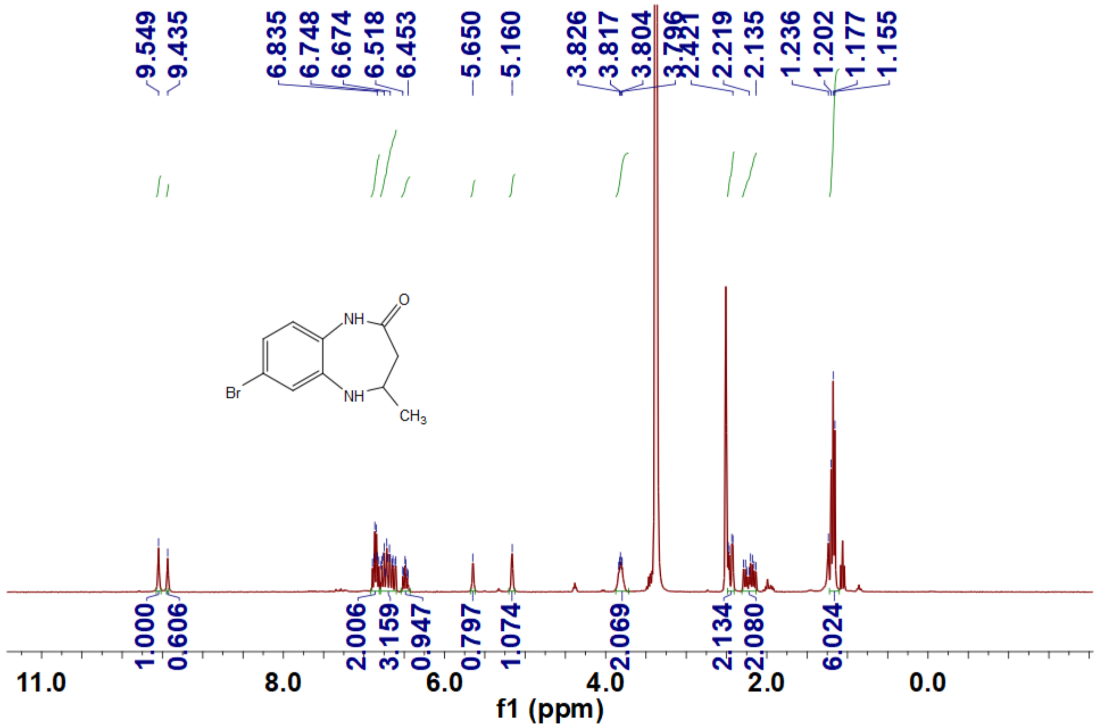

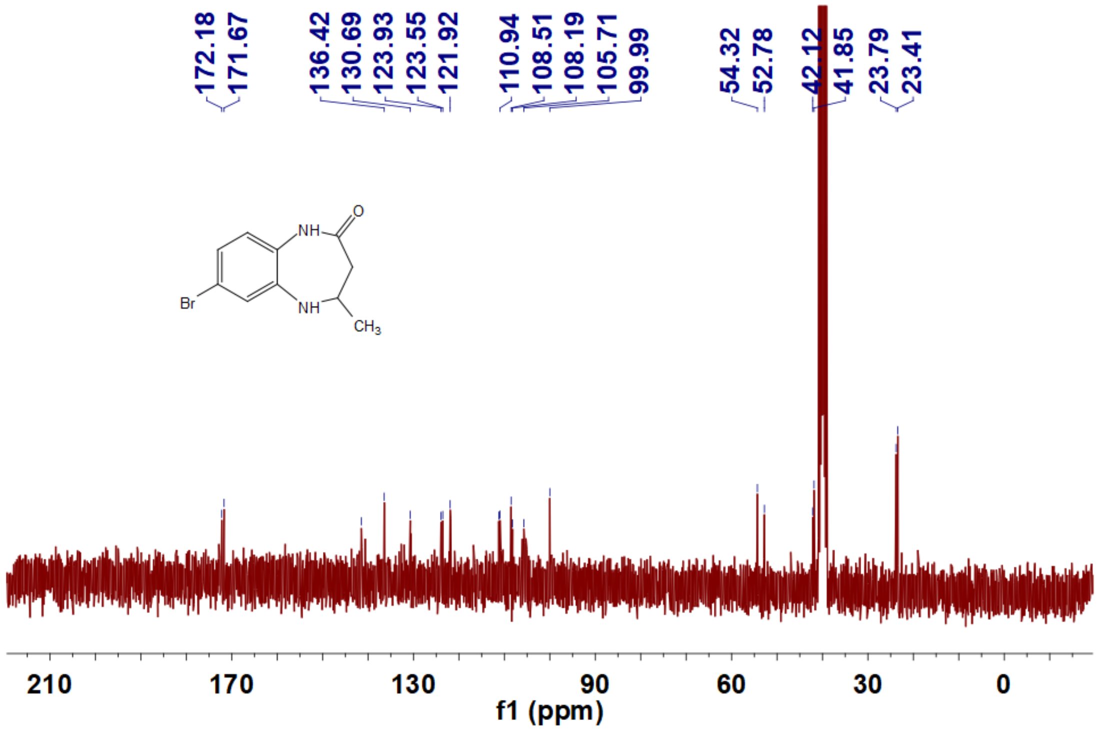

4,7-Dimethyl-1,3,4,5-tetrahydro-2H-benzo[b][1,4]diazepin-2-one (3u)

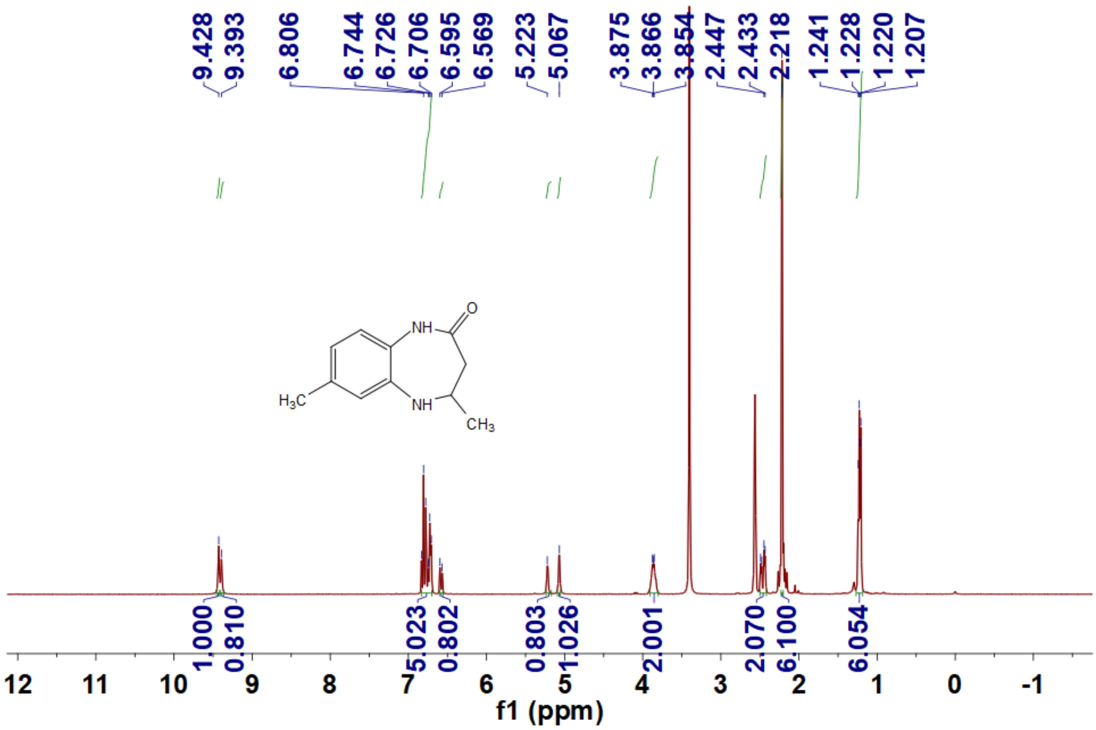

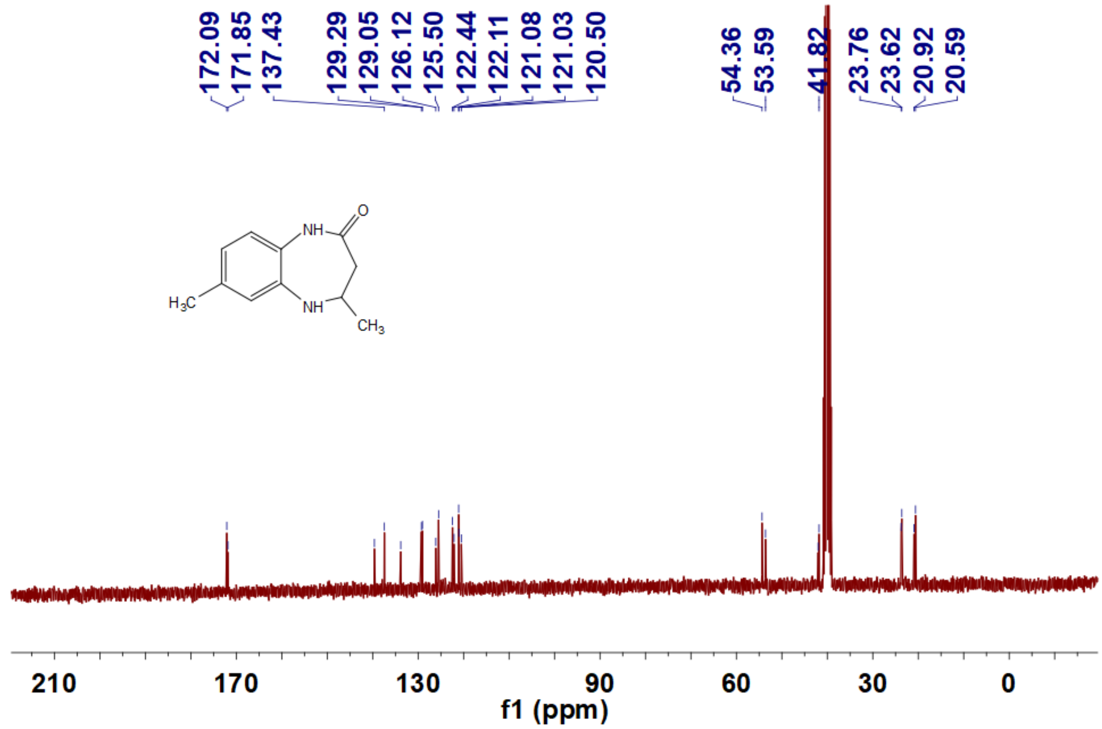

4,7,8-Trimethyl-1,3,4,5-tetrahydro-2*H*-benzo[b][1,4]diazepin-2-one (3v)

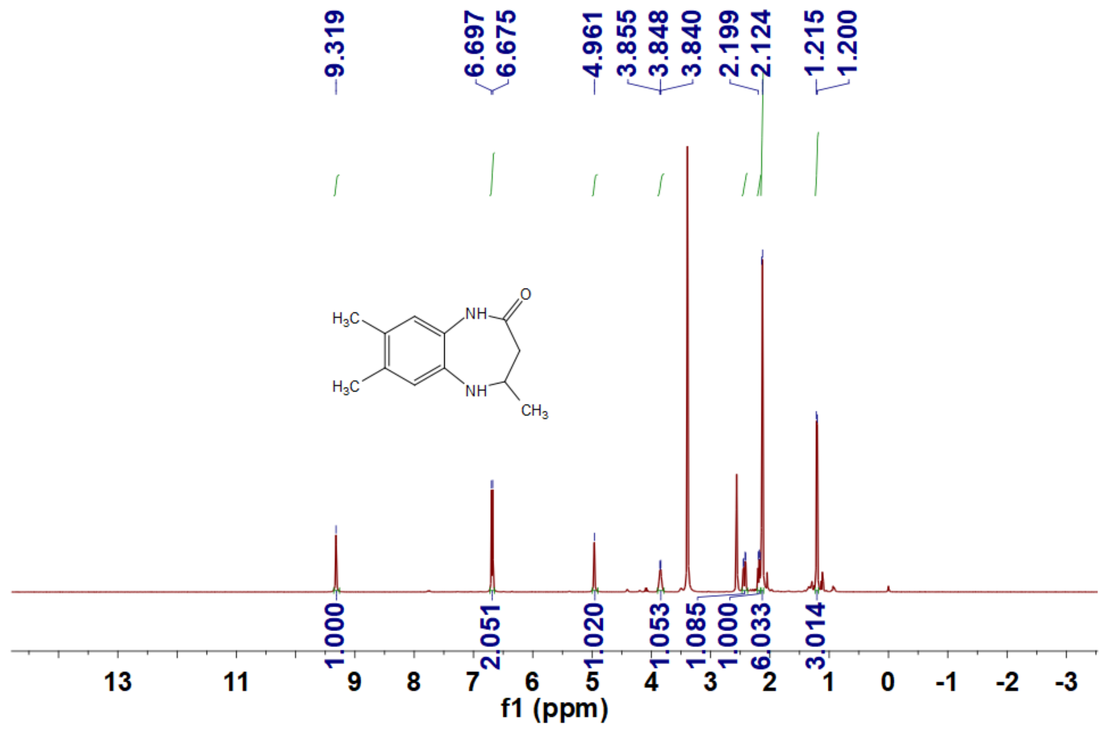

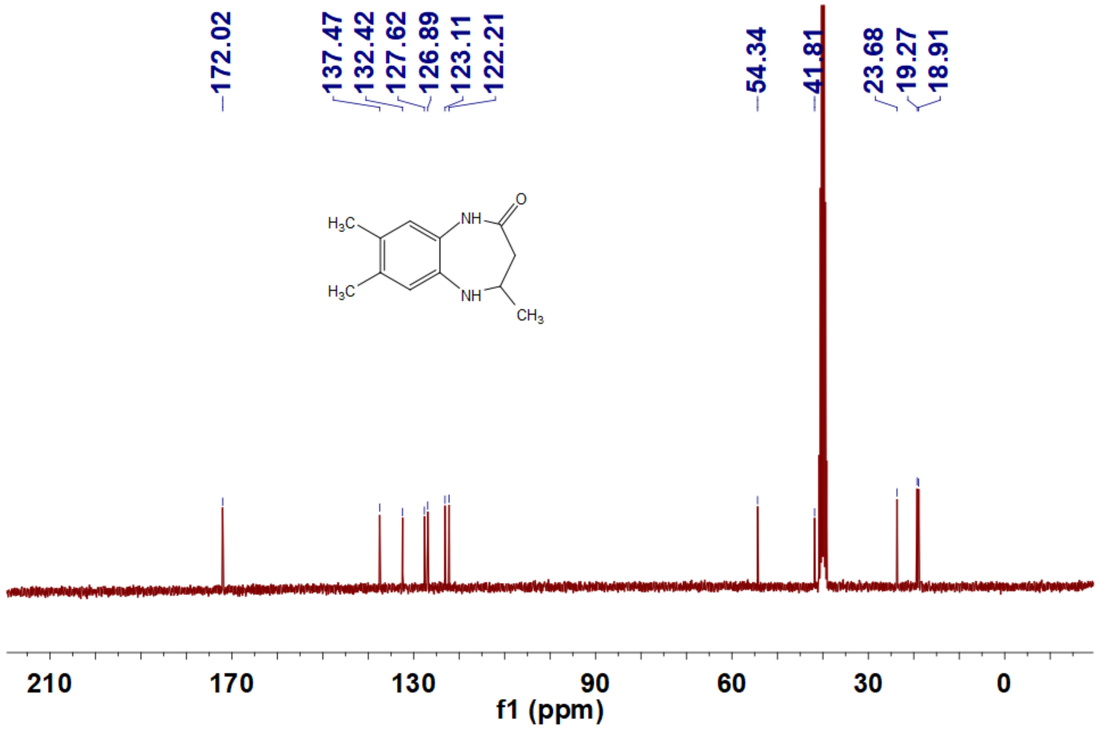

7-Methoxy-4-methyl-1,3,4,5-tetrahydro-2*H*-benzo[*b*][1,4]diazepin-2-one (3w)

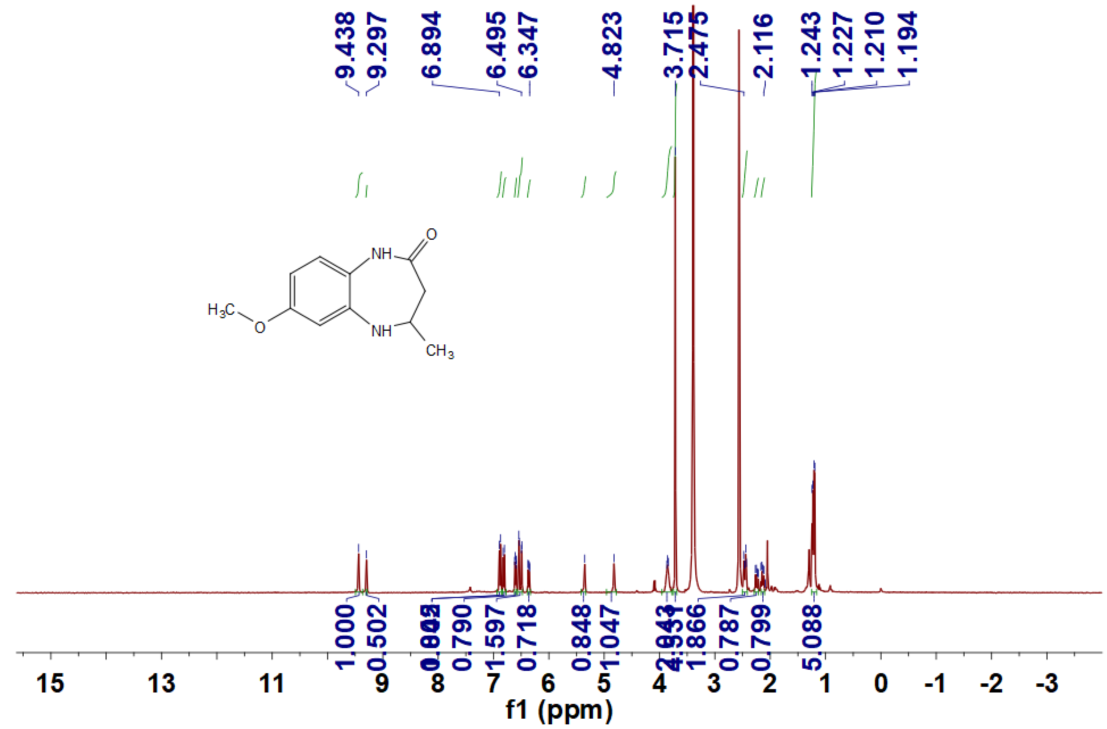

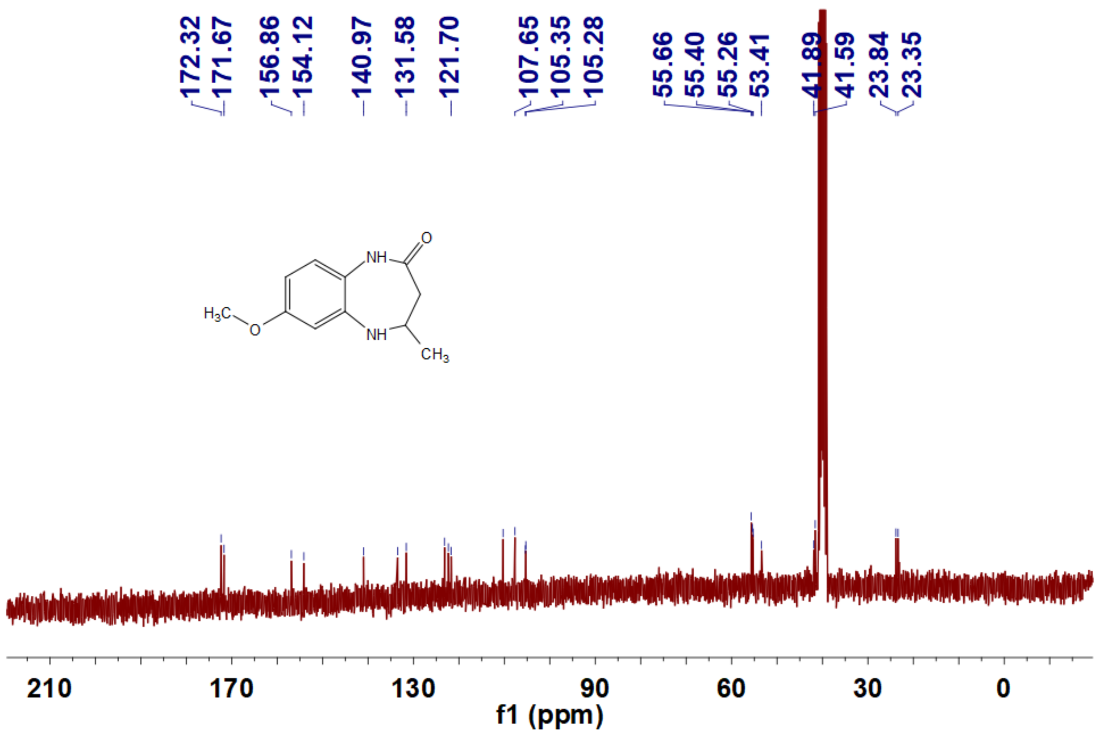

4-Methyl-1,3,4,5-tetrahydro-2*H*-naphtho[2,3-*b*][1,4]diazepin-2-one (3x)

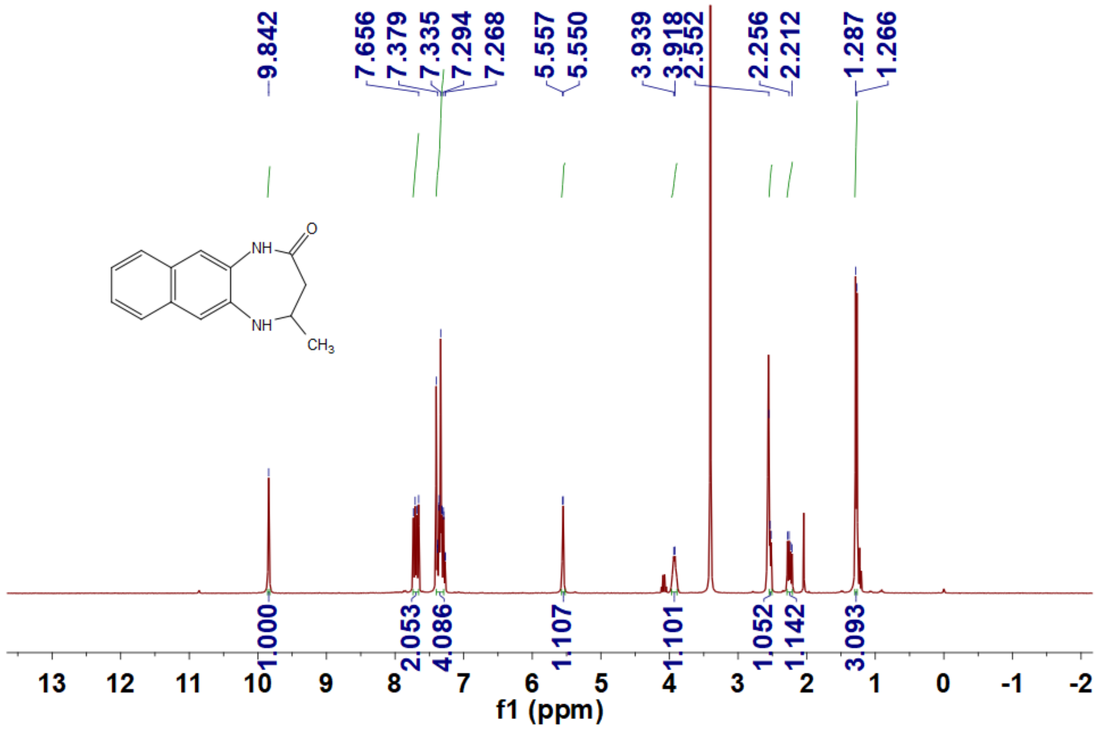

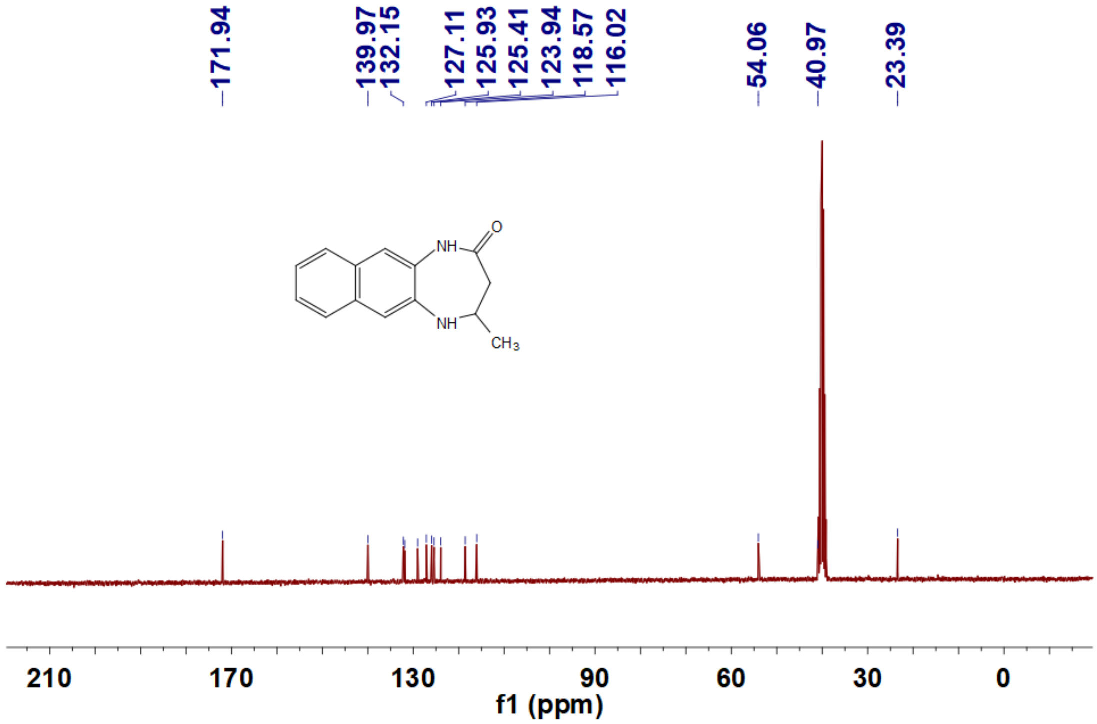

5-Methyl-2-(naphthalen-2-yl)pyrazolidin-3-one (3aa)

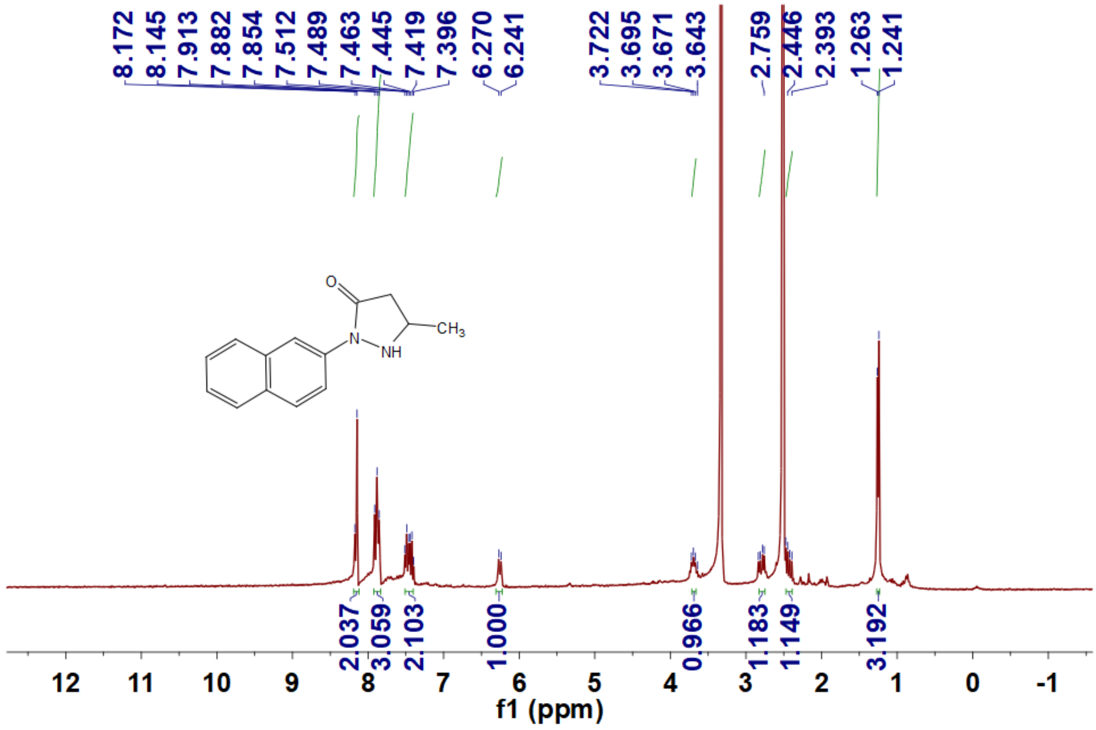

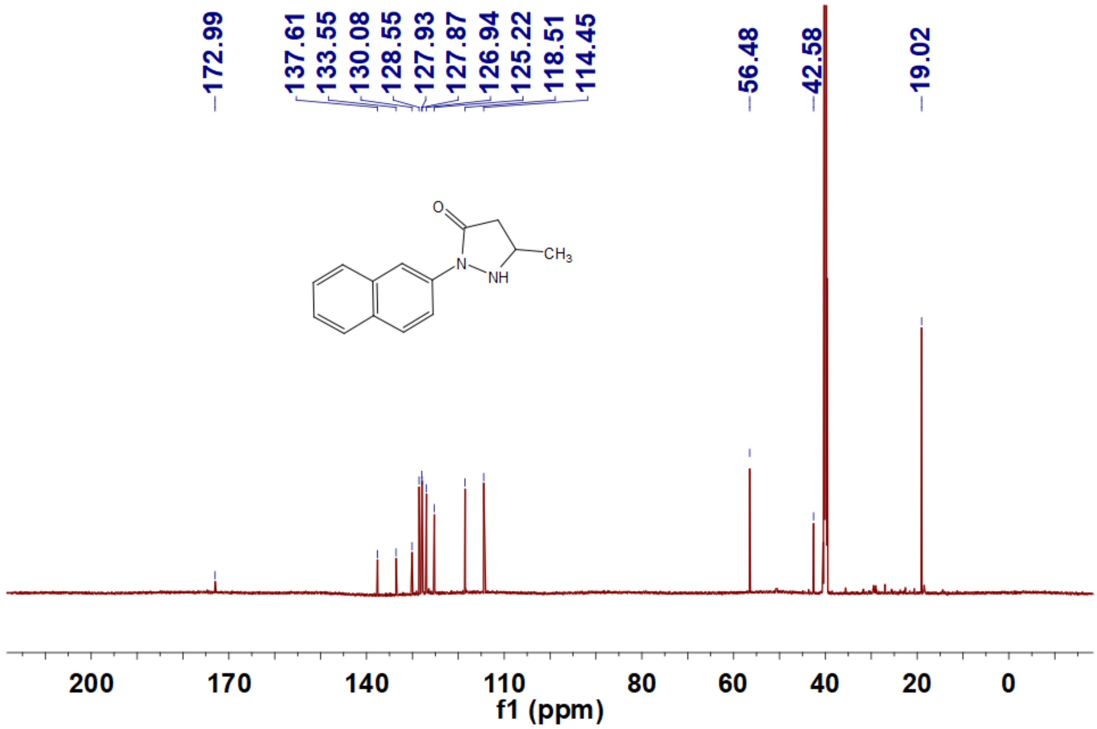

5-Methylpyrazolidin-3-one (3ab)

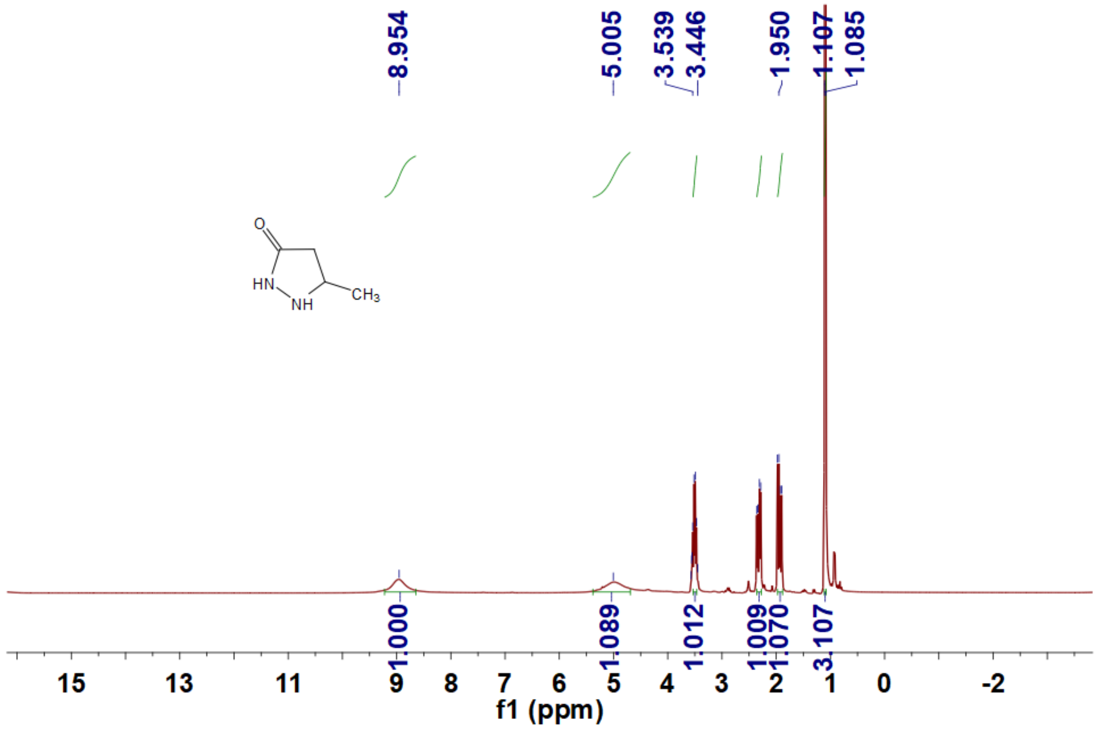

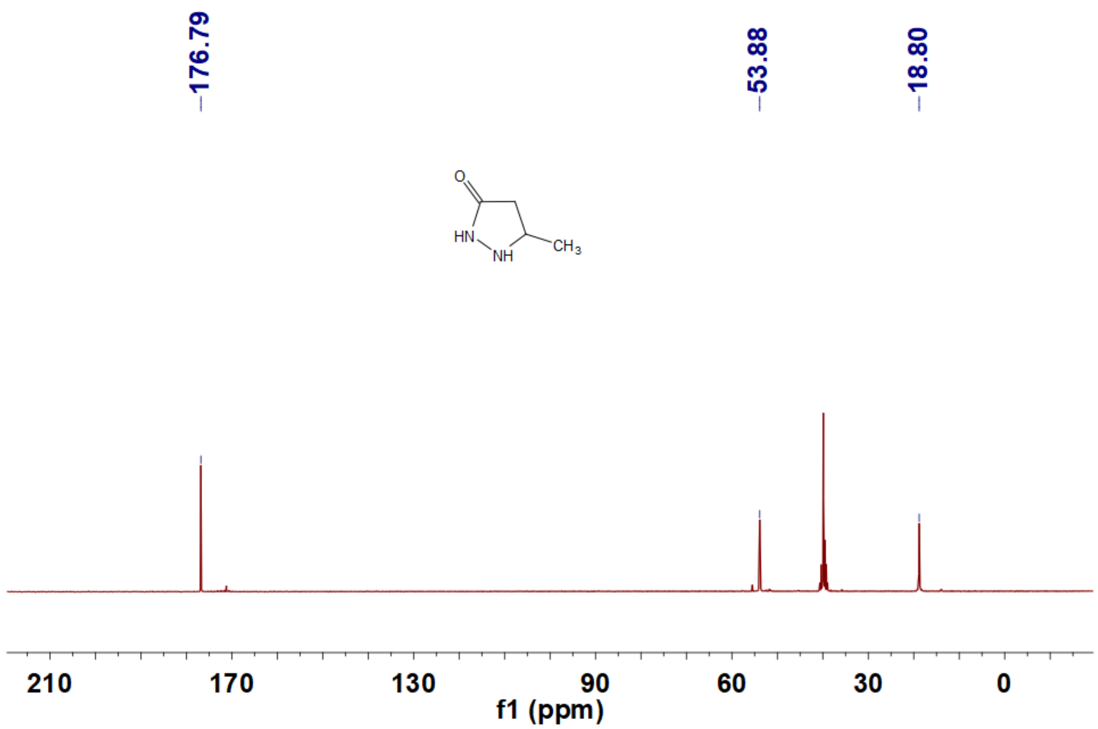

5-Methyl-2-phenylpyrazolidin-3-one (3ac)

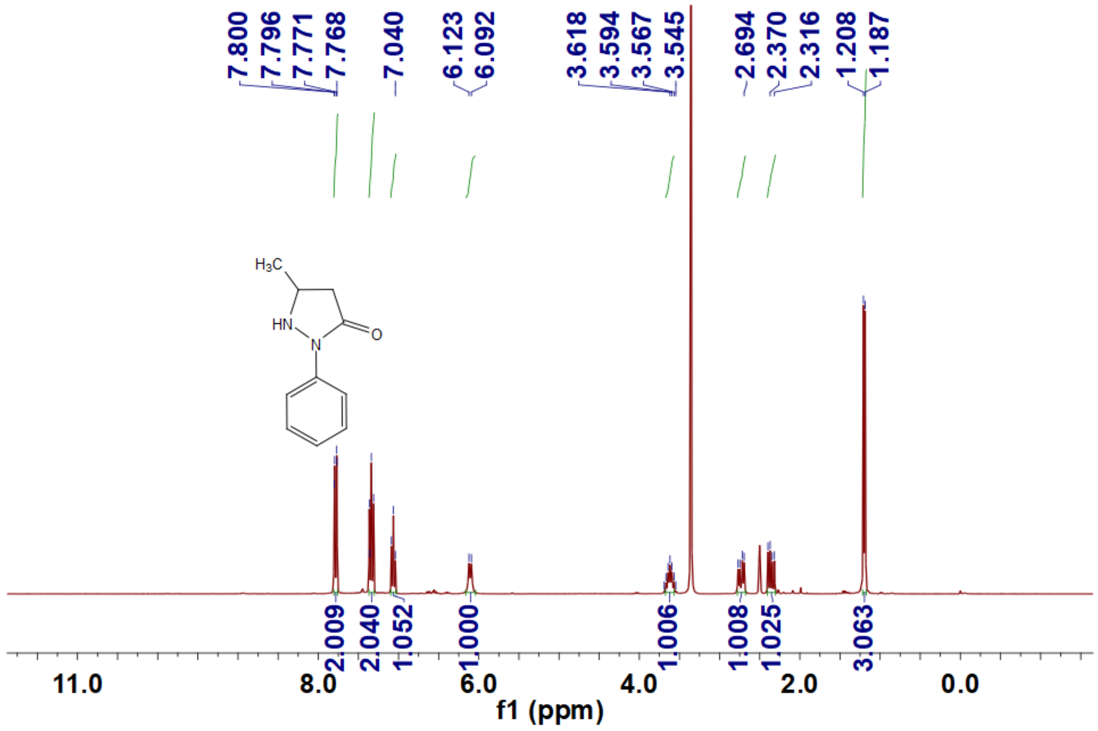

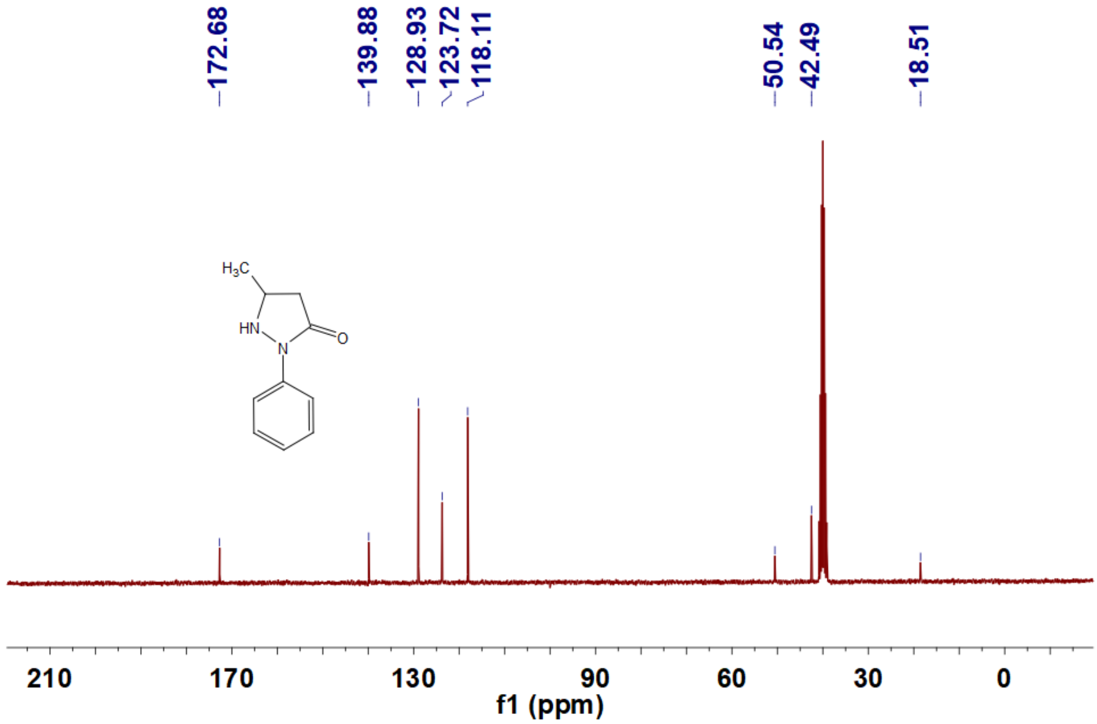

5-Methyl-2-(*p*-tolyl)pyrazolidin-3-one (3ad)

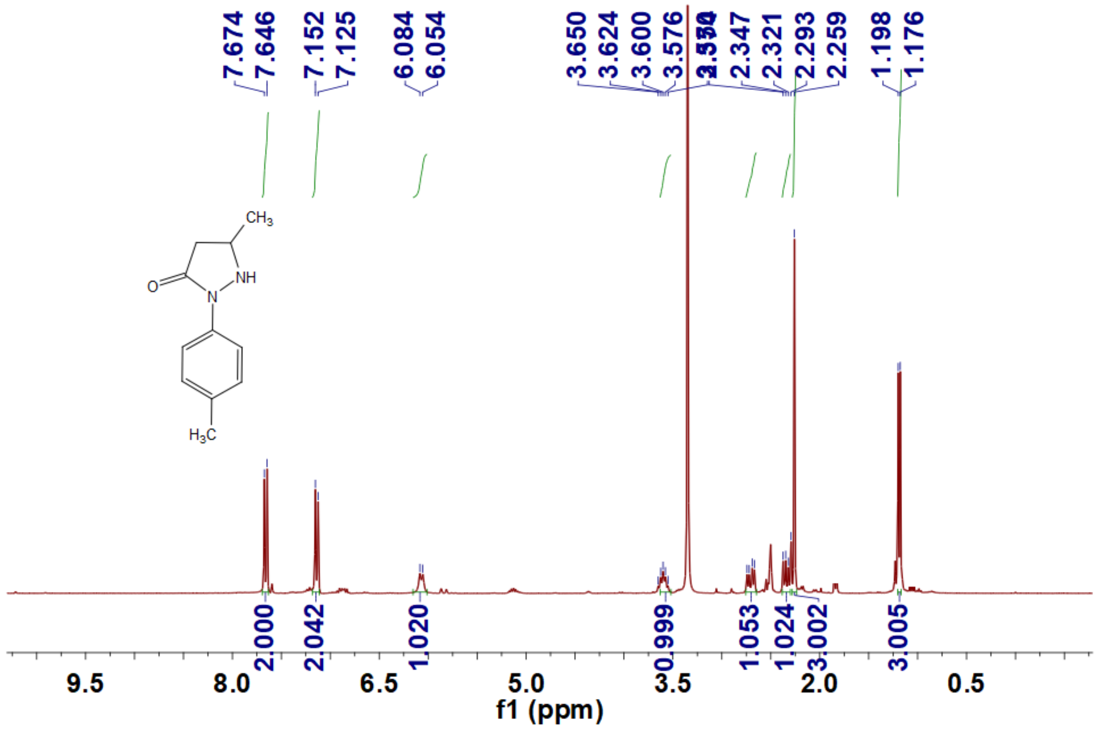

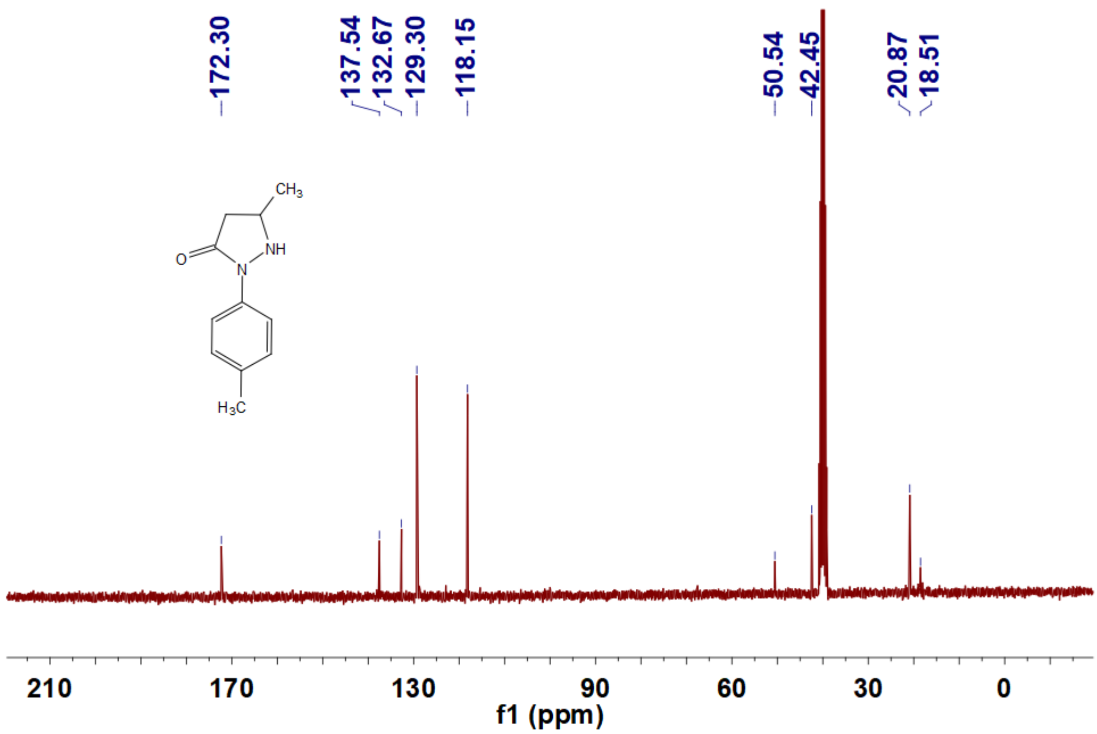

5-Methyl-2-(*m*-tolyl)pyrazolidin-3-one (3ae)

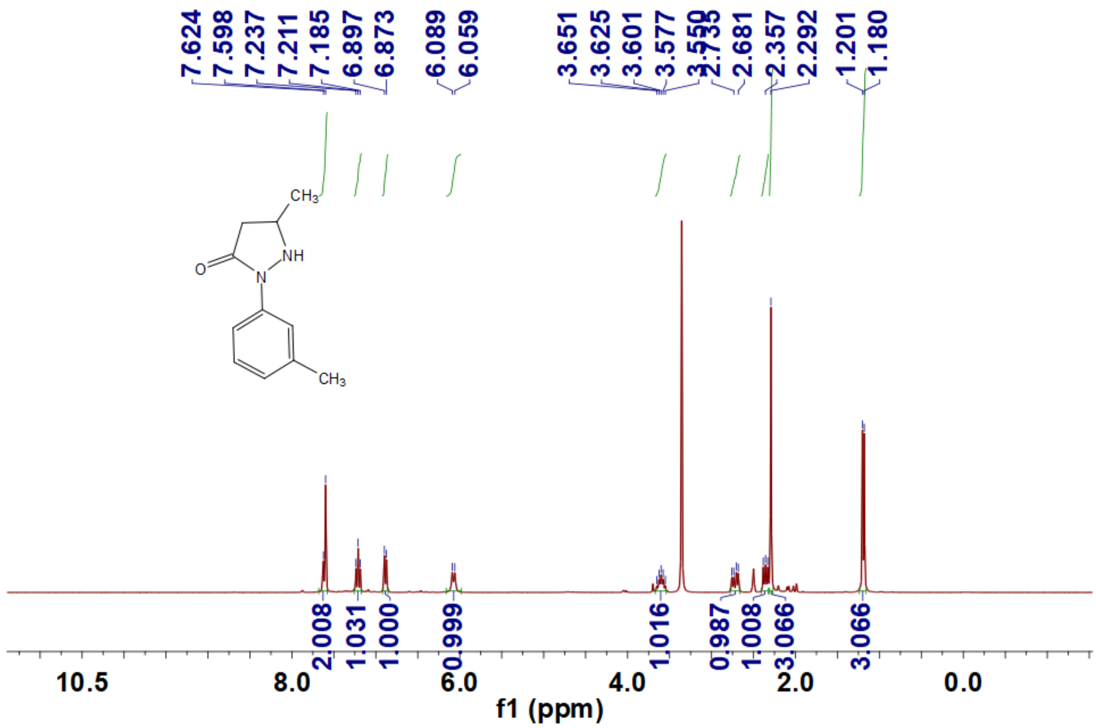

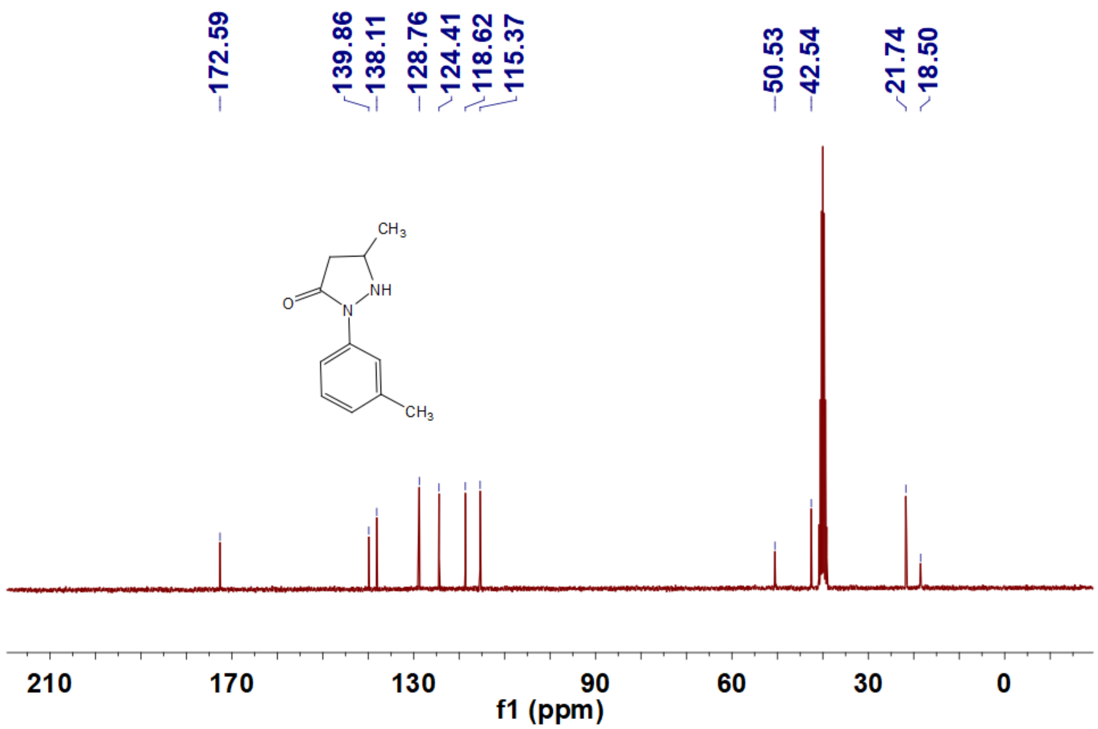

2-(4-Methoxyphenyl)-5-methylpyrazolidin-3-one (3af)

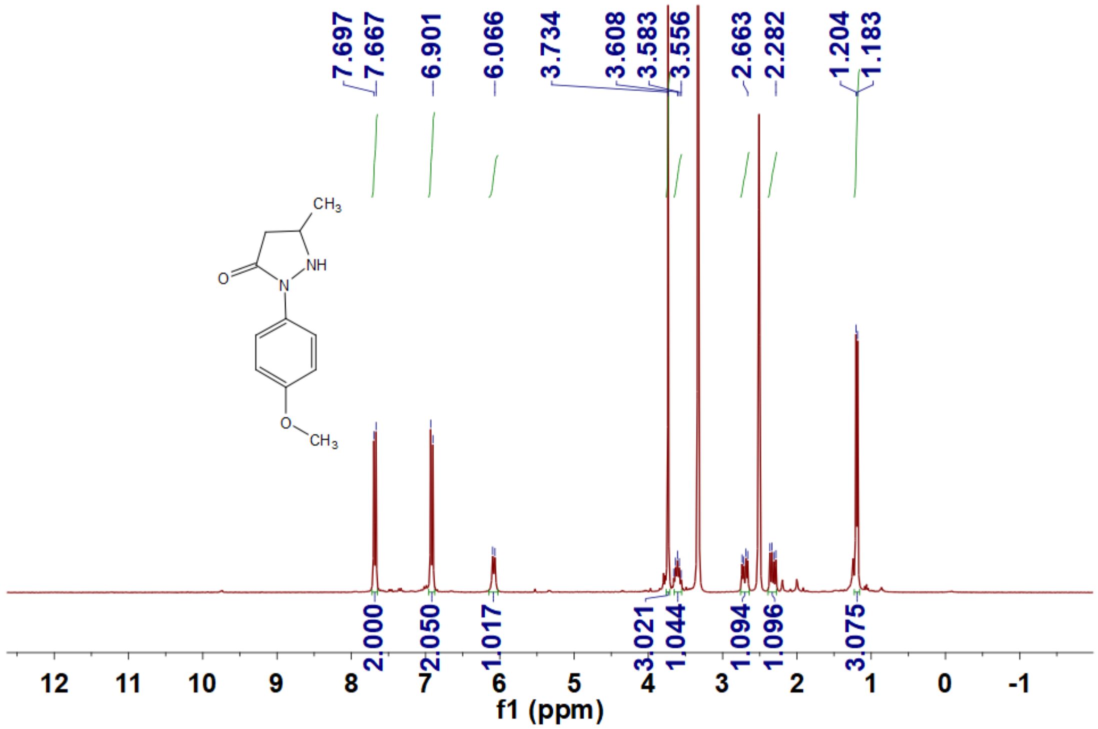

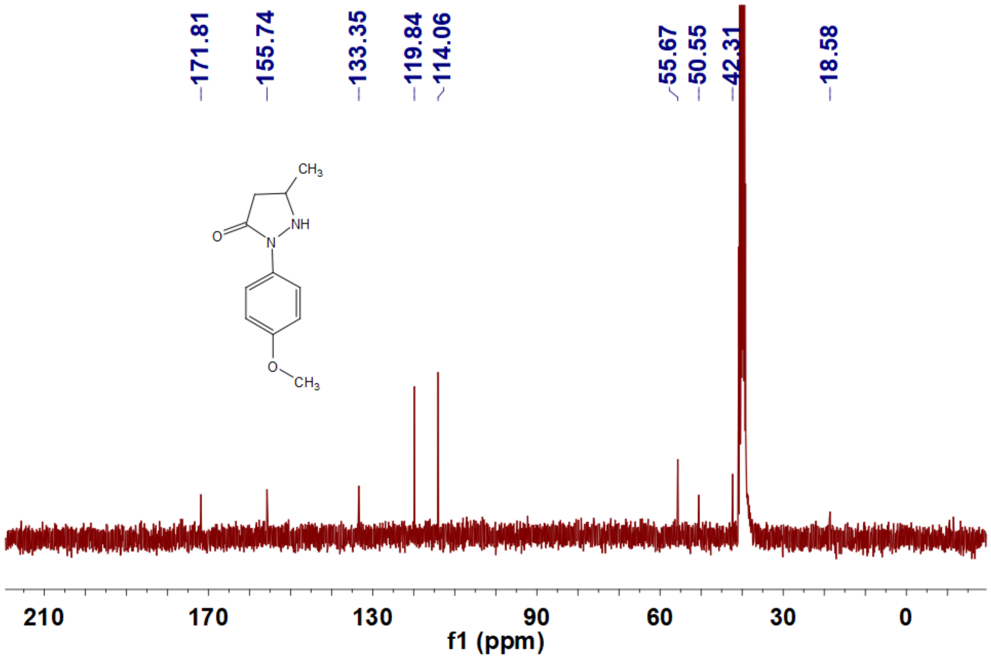

2-(4-Fluorophenyl)-5-methylpyrazolidin-3-one (3ag)

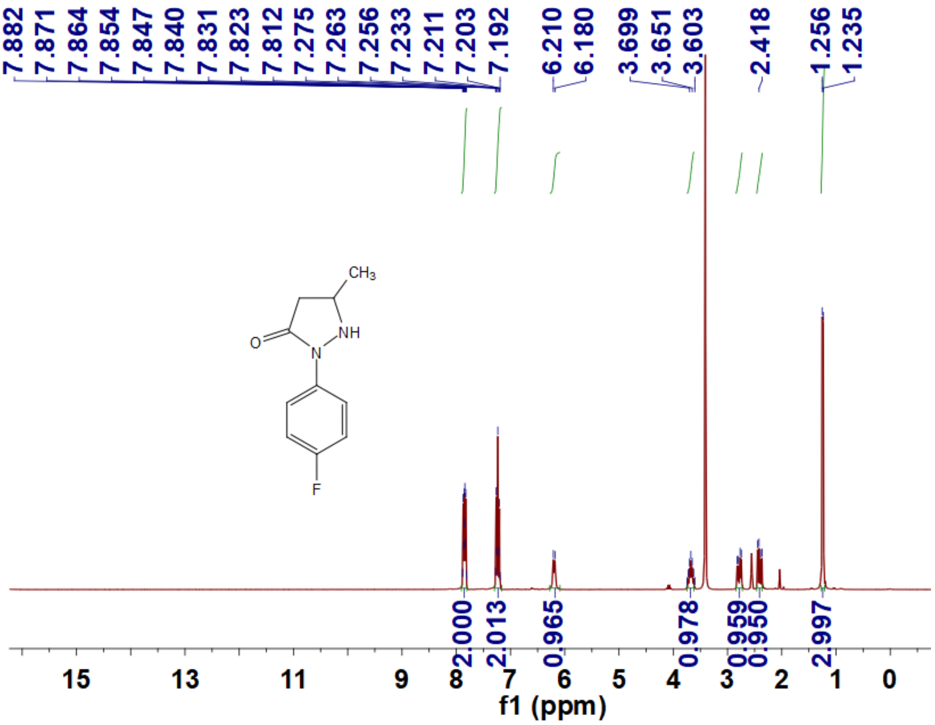

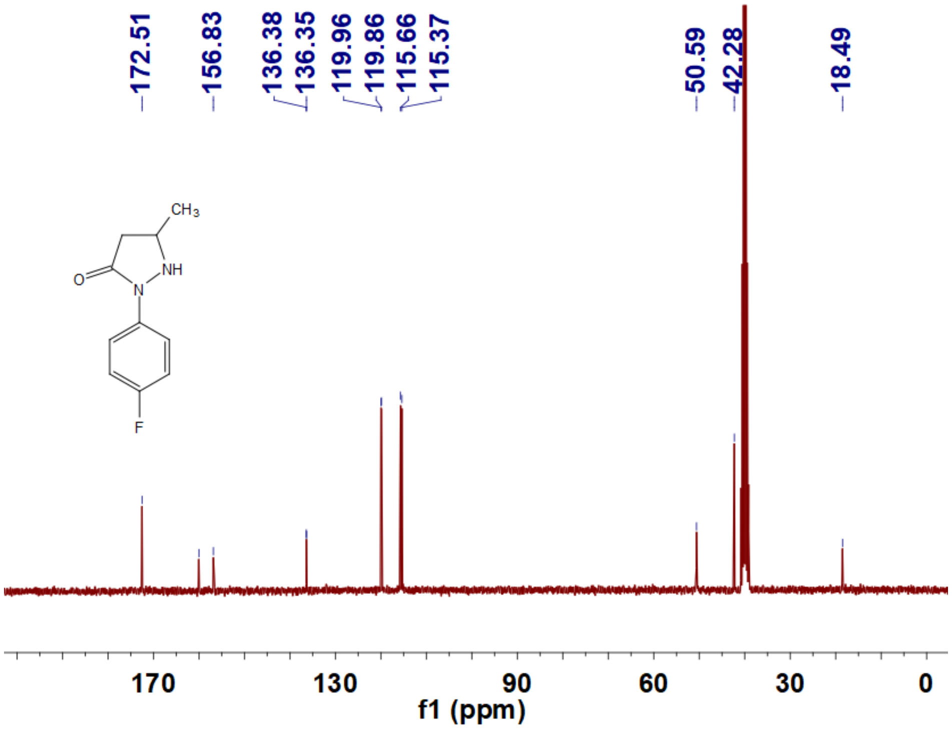
172.51
156.83
136.38
136.35
119.96
119.86
115.66
115.37
50.59
42.28
18.49
CH3
O
N
NH
F
170
130
90
60
30
0
f1 (ppm)

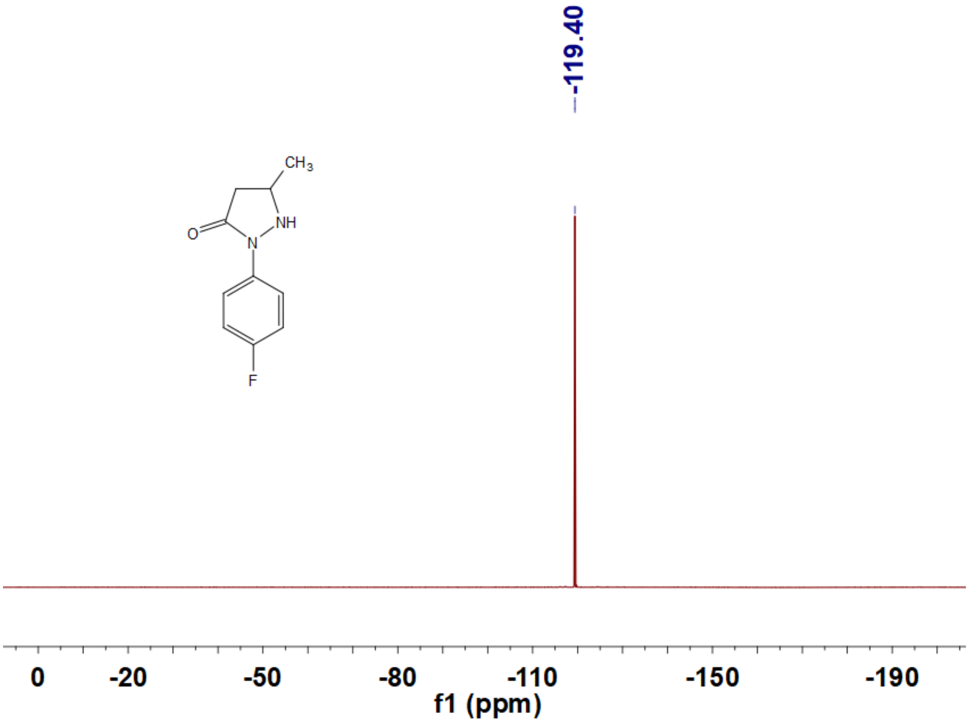
-119.40
CH3
O
N
NH
F
0
-20
-50
-80
-110
-150
-190
f1 (ppm)

2-(4-Chlorophenyl)-5-methylpyrazolidin-3-one (3ah)

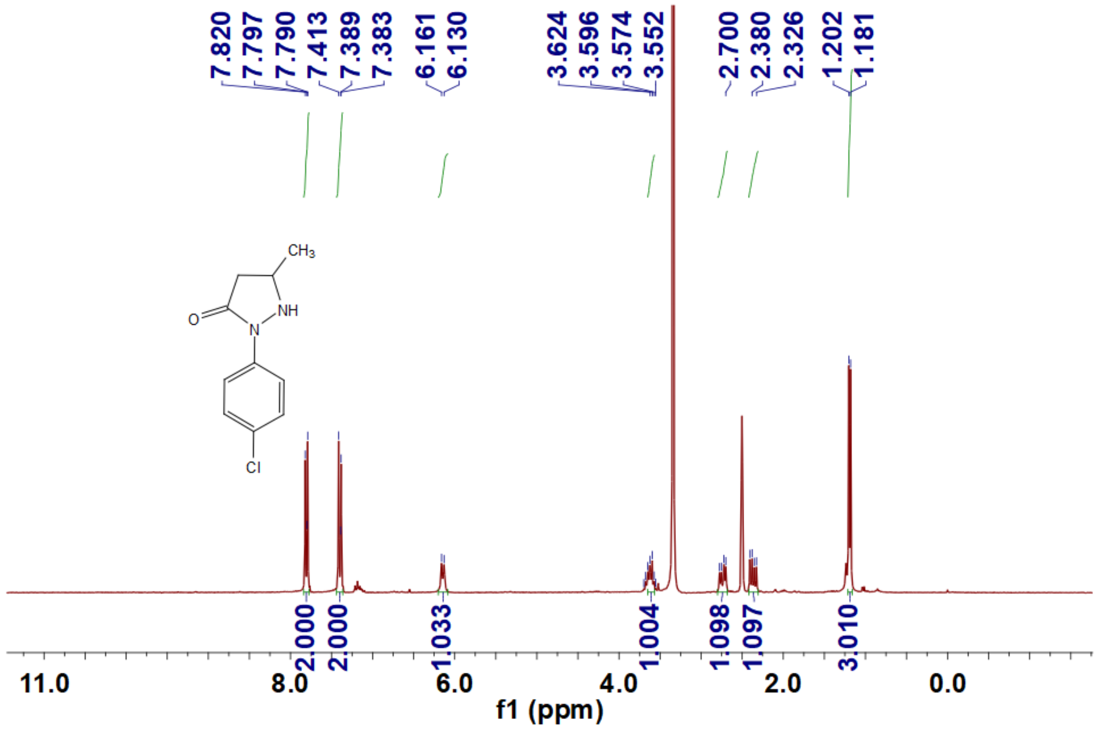

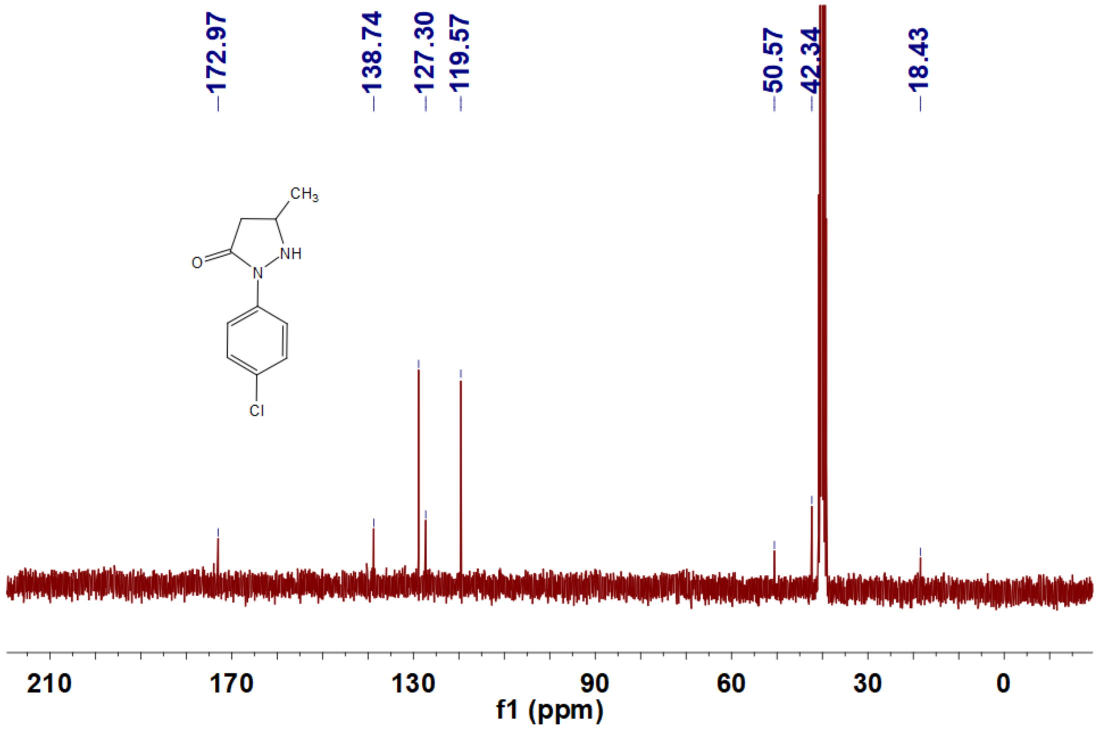

2-(4-Bromophenyl)-5-methylpyrazolidin-3-one (3ai)

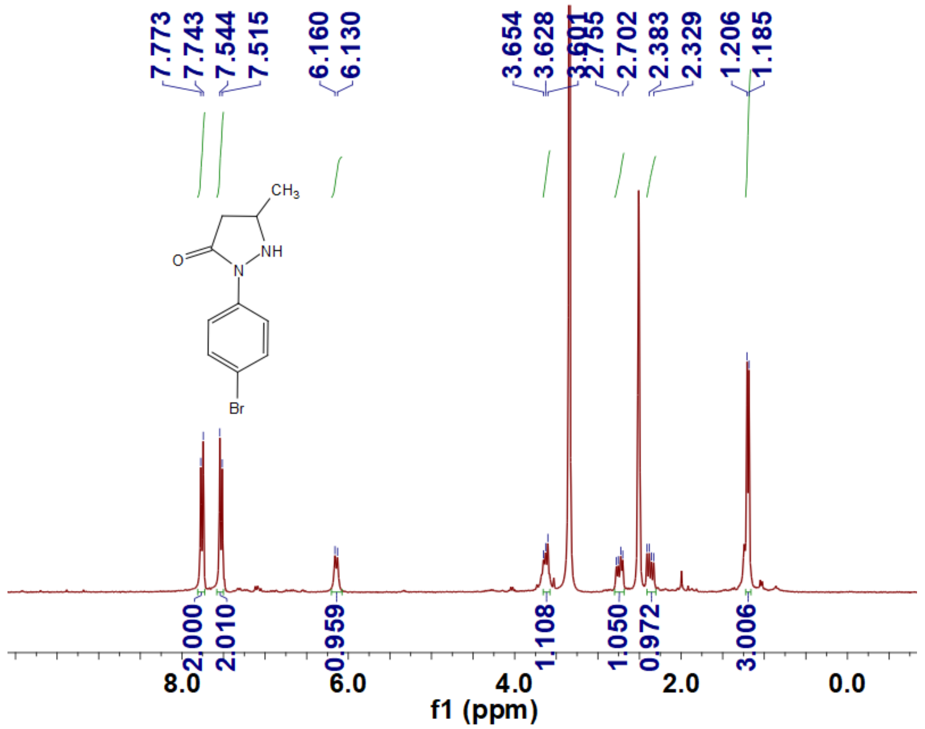

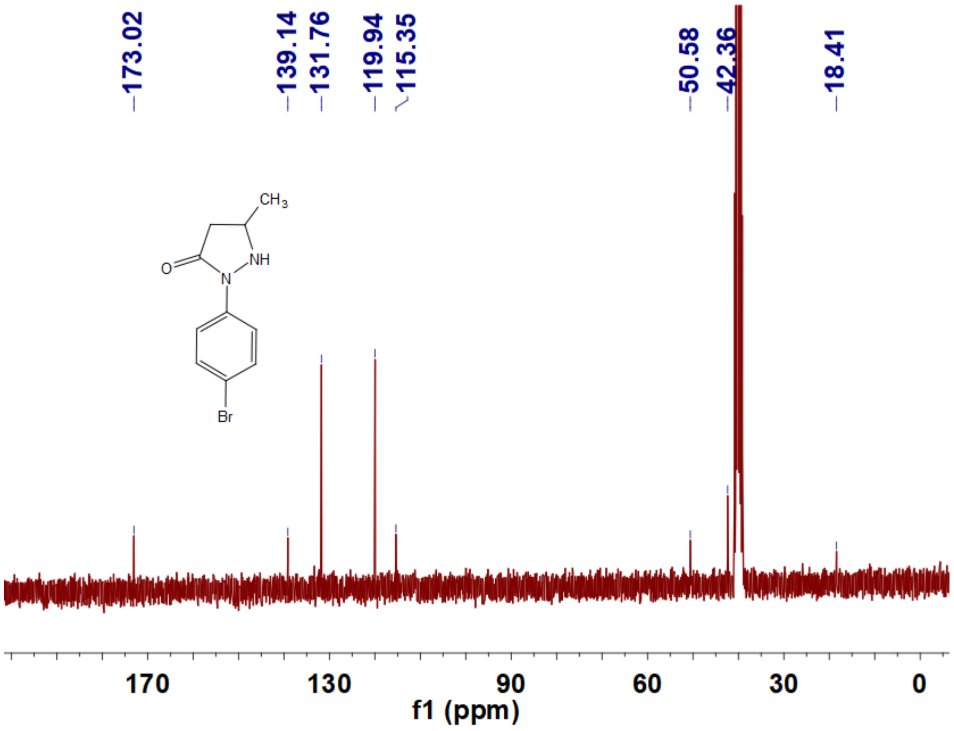

2-(4-Iodophenyl)-5-methylpyrazolidin-3-one (3aj)

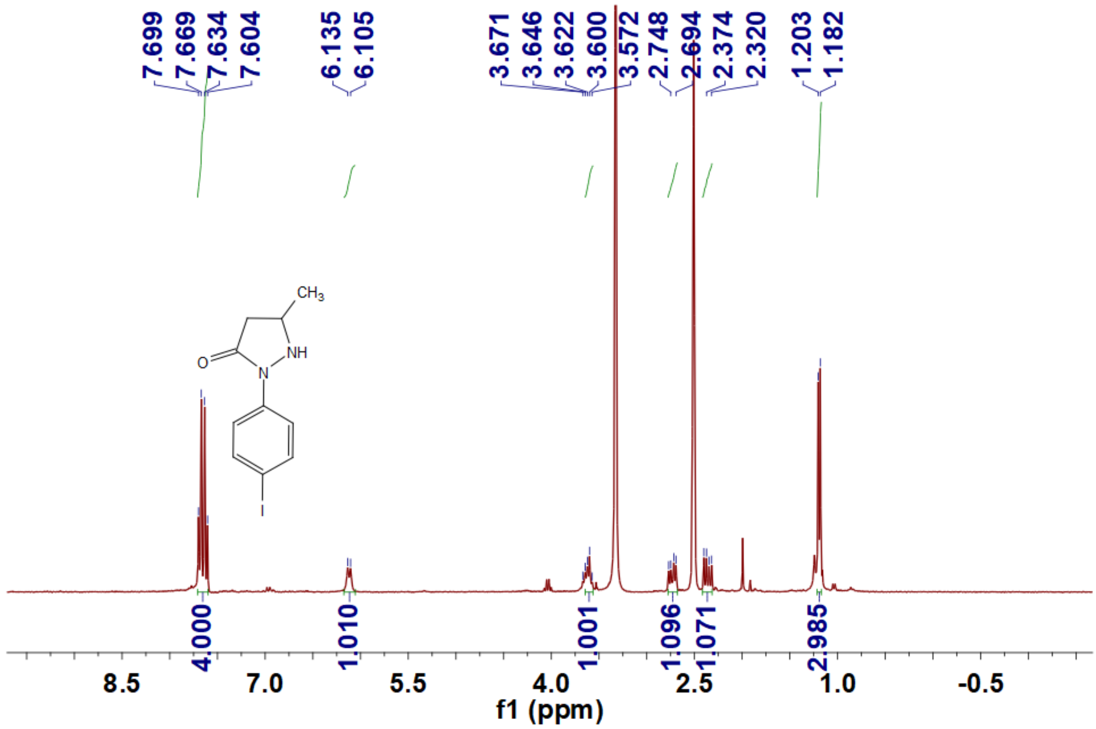

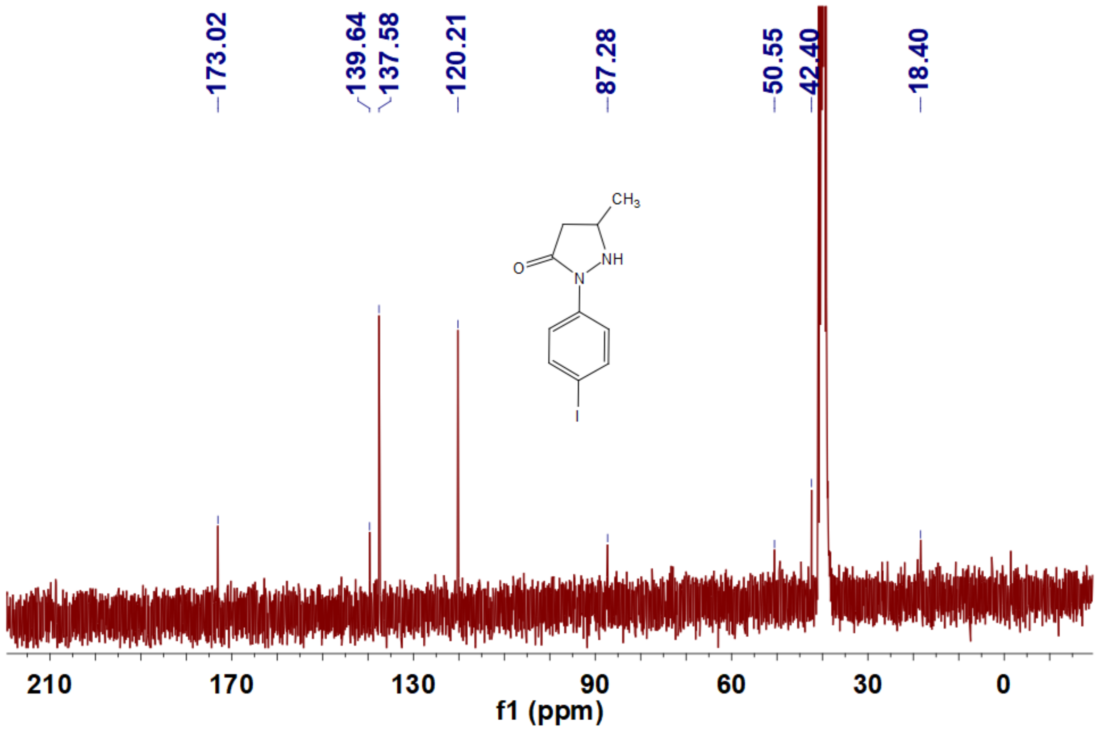

Supplement: SC-015-D4SC02533D-s001 [file SC-015-D4SC02533D-s001.pdf]
